# Pathways of positive youth development, hope, and life satisfaction: A multi‐country study among Southeast Asian youth

**DOI:** 10.1111/jora.70313

**Published:** 2026-11-05

**Authors:** Jet Uy Buenconsejo, Laura Ferrer‐Wreder, Lu Yu, Steven Krauss, Nor Ba'yah Abdul‐Kadir, Yue Yu, John Jamir Benzon R. Aruta, Angela Oktavia Suryani, Priyoth Kittiteerasack, Duong Cong Doanh, Radosveta Dimitrova

**Affiliations:** ^1^ Department of Applied Social Sciences The Hong Kong Polytechnic University Hong Kong SAR China; ^2^ Department of Psychology Stockholm University Stockholm Sweden; ^3^ Department of Psychology University of Agder Kristiansand Norway; ^4^ Department of Human Development and Family Science University of Missouri Columbia Missouri USA; ^5^ Centre for Research in Psychology and Human Well‐being, Faculty of Social Sciences and Humanities Universiti Kebangsaan Malaysia Bangi Selangor Malaysia; ^6^ Singapore Centre for Character and Citizenship Education National Institute of Education, Nanyang Technological University Singapore City Singapore; ^7^ Centre for Research in Child Development National Institute of Education, Nanyang Technological University Singapore City Singapore; ^8^ Department of Psychology De La Salle University Manila Philippines; ^9^ Faculty of Psychology Atma Jaya Catholic University of Indonesia Jakarta Indonesia; ^10^ Department of Mental Health and Psychiatric Nursing Thammasat University Pathum Thani Thailand; ^11^ Faculty of Business Management National Economics University Hanoi Vietnam

**Keywords:** emerging adults, hope, life satisfaction, positive youth development, Southeast Asia, well‐being

## Abstract

Positive Youth Development (PYD) has gained prominence through models such as the 7Cs—competence, confidence, connection, character, caring, contribution, and creativity—yet relatively little is known about how these dimensions function in Southeast Asian contexts, where young people navigate diverse cultural values, socio‐economic structures, and access to resources. Further, although PYD attributes are linked to life satisfaction, limited work has explored mechanisms that may explain these associations. This study investigated the direct and indirect relations of the 7Cs with life satisfaction via hope among youth from six Southeast Asian countries. Participants included 1967 emerging adults (aged 18–25) from Indonesia, Malaysia, the Philippines, Singapore, Thailand, and Vietnam. A series of confirmatory factor analyses and structural equation modeling were conducted to examine the direct and indirect associations of the 7Cs with life satisfaction via hope, while accounting for gender, age, and education. Controlling for the covariates, results showed that confidence, connection, and creativity had indirect associations with life satisfaction through hope. Country‐specific models revealed culturally distinct associations, such as the significant indirect role of character in the Philippines, and the nonsignificant relations of confidence and creativity with well‐being outcomes in Thailand and Singapore, respectively. Together, these findings advance the understanding of the 7Cs in Southeast Asia, highlight hope as an important mechanism linking PYD to life satisfaction, and underscore the importance of culturally relevant approaches to youth development in the region.

Positive Youth Development (PYD) provides a strengths‐based framework for understanding how young people thrive by harnessing individual capacities, supportive relationships, and contextual resources (Lerner et al. 2010). Among existing PYD models, the 7Cs—competence, confidence, connection, character, caring, contribution, and creativity—offer a comprehensive framework of youth strengths spanning intrapersonal, interpersonal, and ecological domains (Dimitrova et al. 2021). Empirical research has consistently linked PYD attributes to adaptive developmental outcomes, including prosocial behaviors, academic performance, and life satisfaction (Carvalho et al. 2024; Geldhof et al. 2014; Holsen et al. 2017). However, much of this evidence is derived from Western youth samples, raising concerns about the applicability of PYD frameworks in Majority World regions where cultural values, social structures, and developmental affordances differ substantially (Koller & Verma, 2017; Wiium & Dimitrova, 2019).

Beyond issues of representation, key conceptual gaps remain in the PYD literature. First, PYD is typically operationalized as a global construct or limited only to the 5Cs, obscuring the distinct contributions of other PYD dimensions, particularly contribution and creativity (Buenconsejo, Ferrer‐Wreder, Dimitrova, et al. 2026). Second, although PYD characteristics are strongly associated with life satisfaction (Holsen et al. 2017), few studies have examined the psychological mechanisms that may facilitate these associations. Hope represents a theoretically grounded mechanism, as it reflects goal‐directed agency, optimistic pathways of thinking, and relational support, processes that are closely aligned with PYD resources (Bowers & Bowers, 2023; Callina et al. 2015). Preliminary evidence suggests that hope mediates the associations between PYD and well‐being (Abdul‐Kadir & Mohd, 2021), yet its role in linking the broader 7Cs framework to life satisfaction remains largely unexplored, particularly in understudied non‐Western contexts.

These gaps are particularly salient in Southeast Asia, a demographically significant (12% of the world's youth population) and culturally diverse region generally characterized by a strong collectivistic orientation and substantial cross‐national variations in youth development (ASEAN, 2023; Rattanasevee, 2023). Emerging research indicates that interpersonal and socio‐emotional PYD dimensions, particularly connection, may be more salient for well‐being in societies with an interdependent orientation, while other Cs may function differently (Buenconsejo, Datu, & Liu 2025; Wong et al. 2021). To address these limitations, this multi‐country study examined the direct and indirect associations between the 7Cs of PYD, hope, and life satisfaction among emerging adult samples from six Southeast Asian countries including Indonesia, Malaysia, the Philippines, Thailand, Singapore, and Vietnam. By testing the mediating role of hope and examining sample‐specific associations, this study aimed to provide a more comprehensive and contextually sensitive understanding of youth thriving in these portions of the Majority World.

## THE POSITIVE YOUTH DEVELOPMENT (PYD) FRAMEWORK

The Positive Youth Development (PYD) framework represents an alternative lens to deficit‐oriented developmental perspectives that have historically conceptualized young people as problems to be managed or patients to be cured (Lerner et al. 2005, 2010). Grounded in relational developmental systems theories and informed by principles of positive psychology, PYD conceptualizes youth as active agents whose strengths, capacities, and contextual resources jointly shape developmental trajectories toward thriving (Buenconsejo & Datu, 2022; Tolan et al. 2016). Rather than focusing solely on the prevention of risks and maladjustments, PYD emphasizes the identification and promotion of psychosocial resources that promote well‐being and positive contributions to society (Geldhof et al. 2014). Over the past two decades, this strengths‐based perspective has gained substantial empirical and applied momentum, leading to the development of several conceptual models of youth thriving, including the 40 developmental assets framework, the 15 PYD constructs, and the 5/6/7Cs models (see Buenconsejo & Datu, 2022; Qi et al. 2020).

Among these approaches, the 7Cs model of PYD offers a comprehensive and developmentally grounded framework for capturing multidimensional strengths (Dimitrova et al. 2021). Extending the original 5/6Cs model (Geldhof et al. 2014; Lerner et al. 2005, 2010), the 7Cs model incorporates creativity alongside competence, confidence, connection, character, caring, and contribution (Dimitrova et al. 2021). Specifically, *competence* reflects positive evaluations of one's abilities across academic, social, and career domains, whereas *confidence* denotes a more global sense of self‐worth and self‐efficacy. *Connection* captures the presence of supportive and mutually beneficial relationships with peers, family members, and institutions. *Character* refers to moral integrity, respect for social and cultural norms, and a coherent ethical orientation, while *caring* reflects empathy and compassion for others. *Contribution* pertains to young people's active engagement in behaviors that benefit other people and their communities. Lastly, *creativity* is defined as the capacity to generate original, useful, and contextually adaptive solutions to problems, reflecting youths' ability to navigate complexity and change (Dimitrova et al. 2021; Said‐Metwaly et al. 2017).

Compared with earlier PYD frameworks, the 7Cs model offers a more comprehensive account of youth thriving by incorporating both contribution and creativity, two developmental assets that are particularly salient during emerging adulthood. Prior studies have supported the factor structure of the 7Cs of PYD among diverse youth samples (Abdul Kadir et al. 2021; Buenconsejo, Ferrer‐Wreder, Dimitrova, et al. 2026; Manrique‐Millones et al. 2021). As young people transition into higher education, employment, and broader civic roles, opportunities to contribute to communities and address increasingly complex challenges become more central to their developmental experiences (Arnett, 2024; Nelson, 2021). Creativity may be especially relevant in Southeast Asian contexts, where emerging adults are navigating rapid technological change, evolving labor market demands, and diverse sociocultural environments that require flexibility, innovation, and adaptive problem‐solving (ASEAN, 2023; Rattanasevee, 2023). By encompassing both prosocial engagement and creative adaptation, the 7Cs framework provides a broader lens for understanding how emerging adults thrive within contemporary and rapidly changing societies (Dimitrova et al. 2021).

A useful framework for understanding the 7Cs and other PYD indicators is through the integrative paradigm of PYD, which classifies thriving dimensions into three interrelated clusters: intrapersonal‐immediate, interpersonal‐proximal, and ecological‐distal (Buenconsejo & Datu, 2022, 2023a). *Intrapersonal‐immediate indicators* (e.g., competence, confidence, creativity) reflect self‐focused strengths that directly benefit the individual. *Interpersonal‐proximal indicators* (e.g., connection and caring) emphasize supportive relational processes. *Ecological–distal indicators* (e.g., character and contribution) capture values, moral orientations, and prosocial behaviors that extend beyond proximal relationships and benefit broader social systems (Buenconsejo & Datu, 2022). Although these dimensions have each been linked to psychological well‐being, existing research has largely examined PYD as an aggregate construct or focused on limited subsets of the Cs, leaving the distinct pathways linking specific PYD characteristics to well‐being outcomes insufficiently understood. Furthermore, such mechanisms remain particularly underexplored in Majority World contexts, where cultural values, social structures, and developmental affordances may shape the functioning of PYD attributes in distinct ways. Addressing these gaps requires simultaneously examining multiple PYD dimensions and their associations with well‐being outcomes across diverse youth samples.

## PYD, HOPE, AND LIFE SATISFACTION

The PYD framework posits that young people who exhibit thriving characteristics are more likely to experience optimal developmental outcomes and fewer maladaptive behaviors (Lerner et al. 2005, 2010). Past empirical evidence supports this proposition across different well‐being outcomes. For example, PYD attributes have been positively associated with psychological empowerment (Holsen et al. 2017), academic achievement (Kozina et al. 2019), grit and school engagement (Buenconsejo, Datu, & Liu 2025), contribution (Yang & McGinley, 2021), and prosociality (Buenconsejo et al. 2024). Conversely, PYD dimensions have demonstrated inverse relations with maladaptive outcomes, including anxiety and depressive symptoms (Geldhof et al. 2014), suicidal ideation (Wong et al. 2021), and emotion dysregulation (Dvorsky et al. 2019). Collectively, these findings affirm PYD as a robust developmental framework linking intrapersonal, interpersonal, and ecological assets to well‐being, while also underscoring the importance of examining specific PYD dimensions rather than treating PYD as a unidimensional construct.

Life satisfaction represents a key well‐being outcome within the PYD literature (Tolan et al. 2016). As the cognitive component of subjective well‐being, life satisfaction refers to individuals' global evaluation of their quality of life based on self‐determined criteria, reflecting feelings of contentment, fulfillment, and overall happiness (Kjell & Diener, 2021). Empirical studies, largely focused on the 5Cs, consistently support positive associations between PYD characteristics and life satisfaction among youth samples from Western societies (Carvalho et al. 2024; Holsen et al. 2017). For instance, among Norwegian adolescents, all 5Cs were positively associated with life satisfaction, with competence, confidence, and connection showing the strongest associations, followed by weaker, but still significant, relations for character and caring (Holsen et al. 2017). The same dimensions of competence, confidence, and connection were also found to be positively associated with life satisfaction among a sample of Portuguese adolescents (Carvalho et al. 2024).

Evidence from non‐Western and Southeast Asian youth samples, however, reveals a more complex pattern. In a large study of Chinese secondary school students in Hong Kong, connection and character emerged as the strongest correlates of life satisfaction, whereas competence and confidence showed comparatively weaker associations (Wong et al. 2021). Notably, connection was robustly linked to multiple domains of life satisfaction and was associated with reduced suicidal ideation, highlighting the salience of relational and moral dimensions of PYD in a context characterized by a relatively stronger collectivistic orientation (Wong et al. 2021). Similarly, among Filipino adolescents during the COVID‐19 pandemic, PYD was positively associated with life satisfaction, with connection again demonstrating the strongest relation, followed by confidence and competence, and then character and caring (Buenconsejo et al. 2022). Taken together, these findings suggest that while connection consistently emerges as a central predictor of life satisfaction across cultures, the relative importance of the other Cs tends to vary across contexts.

Beyond direct associations, understanding how PYD attributes relate to life satisfaction necessitates the examination of intervening psychological processes, such as hope. Hope is defined as a positive psychological construct reflecting optimistic expectations of achieving desired outcomes through realistic goals and supportive relationships (Herth, 1992). Conceptually, hope integrates intentional self‐regulation, positive future expectations, and connectedness, enabling young people to set, pursue, and attain meaningful goals in life (Callina et al. 2015; Herth, 1992). From a PYD perspective, hope aligns closely with adaptive person–context interactions and is nurtured through psychosocial resources embedded in family, school, and community contexts (Bowers & Bowers, 2023). Considering the beneficial impacts of PYD attributes on adaptive developmental outcomes, the 7Cs are proposed to serve as important psychosocial predictors of hopeful future expectations. Intrapersonal strengths such as competence, confidence, and creativity may promote hope by strengthening young people's beliefs that they can effectively pursue goals, overcome obstacles, and generate alternative pathways when challenges arise. Interpersonal and ecological strengths such as connection, caring, character, and contribution may likewise nurture hope by providing supportive relationships, moral direction, social purpose, and opportunities for meaningful engagement with others. The 7Cs may promote a more optimistic and agentic orientation toward the future, increasing young people's beliefs that desired goals can be attained despite uncertainty and adversity. From a developmental perspective, it is argued that youth strengths and talents, when cultivated within supportive environments, may promote a hopeful future orientation, even in adverse circumstances (Ismailov, 2026). Meta‐analytic evidence indicates that hope is strongly predicted by positive psychological traits and social support and negatively associated with depression, stress, and adverse experiences (Yarcheski & Mahon, 2016). These patterns position hope as a key developmental asset that may facilitate the links between PYD characteristics and life satisfaction.

The proposed ordering of the variables assumes that PYD strengths constitute relatively stable psychosocial resources that may shape how young people think about and approach their futures, whereas hope reflects a future‐oriented motivational process that subsequently contributes to evaluations of well‐being (Ismailov, 2026). Recent empirical work supports hope as a plausible mediating mechanism linking PYD dimensions to life satisfaction. Among Malaysian emerging adults, the 5Cs of PYD were positively associated with hope, which in turn mediated the relationships between specific PYD dimensions and psychological well‐being (Abdul‐Kadir & Mohd, 2021). Specifically, confidence and connection indirectly predicted well‐being through hope, suggesting that belief in one's abilities and supportive social relationships foster hopeful thinking, which subsequently enhances well‐being. Beyond the PYD literature, hope has been shown to mediate the associations of resilience, psychological vulnerability, meaning in life, purpose, with life satisfaction across diverse cultural contexts (Bronk et al. 2009; Murphy, 2023; Satici, 2016; Yalçın & Malkoç, 2015). By fostering agency and pathways thinking, hope may enable individuals to overcome challenges, sustain motivation, and experience progress toward valued goals (Bowers & Bowers, 2023). Despite these advances, few studies have explicitly examined hope as a mediating mechanism linking the broader 7Cs of PYD to life satisfaction. Addressing this gap, the present study proposes hope as a theoretically grounded and empirically supported mediator that explains how PYD characteristics are translated into greater satisfaction with life among young people.

## PYD IN EMERGING ADULTHOOD

Emerging adulthood, spanning the ages of 18 to 25 years, and extending to the late twenties, is a proposed time of life that can be characterized by heightened identity exploration, increased autonomy, delayed transitions into traditional adult roles, and a subjective sense of feeling “in‐between” adolescence and adulthood (Arnett, 2024). Although this phase is often marked by uncertainty regarding educational, occupational, and relational futures, it also presents unique opportunities for growth, self‐discovery, and positive adaptation (Nelson, 2021). Emerging adults are tasked with making consequential decisions about their careers, relationships, values, and life directions while navigating increasingly complex societal issues (Arnett, 2024). These demands make the examination of strength‐based attributes particularly relevant during this period. From a PYD perspective, thriving characteristics such as the 7Cs may serve as important psychosocial resources that may help young adults successfully negotiate challenges and capitalize on opportunities for growth as they transition toward established adulthood (Buenconsejo, Ferrer‐Wreder, et al. 2025).

Although PYD frameworks were originally developed to explain positive adaptation during adolescence, developmental scientists have argued that these strengths remain relevant beyond the teenage years because positive characteristics cultivated during adolescence often extend into subsequent stages of development (Arnett, 2024; Bornstein et al. 2026). However, the manifestations of PYD may evolve alongside changing developmental tasks and social roles. As emerging adults enter higher education or the workforce, for example, competence may become increasingly expressed through occupational skills, career readiness, and independent living capacities over academic performance alone (Filosa et al. 2022; Gomez‐Baya et al. 2025). Similarly, confidence may manifest in stronger vocational identities and greater self‐mastery, while connection expands beyond relationships with parents and teachers to include peers, romantic partners, mentors, and professional networks (Arnett, 2024; Nelson, 2021). Character may become increasingly grounded in internalized values and ethical commitments beyond external rules and regulations (Lapsley et al. 2023), whereas caring and contribution may be expressed through broader forms of civic engagement and social responsibility facilitated by advances in perspective‐taking and prosocial reasoning (Eisenberg et al. 2005). Moreover, continued brain maturation during emerging adulthood supports more sophisticated forms of creative thinking and adaptive problem‐solving that may enable young people to cope with the demands of adult life (King & Kitchener, 2016). Thus, while the developmental contexts in which strengths are enacted may change, PYD characteristics remain important assets that support healthy maturation and well‐being during emerging adulthood (Arnett, 2024; Bornstein et al. 2026).

Recent evidence supports the applicability of PYD frameworks among emerging adults across diverse cultural contexts. In Southeast Asia, one cross‐national study established the validity and measurement invariance of the 7Cs model across countries, gender, age, and educational groups, demonstrating its relevance for understanding thriving among emerging adults in the region (Buenconsejo, Ferrer‐Wreder, et al. 2026). Findings also showed that connection, character, caring, and contribution were positively associated with socially responsive behaviors during the COVID‐19 pandemic, whereas competence, confidence, and connection were linked to lower levels of anxiety. At the same time, higher levels of caring, contribution, and creativity were associated with greater anxiety, suggesting that certain strengths may confer both benefits and challenges based on contextual demands (Buenconsejo, Ferrer‐Wreder, et al. 2026). Complementing these findings, a longitudinal study spanning early adolescence to emerging adulthood across nine countries found that PYD trajectories varied according to national economic contexts, while supportive parenting practices, particularly parental warmth, predicted higher levels of PYD from adolescence into emerging adulthood (Bornstein et al. 2026). Collectively, these findings indicate that PYD remains a meaningful framework for understanding thriving during emerging adulthood while also highlighting the importance of considering developmental, cultural, and structural contexts when examining how strengths are expressed and translated into adaptive outcomes among young people.

## EXTENDING PYD IN THE MAJORITY WORLD

PYD scholarship has been largely developed and empirically validated among youth samples from Minority World contexts, particularly in North America and Europe (Koller & Verma, 2017). This raises important concerns regarding the cross‐cultural generalizability of PYD models and measures (Wiium & Dimitrova, 2019). Developmental scientists caution that PYD frameworks derived primarily from Western contexts may insufficiently capture the cultural, social, and structural conditions shaping youth development in Majority World countries, where norms, values, and developmental affordances often differ markedly (Wiium & Dimitrova, 2019). Extending PYD to these contexts is therefore both a theoretical and practical imperative, as youth in the Majority World frequently face distinct challenges, including socio‐economic inequality as well as political and environmental instability (ASEAN, 2023; Buenconsejo & Datu, 2023a). By examining PYD within culturally diverse and underrepresented settings, researchers can better understand how promotive factors embedded in family, school, and community systems can support youth thriving and inform culturally responsive policies and interventions.

Recent investigations have begun to examine the applicability of the 7Cs model of PYD in Majority World contexts, with promising but methodologically varied findings. Empirical investigations conducted among emerging adults in Malaysia (Abdul Kadir et al. 2021), Colombia and Peru (Manrique‐Millones et al. 2021), as well as India, Indonesia, and Pakistan (Dimitrova et al. 2021) reported acceptable model fit for the 7Cs framework. However, these studies operationalized PYD as a unidimensional construct using aggregate scores of the 7Cs, thereby limiting insights into the distinct roles of individual PYD dimensions. Addressing this limitation, a more recent large‐scale study among Southeast Asian emerging adults rigorously examined the factorial validity and measurement invariance of the 7Cs using competing measurement models (Buenconsejo, Ferrer‐Wreder, Dimitrova, et al. 2026). The findings also revealed meaningful cultural variations in the expression of specific strengths. Emerging adults from Malaysia and Singapore, which tend to be more achievement‐oriented contexts, reported relatively higher levels of competence, confidence, and creativity, whereas those from Indonesia, the Philippines, and Thailand noted higher levels of connection, character, caring, and contribution, possibly explained by the heightened familial and community ties in these countries (Buenconsejo, Ferrer‐Wreder, et al. 2026; Yeung et al. 2018). These patterns suggest that PYD does not manifest uniformly across contexts but may reflect the developmental priorities, educational opportunities, cultural values, and social expectations that characterize different societies.

Beyond measurement considerations, recent cross‐cultural research suggests that PYD dimensions may not function equivalently across sociocultural contexts (Buenconsejo, Datu, & Liu 2025). Recent theorizing distinguishes between *intrapersonal‐immediate indicators* (e.g., competence, confidence, and creativity), which emphasize self‐efficacy and personal achievement, as well as *interpersonal‐proximal* and *ecological‐distal indicator*s (e.g., connection, character, caring, and contribution), which reflect relational and moral orientations (Buenconsejo & Datu, 2023a). Empirical evidence supports this distinction, showing that efficacy‐related Cs tend to be more salient in contexts with relatively stronger individualistic orientations such as the United States (Yang & McGinley, 2021), whereas socio‐emotional Cs may play a more prominent role in societies with relatively stronger collectivistic orientations, such as Hong Kong (Wong et al. 2021), Taiwan (Yang & McGinley, 2021), and the Philippines (Buenconsejo & Datu, 2023b). Nonetheless, these distinctions should not be interpreted as rigid cultural binaries as evidence suggests that both efficacy‐related and socio‐emotional strengths may contribute to well‐being, albeit through different pathways and with varying levels of salience across contexts (Buenconsejo, Ferrer‐Wreder, et al. 2025). Recent findings also reveal nuanced and sometimes counterintuitive associations, with high competence linked to elevated depressive symptoms among Filipino adolescents (Buenconsejo & Datu, 2024), and confidence associated with lower empathic and altruistic tendencies among Southeast Asian youth (Buenconsejo et al. 2024). These patterns underscore the importance of examining PYD within understudied regions such as Southeast Asia, where cultural values and structural conditions may shape the function of specific PYD attributes.

## THE SOUTHEAST ASIAN CONTEXT

Southeast Asia represents a highly salient yet underexamined context for advancing PYD scholarship, given its demographic significance and profound sociocultural diversity. The region is home to more than 224 million youth aged 15 to 34, accounting for approximately 12% of the global youth population (ASEAN, 2023). The region comprises 11 countries that form the Association of Southeast Asian Nations (ASEAN), a regional bloc committed to economic growth, social progress, and cultural development (ASEAN, 2023). Although Southeast Asian societies share certain historical and philosophical influences, including Confucian and East Asian values, precolonial belief systems, and the legacies of colonial rule, these commonalities co‐exist with substantial diversity in language, religion, political systems, and socio‐economic conditions (Rattanasevee, 2023; Yeung et al. 2018). As such, specific national contexts often outweigh any singular regional identity, underscoring the importance of examining youth development through frameworks that are sensitive to both shared and context‐specific developmental processes.

The present study focuses on six Southeast Asian countries—Indonesia, Malaysia, the Philippines, Thailand, Singapore, and Vietnam—that collectively illustrate the region's breadth of cultural, political, and developmental conditions (Rattanasevee, 2023). Indonesia (67.9 million youth), the world's largest archipelago, is marked by deep ethnic diversity and a political system shaped by historical colonization and patronage networks. Malaysia's (11.6 million youth) multicultural society reflects its colonial past and a governance structure influenced by informal and elite‐driven decision‐making. The Philippines (33.6 million youth) exhibits a distinctive blend of Western and Asian influences, alongside persistent governance and regional integration challenges. Singapore (1.8 million youth), a highly developed city‐state, adopts a pragmatic and globally oriented stance while balancing its ASEAN commitments. Thailand's (19.4 million youth) sociopolitical landscape is shaped by a long‐standing monarchy and recurrent political instability, whereas Vietnam (24.5 million youth), a socialist state, has demonstrated strong citizen engagement with regional identity and integration efforts (ASEAN, 2023; Rattanasevee, 2023).

Emerging adults from these Southeast Asian countries encounter distinct combinations of opportunities and challenges that may shape the development and expression of PYD strengths (ASEAN, 2023). In Indonesia, expanding educational access and digital engagement provide opportunities for competence and contribution, although unemployment and inequalities in access to developmental resources remain concerns. Malaysian youth benefit from strong digital infrastructure and inclusion initiatives but continue to face challenges related to mental health and labor market transitions. In the Philippines, growing civic engagement and volunteerism create opportunities for connection, caring, and contribution, while unemployment, social inequalities, and limited mental health support may hinder thriving. Thailand has made gains in education, inclusion, and civic participation, yet rising mental health concerns and employment challenges continue to affect youth well‐being. Singapore offers extensive educational, occupational, and technological opportunities that may foster competence, confidence, and creativity, although concerns related to labor force participation and cultural belonging persist. Vietnam has experienced improvements in education, civic engagement, and social inclusion while continuing to address mental health and equity‐related challenges (ASEAN, 2023). Collectively, these diverse developmental conditions illustrate why PYD strengths may be expressed differently across Southeast Asian contexts and underscore the importance of examining youth thriving within specific sociocultural and structural environments.

From a developmental perspective, PYD may be particularly relevant for Southeast Asian emerging adults because many are negotiating educational, occupational, and social transitions while simultaneously confronting structural challenges that can hinder successful adulthood transitions. Across the region, issues such as youth unemployment, educational inequalities, mental health concerns, environmental vulnerabilities, and rapidly changing labor market demands continue to shape young people's developmental experiences (ASEAN, 2023; Rattanasevee, 2023). At the same time, greater access to higher education, digital technologies, and civic engagement opportunities provides important pathways for growth and contribution. Within these contexts, the 7Cs may function as key developmental assets that can help emerging adults adapt to uncertainty, pursue meaningful goals, maintain supportive relationships, exercise moral responsibility, and contribute positively to their communities (Buenconsejo, Ferrer‐Wreder, Dimitrova, et al. 2026; Dimitrova et al. 2021). Yet despite growing interest in youth well‐being in Southeast Asia, research has largely focused on mental health and other applied outcomes, with comparatively limited attention to integrative developmental frameworks (Bernardo et al. 2022). Consequently, further examining the 7Cs in Southeast Asia can advance a more culturally relevant understanding of youth thriving while identifying both shared and context‐specific developmental processes across the region.

## THE PRESENT STUDY

Despite substantial advances in PYD scholarship, several important gaps remain, particularly in Majority World contexts. First, much of the extant literature has relied on Western youth samples and has often operationalized PYD as a global construct or examined only selected Cs, thereby limiting understanding of how specific PYD dimensions uniquely relate to well‐being outcomes. Second, although recent evidence suggests that PYD attributes may function differently across sociocultural contexts, few studies have systematically and simultaneously examined the mechanisms linking PYD characteristics to well‐being among culturally diverse youth samples in Southeast Asia. Third, while hope has been identified as a central developmental asset and a plausible psychological mechanism through which psychosocial resources foster well‐being, its mediating role between the broader 7Cs of PYD and life satisfaction remains underexplored, particularly using multi‐country analytic approaches. The present study addresses these gaps by examining the direct and indirect associations between the 7Cs of PYD, hope, and life satisfaction among emerging adults from six Southeast Asian countries—Indonesia, Malaysia, the Philippines, Thailand, Singapore, and Vietnam—thereby providing a more comprehensive and context‐sensitive account of youth thriving in the region.

Guided by the 7Cs model of PYD (Dimitrova et al. 2021) and the integrative paradigm of PYD (Buenconsejo & Datu, 2022), this study sought to address three major objectives: (1) to examine the associations between the 7Cs of PYD with hope and life satisfaction while controlling for age, gender, and educational attainment; (2) to test the indirect pathways linking the 7Cs of PYD to life satisfaction through hope, controlling for the same socio‐demographic covariates; and (3) to investigate country‐specific differences in the indirect relations between the 7Cs and life satisfaction via hope across the six Southeast Asian samples. Socio‐demographic covariates were included to establish the unique predictive validity of PYD dimensions, given consistent evidence that older, female, and more educated youth tend to report higher levels of PYD (Buenconsejo, Ferrer‐Wreder, et al. 2025; Geldhof et al. 2014).

As shown in Figure 1, the hypothesized model proposes that the 7Cs of PYD will positively foster hope, which in turn is expected to promote greater life satisfaction through sustained motivation and goal‐directed engagement. It is hypothesized that intrapersonal (competence, confidence, and creativity), interpersonal (connection and caring), and ecological (character and contribution) PYD dimensions will show positive associations with life satisfaction above and beyond socio‐demographic covariates, with hope mediating these associations. Consistent with prior findings and the collectivistic orientations prevalent in many Southeast Asian societies, stronger indirect paths are anticipated for interpersonal PYD indicators, particularly connection (Buenconsejo et al. 2022; Holsen et al. 2017; Wong et al. 2021), alongside country‐specific pathways reflecting contextual variations in youth development processes in the Southeast Asian region (Rattanasevee, 2023; Yeung et al. 2018).

**FIGURE 1:**
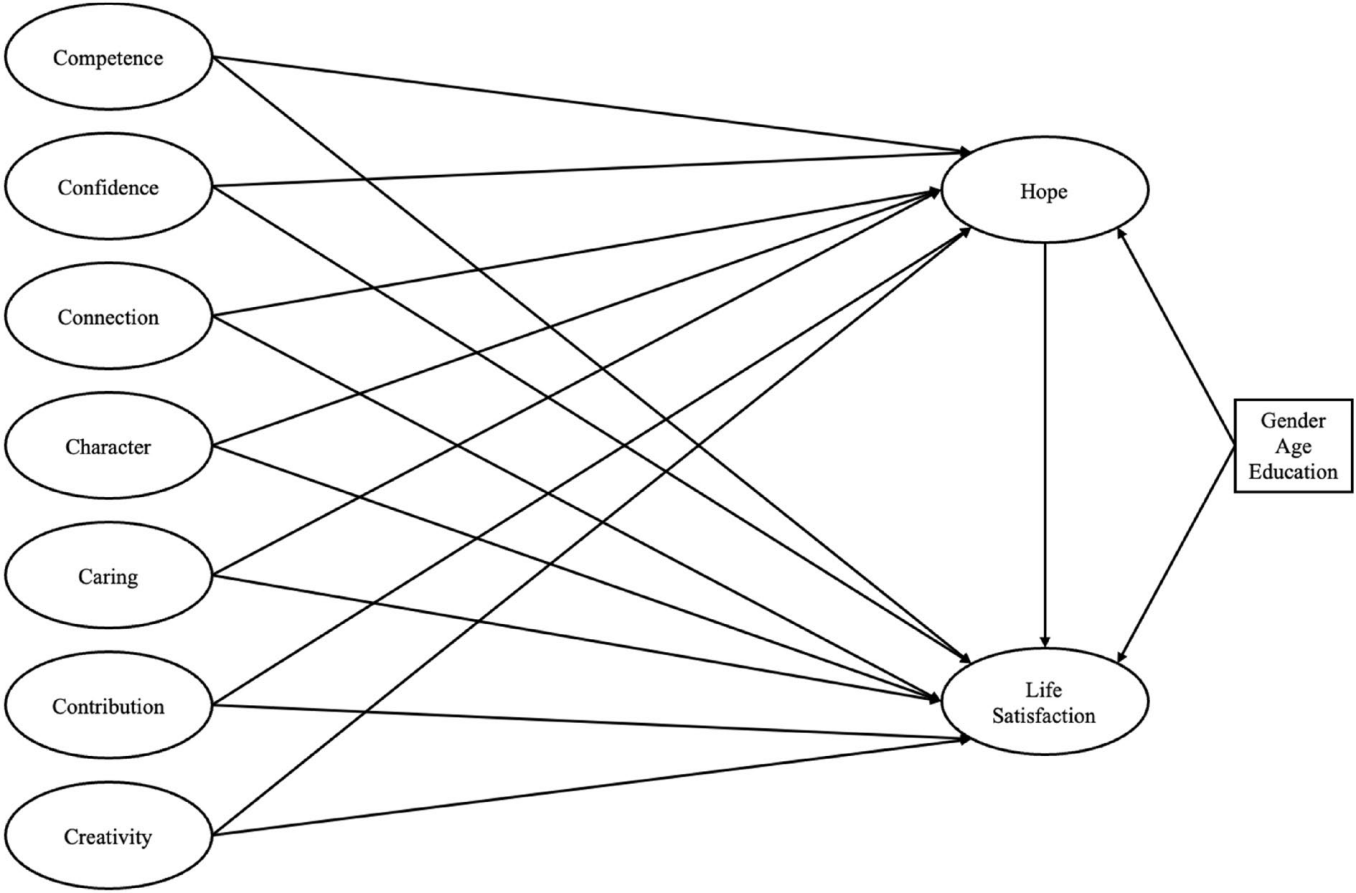
Conceptual Model of the Indirect Associations Between the 7Cs of Positive Youth Development and Life Satisfaction via Hope, Controlling for Gender, Age, and Education. Manifest variables (i.e., items) and error terms were intentionally omitted for the purpose of parsimony.

## METHODS

### Participants

Data were obtained from 1967 emerging adults aged 18 to 25 (*M*_*age*_ = 20.99; *SD*_*age*_ = 1.79) from Indonesia (*n* = 227), Malaysia (*n* = 280), the Philippines (*n* = 479), Singapore (*n* = 247), Thailand (*n* = 486), and Vietnam (*n* = 248). The majority of the participants were females (74%) who had completed an undergraduate degree (65%). About a third or 33% completed a secondary or high school diploma, while about 1% had a post‐graduate degree. Among the respondents, 64% were full‐time students, while 4% were full‐time employees. About 9% of the respondents reported studying and working at the same time, while 1% noted that they were neither studying nor working. Table 1 presents the socio‐demographic characteristics of the participants from each country site.

**TABLE 1:** Socio‐demographic profiles of the youth samples from the six Southeast Asian countries.

Youth sample	*n*	Mean age (standard deviation)	Gender		Highest educational attainment		
			Males (%)	Females (%)	High school diploma (%)	University degree (%)	Post‐graduate degree (%)
Overall sample	1967	20.99 (1.79)	25.93	74.07	34.16	64.92	0.92
Indonesia	227	20.41 (1.62)	23.79	76.21	73.57	25.11	1.32
Malaysia	280	22.55 (1.66)	19.64	80.36	7.85	90.36	1.79
Philippines	479	19.92 (1.24)	35.28	64.72	39.87	59.92	0.21
Singapore	247	22.77 (1.31)	25.10	74.90	37.25	61.94	0.81
Thailand	486	20.66 (1.51)	19.96	80.04	32.72	67.28	0.00
Vietnam	248	20.68 (1.41)	29.44	70.56	16.53	80.65	2.82

Almost all of the participants were natural born citizens in the six specified countries. As for their ethnicity, the majority of the participants from Malaysia, Thailand, the Philippines, and Vietnam identified themselves as Malay (99%), Thai (99%), Filipino (97%), and Kinh/Viet (94%), respectively. For Indonesia, most of the participants identified either as Chinese/Tionghoa (43%) or Javanese/Jawa (23%), while the rest were a mixture of Batak, Manado, Minahasa, and Suku. Majority of the participants from Singapore identified as Chinese (89%), while the rest identified as Indian (6%), Malay (1%), Caucasian (1%), and Mixed (3%).

### Measures

#### Positive youth development

The 7Cs of PYD were measured using the 32‐item scale proposed by Dimitrova et al. (2021) and validated with Southeast Asian emerging adults (Buenconsejo, Ferrer‐Wreder, Dimitrova, et al. 2026). The scale was adapted from the very short measure of the 5Cs (Geldhof et al. 2014), contribution scale (Lerner et al. 2005), and creativity scale (Reisman et al. 2016). Competence included 3 items on academic, social, and skill competence (e.g., “I do very well in my work at school, university, or workplace”). Confidence included 3 items on self‐worth, positive identity, and physical appearance (e.g., “All in all, I am glad I am me”). Connection included 4 items pertaining to relationships in their families, neighborhoods, schools, and peers (e.g., “I am a useful and important member of my family”). Character included 4 items on social conscience, diversity valuation, conduct behavior, and personal values (e.g., “I accept responsibility for my actions when I make a mistake or get into trouble”). Caring included items on experiencing sympathy and empathy for others (e.g., “When I see someone being taken advantage of, I want to help them”). Creativity included 9 items related to the generation of new ideas and novel solutions to address specific challenges in one's environment (e.g., “I can come up with novel uses for things”). Items for competence, confidence, connection, character, caring, and creativity were rated using a 5‐point scale (1 = *strongly disagree* to 5 = *strongly agree*). Contribution included 6 items measuring the weekly frequency of helping other people and providing community services (e.g., volunteering, mentoring peers, or participating in service‐oriented committees) rated on a 5‐point scale (1 = *0 h* to 5 = *6 or more hours*). The reliability coefficients (Cronbach's alpha and McDonald's omega) of all constructs among the entire sample are presented in Table 2. The 7Cs of PYD exhibited adequate reliability estimates ranging between .71 (connection) to .95 (creativity). Tables A1 to A.6 of the supporting information present the reliability estimates of all constructs per country site. The country‐specific reliability estimates ranged between .70 (connection in Singapore) to .96 (creativity in Malaysia, Singapore, and Thailand).

**TABLE 2:** Descriptive statistics and reliability coefficients of the 7Cs of positive youth development, hope, and life satisfaction among youth from the six Southeast Asian countries.

Variable	*n*	Minimum	Minimum	Mean	Standard deviation	Skewness	Kurtosis	Cronbach's ⍺	McDonald's ⍵
Competence	1967	1.00	5.00	3.17	0.75	0.13	−0.04	0.78	0.80
Confidence	1967	1.00	5.00	3.51	0.87	−0.39	−0.09	0.82	0.82
Character	1967	1.00	5.00	3.87	0.61	−0.42	0.64	0.78	0.79
Caring	1967	1.00	5.00	4.10	0.69	−0.61	0.57	0.81	0.82
Connection	1967	1.00	5.00	3.66	0.71	−0.16	−0.14	0.71	0.76
Contribution	1967	1.00	5.00	2.68	0.92	0.33	−0.40	0.78	0.85
Creativity	1967	1.00	5.00	3.50	0.71	−0.14	0.16	0.93	0.95
Hope	1967	1.00	5.00	3.76	0.66	−0.28	0.27	0.91	0.93
Life satisfaction	1967	1.00	7.00	4.35	1.36	−0.30	−0.38	0.87	0.88

#### Hope

The 12‐item Herth Hope Index (HHI; Herth, 1992) was used to measure hope or the belief in a positive future, characterized by temporality and future orientation, positive readiness and expectancy, and interconnectedness with others. Sample items include “I can see possibilities in the midst of difficulties” and “I believe that each day has potential.” Items were rated using a 5‐point scale ranging between 1 = *strongly disagree* to 5 = *strongly agree*. The HHI exhibited .91 Cronbach's alpha and .93 McDonald's omega. The country‐specific reliability estimates of hope ranged between .88 (Indonesia) to .95 (Malaysia).

#### Life satisfaction

The 3‐item Satisfaction with Life Scale (SWLS; Kjell & Diener, 2021) was used to measure life satisfaction or the global assessment of one's quality of life, encompassing feelings of happiness, contentment, and fulfillment. A sample item includes “The conditions of my life are excellent.” Items were rated using a 7‐point scale ranging between 1 = *strongly disagree* to 7 = *strongly agree*. The SWLS exhibited .87 Cronbach's alpha and .88 McDonald's omega. The country‐specific reliability estimates of life satisfaction ranged between .84 (Indonesia) and .89 (Malaysia, Thailand, and Vietnam).

#### Socio‐demographic covariates

Aside from the PYD, hope, and life satisfaction, we also asked for the participants' gender (1 = male and 2 = female), age (18 to 25), and highest educational attainment (1 = high school diploma, 2 = university degree, and 3 = post‐graduate degree). Gender was treated as a binary covariate, while age and educational attainment were treated as continuous and ordinal covariates, respectively. These covariates were included in the structural models to estimate the unique associations of the 7Cs with hope and life satisfaction after accounting for socio‐demographic characteristics.

#### Procedures

This study was part of a larger project called “International and Multidimensional Perspectives on the Impact of COVID‐19” (IMPACT‐C19), which was led by the Research Initiatives Work Group of the American Psychological Association Interdivisional Task Force on the Pandemic. The project examined the impact, perceptions, and experiences of COVID‐19 among young people and adults from an international perspective with multidisciplinary expertise involving over 150 members and data from over 80 countries. This international project followed a committee approach in adapting the survey instruments for each country, which included translating and adapting the items in the local language and context (van de Vijver et al. 2019). For Indonesia, Thailand, and Vietnam, the measures were translated in Bahasa Indonesia, Thai, and Viet, which were validated by at least two experts in each site. Translation experts possessed at least a master's degree in psychology, education, or social sciences and were native speakers of the language in their respective country. The instruments were administered in English in Malaysia, the Philippines, and Singapore because this is the main language of instruction in these countries.

Ethical clearance was obtained from the authors' respective universities in each site. Participants were recruited via convenience sampling through announcements posted on social media platforms and online groups created during the pandemic. Participants were informed about the purposes of the study and were informed that participation in the study was completely voluntary. The online survey was administered via Qualtrics between February 2021 and June 2022. Note that data for this study are cross‐sectional and are not meant to be nationally representative of all youth within the specified countries.

#### Data analyses

Descriptive statistics (i.e., mean, standard deviation, skewness, and kurtoses), reliability estimates (i.e., Cronbach's alpha and McDonald's omega), and Pearson's r correlations were calculated using the 30th version of the Statistical Package for the Social Sciences (SPSS). Missing responses ranged between 2.5% (competence) to 4.4% (life satisfaction). Little's MCAR test showed that missing responses were missing completely at random, *χ* ^*2*^ (1202) = 1283.76, *p* = .350. Missing responses were estimated separately per country at the item level using the multivariate imputation by chained equations (MICE) package in R (van Buuren & Groothuis‐Oudshoorn, 2011).

Prior to conducting the main analyses, multi‐group confirmatory factor analyses (MG‐CFAs) were performed to examine if the measures exhibited configural (equivalence in the factor structure), metric (equivalence in the factor loadings), and scalar (equivalence in item intercepts) invariance across the six Southeast Asian countries. Measurement invariance was evaluated using changes in approximate fit indices and was supported when changes in CFI, RMSEA, and SRMR remained within the recommended cut‐offs of .010, .015, and .010, respectively (Chen, 2007). Note that MG‐CFAs were conducted for the 7Cs of PYD and hope, but not for life satisfaction, because the SWLS comprises only three items and thus yields a just‐identified one‐factor measurement model with global fit indices that are not informative. Instead, we inspected the standardized factor loadings of the SWLS items across the six countries, ensuring that all were substantial (≥ .40) and in the expected positive direction.

The two‐step approach to structural equation modeling (SEM) (Anderson & Gerbing, 1988) was used to examine the direct and indirect paths of the 7Cs of PYD to life satisfaction via hope, while controlling for participants' gender, age, and education. In Step 1, a measurement model with only the latent constructs (i.e., 7Cs of PYD, hope, and life satisfaction) and their observed indicators (i.e., items) were estimated using confirmatory factor analysis (CFA). No regression paths among the latent variables and no covariates were included at this stage. After securing a well‐fitting measurement model, SEM was specified in Step 2. Here, regression paths between the 7Cs of PYD, hope, and life satisfaction were estimated along with gender, age, and education as covariates. The following cut‐off values were used in evaluating the fit of the models: (a) Comparative Fit Index (CFI) and Tucker–Lewis Index (TLI) greater than .90 and (b) Standardized Mean Square Residual (SRMR) and Root Mean Square Error of Approximation (RMSEA) less than .08 (Hair et al. 2018).

The indirect associations between the 7Cs of PYD and life satisfaction via hope were examined using 95% confidence intervals from the delta method in lavaan, which estimates the products of structural paths (e.g., competence → hope [path a] × hope → life satisfaction [path b]). In line with conventional mediation practice, an indirect effect was considered statistically significant when its 95% confidence interval did not include zero (Preacher & Hayes, 2008). Aside from examining the overall SEM (i.e., using the entire Southeast Asian dataset), we also estimated country‐specific models (i.e., separate SEMs for Indonesia, Malaysia, Singapore, Thailand, Philippines, and Vietnam) to examine whether the structural paths differed meaningfully across countries.

All parameter estimates were obtained using Weighted Least Squares Mean and Variance adjustment (WLSMV), a limited‐information diagonally weighted least squares estimator that provides robust standard errors and a mean–variance‐adjusted chi‐square test statistic for models with ordinal data and mixed response options (Li, 2016; Muthén et al. 1997). In lavaan, the item‐level indicators for the 7Cs of PYD, hope, and life satisfaction were declared as “ordered” and were therefore treated as ordinal variables with thresholds and polychoric/polyserial correlations, whereas gender, age, and education were included as observed exogenous covariates using their coded values. Gender was specified as a binary covariate, age as a continuous covariate, and education as an ordinal covariate. This implementation follows current recommendations for CFA and SEM with Likert‐type items, where WLSMV has been shown to yield more accurate parameter estimates and fit indices compared to maximum likelihood estimation (Brauer et al. 2023; Rosseel, 2014). Both the MG‐CFAs and SEM were performed using the lavaan package in R (Rosseel, 2012).

## RESULTS

### Descriptive statistics and bivariate correlations

The descriptive statistics and reliability coefficients of the 7Cs of PYD, hope, and life satisfaction among the entire sample are presented in Table 2. There were no indications of severe violations of normality among the constructs, as indicated by acceptable skewness (−2 to +2) and kurtosis (−7 to +7) values (Hair et al. 2018). Tables A1 to A.6 of the supporting information present the descriptive statistics and reliability coefficients of the variables per country site.

Table 3 shows the bivariate correlations of the variables using the entire sample. The 7Cs of PYD, hope, and life satisfaction exhibited significant positive correlations with each other ranging from .12 (contribution and life satisfaction) to .61 (character and caring). Competence, confidence, and connection were moderately correlated with each other (.45 to .54), whereas caring showed the strongest association with character (.61). Contribution was most strongly associated with connection (.32), while creativity exhibited the highest correlations with connection (.39) and character (.38). Hope demonstrated moderate to strong correlations with all 7Cs ranging between .25 (contribution) to .60 (connection). Life satisfaction was moderately related to competence, confidence, connection, character, and hope (.30 to .53), but showed comparatively smaller associations with caring, contribution, and creativity (.12 to .25). Tables B.1 to B.6 of the supporting information present the country‐specific bivariate correlations.

**TABLE 3:** Bivariate correlations of the 7Cs of positive youth development, hope, and life satisfaction among youth from the six Southeast Asian countries (*n* = 1967).

	Competence	Confidence	Character	Caring	Connection	Contribution	Creativity	Hope	Life satisfaction
Competence	‐								
Confidence	.449**	‐							
Character	.347**	.426**	‐						
Caring	.210**	.209**	.608**	‐					
Connection	.526**	.535**	.472**	.412**	‐				
Contribution	.228**	.181**	.186**	.178**	.319**	‐			
Creativity	.354**	.304**	.380**	.292**	.386**	.304**	‐		
Hope	.434**	.586**	.484**	.366**	.603**	.246**	.459**	‐	
Life Satisfaction	.355**	.495**	.298**	.189**	.458**	.120**	.250**	.533**	‐

*Note*: **Correlation is significant at the 0.01 level (2‐tailed).

### Multi‐group confirmatory factor analyses

The results of the MG‐CFAs for the seven‐factor model of the 7Cs of PYD and the one‐factor model of hope across the six Southeast Asian countries are presented in Tables C.1 and C.2 of the supporting information. For the 7Cs of PYD, the configural model showed good fit (CFI = 0.975, RMSEA = 0.042, SRMR = 0.063), indicating that the seven‐factor structure was tenable across countries. Adding equality constraints on factor loadings (metric model) and thresholds (scalar model) produced changes in CFI, RMSEA, and SRMR that remained within the recommended invariance cut‐offs, supporting metric and scalar invariance of the 7Cs of PYD across the six countries. Similarly, for hope, the configural model also showed good fit (CFI = 0.987, RMSEA = 0.048, SRMR = 0.053), and changes in the fit indices for the metric and scalar models remained within the recommended cut‐offs, supporting measurement invariance across the six countries. Given that the SWLS has only three items, we did not implement a full MG‐CFA for life satisfaction. Instead, we examined the factor loadings of the three SWLS items in the overall and country‐specific datasets, which were consistently substantial (≥ .40) and positively correlated with the latent construct. Detailed standardized factor loadings for the 7Cs of PYD, hope, and life satisfaction in the overall and country‐specific samples are reported in Supplementary Materials D, E, and F.

### Overall structural equation modeling

The results of the two‐step approach to SEM are presented in Table 4. Specifically, the measurement model containing the latent and manifest variables (Step 1) and the SEM indicating the regression paths between the 7Cs of PYD, hope, and life satisfaction (Step 2) both showed adequate fit with the overall dataset. Tables G.1 and H.1 of the supporting information present the standardized factor loadings of the measurement model in Step 1 and the SEM in Step 2 among the entire youth sample, respectively. Figure 2 shows the significant regression paths among the variables.^1^

**TABLE 4:** Results of the overall and country‐specific confirmatory factor analyses (Step 1) and structural equation m (Step 2) on the 7Cs of positive youth development, hope, and life satisfaction, controlling for gender, age, and education.

Measurement model	χ^*2*^	*df*	*p*‐value	CFI	TLI	RMSEA	90% CI	SRMR
Step 1: confirmatory factor analyses								
Overall sample (*n* = 1967)	8730.472	998	< .001	.985	.983	.063	.062, .064	.054
Indonesia (*n* = 227)	1986.428	998	< .001	.956	.952	.066	.062, .070	.058
Malaysia (*n* = 280)	1843.012	998	< .001	.964	.961	.055	.051, .059	.064
Philippines (*n* = 479)	3056.397	998	< .001	.975	.973	.066	.063, .068	.072
Singapore (*n* = 247)	1730.883	998	< .001	.986	.985	.055	.050, .059	.064
Thailand (*n* = 486)	2917.972	998	< .001	.983	.982	.063	.060, .066	.061
Vietnam (*n* = 248)	2121.943	998	< .001	.981	.979	.068	.064, .072	.071
Step 2: structural equation modeling								
Overall sample (*n* = 1967)	11810.833	1133	< .001	.989	.990	.069	.068, .070	.059
Indonesia (*n* = 227)	2111.019	1133	< .001	.956	.958	.062	.058, .066	.059
Malaysia (*n* = 280)	2014.535	1133	< .001	.967	.968	.053	.049, .057	.064
Philippines (*n* = 479)	3080.316	1133	< .001	.973	.974	.060	.057, .063	.072
Singapore (*n* = 247)	1926.745	1133	< .001	.961	.963	.053	.049, .057	.075
Thailand (*n* = 486)	2983.678	1133	< .001	.949	.951	.058	.056, .061	.070
Vietnam (*n* = 248)	1565.243	1133	< .001	.960	.956	.052	.045, .058	.070

*Note*: χ^*2*^ = chi‐square test statistic.

Abbreviations: *df* = degrees of freedom; CFI = Comparative Fit Index; TLI = Tucker–Lewis Index; RMSEA = Root Mean Square Error of Approximation; 90% CI = 90% Confidence Intervals of the Root Mean Square Error of Approximation; SRMR = Standardized Root Mean Square Residual.

**FIGURE 2:**
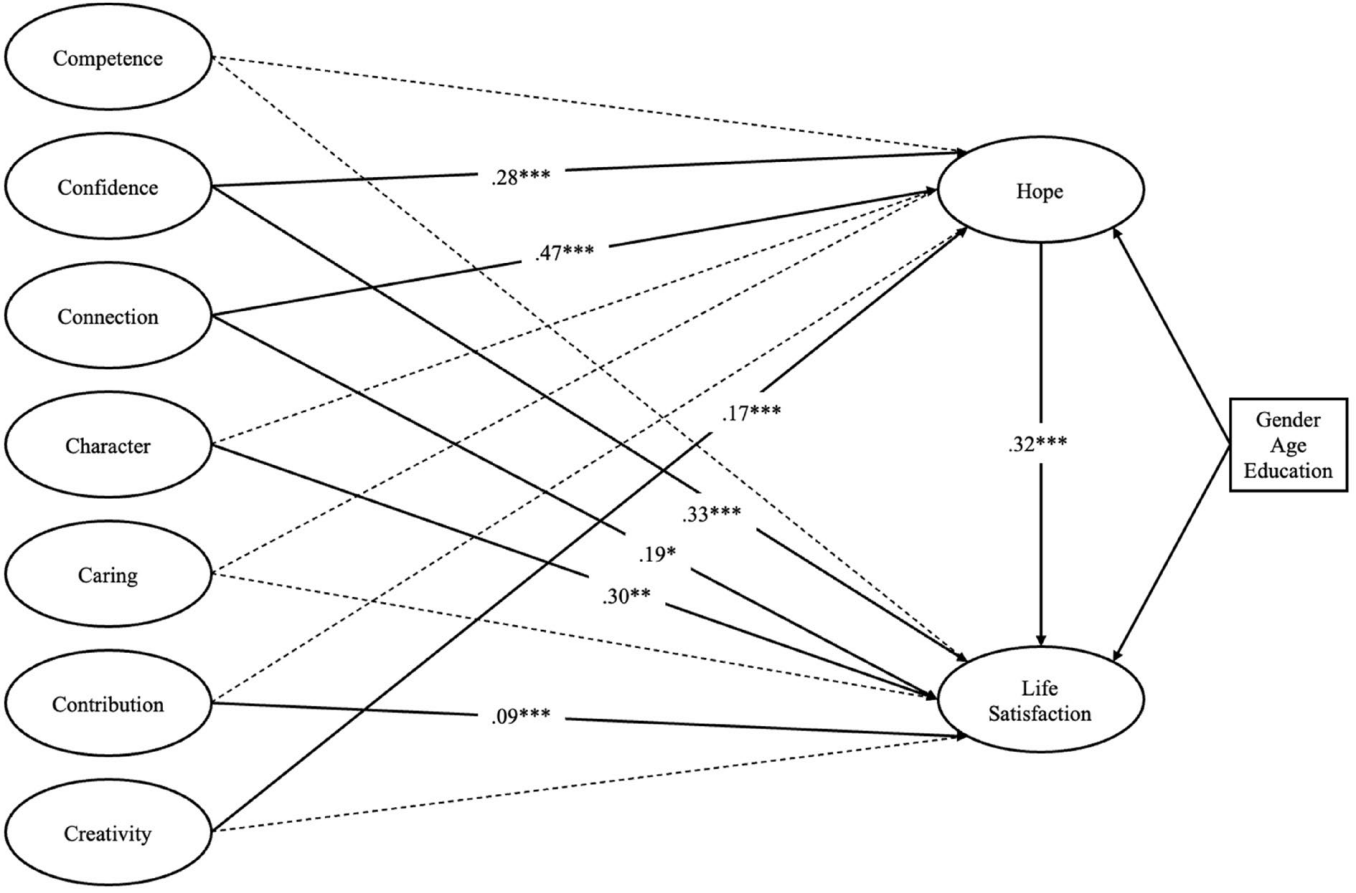
Results of the Overall Structural Equation Model Between the 7Cs of Positive Youth Development and Life Satisfaction Via Hope, Controlling for Gender, Age, and Education Among Youth from the Six Southeast Asian Countries (*n* = 1967). Significant standardized paths are shown in solid lines. Nonsignificant paths are shown in broken lines. Manifest variables (i.e., items) and error terms were intentionally omitted for the purpose of parsimony. ***p < .001; **p < .01; *p < .05.

Controlling for the influence of the participants' gender, age, and education, the PYD characteristics of confidence, connection, and creativity showed significant positive associations with hope, whereas competence, character, caring, and contribution were nonsignificant. Confidence, connection, character, and contribution were significantly and positively associated with life satisfaction, whereas competence, caring, and creativity were not. Hope was positively associated with life satisfaction. As for the socio‐demographic covariates, more educated youth reported higher levels of hope (β = .06, *p* < .05) and life satisfaction (β = .08, *p* < .001), whereas older participants were also more likely to be satisfied with life (β = .07, *p* < .01). Gender was not significantly associated with either outcome. Tables I.1 to I.7 of the supporting information present the complete regression paths of the SEM.

The analysis of indirect paths highlighted hope as a key psychological mechanism linking several PYD strengths to life satisfaction. The results of the mediation analyses for the overall and country‐specific youth samples are presented in Table 5. Controlling for the socio‐demographic covariates, significant positive indirect paths via hope were observed for confidence (β = .09, *p* < .001, 95% CI [.08, .17]), connection (β = .15, *p* < .001, 95% CI [.13, .29]), and creativity (β = .05, *p* < .001, 95% CI [.05, .10]), but not for competence, character, caring, and contribution. Confidence and connection each showed significant direct associations with life satisfaction alongside significant indirect associations through hope. In contrast, creativity showed a significant indirect association with life satisfaction through hope, whereas its direct association with life satisfaction was not significant. The overall model explained 48% of the variance of life satisfaction.

**TABLE 5:** Indirect paths of the 7Cs of positive youth development on life satisfaction via hope, controlling for gender, age, and education across youth from the six Southeast Asian countries.

PYD dimension	Overall (*n* = 1967)	Indonesia (*n* = 227)	Malaysia (*n* = 280)	Philippines (*n* = 479)	Singapore (*n* = 247)	Thailand (*n* = 486)	Vietnam (*n* = 248)
Competence	−.04 (−.10, .00)	.10 (−.14, .44)	−.32 (−.54, .43)	−.04 (−.16, .04)	.01 (−.04, .07)	−.05 (−.23, .09)	−.23 (−1.31, .71)
Confidence	.09*** (.08, .17)	.18* (.18, .82)	.10** (.08, .15)	.11*** (.07, .25)	.06* (.06, .16)	.01 (−.09, .12)	.31* (.26, .36)
Character	.04 (−.01, .13)	.11 (−.29, .63)	.13 (−.25, .70)	.08** (.04, .11)	.05 (−.08, .23)	.02 (−.15, .20)	−.52 (−3.25, 1.87)
Caring	.01 (−.05, .06)	−.08 (−.46, .21)	−.02 (−.58, .51)	.05 (−.02, .15)	−.01 (−.09, .05)	−.04 (−.21, .10)	.25 (−1.44, 2.09)
Connection	.15*** (.13, .29)	.16* (.11, .67)	.08** (.03, .09)	.09* (.02, .23)	.06* (.04, .20)	.24** (.13, .45)	.29* (.11, .59)
Contribution	−.01 (−.03, .01)	−.02 (−.09, .04)	−.02 (−.60, .52)	.01 (−.03, .05)	−.01 (−.05, .02)	−.01 (−.06, .03)	−.05 (−.34, .22)
Creativity	.05*** (.05, .10)	.08* (.02, .37)	.13* (.12, .14)	.05** (.02, .11)	.01 (−.02, .04)	.06*** (.04, .15)	.25* (.12, .60)

*Note*: Standardized significant indirect paths are highlighted in bold. The 95% delta‐method confidence intervals of the indirect paths are presented inside the parentheses. *** *p* < .001; ** *p* < .01; * *p* < .05.

### Country‐specific structural equation models

As shown in Table 4, country‐specific structural equation models indicated that the hypothesized relations among the 7Cs of PYD, hope, and life satisfaction provided acceptable fit in Indonesian, Malaysian, Filipino, Singaporean, Thai, and Vietnamese samples. These results suggest that the structural relations specified in the overall model were reasonably well reproduced within each country. The standardized factor loadings of the measurement model in Step 1 and the SEM in Step 2 per country site are presented in Tables G.2 to G.7 and Tables H.2 to H.7 of the supporting information, respectively.

The indirect relations between the 7Cs of PYD and life satisfaction via hope across the six countries are presented in Table 5. Overall, the results highlight both commonalities and country‐specific nuances. Controlling for the influence of socio‐demographic covariates, *connection* consistently showed statistically significant indirect associations with life satisfaction through hope across the six countries. *Confidence* exhibited significant indirect associations with life satisfaction via hope in all countries, except for Thailand. Similarly, *creativity* showed significant indirect associations with life satisfaction via hope in all countries, except for Singapore. Although it was not detected in the overall SEM, *character* showed a significant indirect association with life satisfaction through hope in the Philippines, even after controlling for the socio‐demographic covariates. No significant indirect associations were detected for competence, caring, and contribution. All indirect paths between the 7Cs of PYD and life satisfaction via hope are presented in Tables J.1 to J.7 of the supporting information.

## DISCUSSION

Anchored in the 7Cs model of Positive Youth Development (PYD) (Dimitrova et al. 2021) and the integrative paradigm of PYD (Buenconsejo & Datu, 2022), the present study examined how specific PYD attributes (i.e., competence, confidence, connection, character, caring, contribution, and creativity) were linked to hope and life satisfaction among samples of emerging adults from six Southeast Asian countries (i.e., Indonesia, Malaysia, the Philippines, Singapore, Thailand, and Vietnam). Addressing longstanding gaps in PYD scholarship, particularly the underrepresentation of Majority World contexts, this study investigated (a) the direct associations between the 7Cs of PYD, hope, and life satisfaction; (b) the indirect pathways through which PYD characteristics relate to life satisfaction via hope; and (c) whether these pathways vary across six Southeast Asian countries. All analyses were conducted while controlling for the influence of gender, age, and education. By simultaneously examining intrapersonal, interpersonal, and ecological PYD resources within a multi‐country sample, this study provides a more comprehensive and context‐sensitive account of thriving among emerging adults in parts of the Majority World.

Overall, the findings support PYD as a multidimensional framework of psychosocial strengths that are associated with youth well‐being through distinct pathways. In the overall SEM, confidence, connection, and creativity showed significant positive associations with hope. Confidence, connection, character, and contribution were also positively associated with life satisfaction. Hope itself was strongly associated with life satisfaction, while confidence, connection, and creativity showed significant indirect associations with life satisfaction through hope. These patterns highlight hope as a plausible psychological pathway linking specific PYD strengths with well‐being. The results further revealed meaningful cross‐national variations in the salience of specific PYD attributes and their pathways to hope and life satisfaction, underscoring the need to contextualize PYD processes within the sociocultural and structural realities of Southeast Asian youth development.

### PYD strengths show distinct associations with Hope and life satisfaction

Consistent with past PYD investigations (Abdul Kadir et al. 2021; Holsen et al. 2017), several PYD dimensions demonstrated significant associations with hope and life satisfaction, even after accounting for socio‐demographic covariates. Confidence, connection, and creativity were positively associated with hope, suggesting that both intrapersonal‐immediate resources (i.e., confidence and creativity) and interpersonal‐proximal resources (i.e., connection) play important roles in fostering hopeful thinking. These findings align with relational developmental systems perspectives, which emphasize that agency, optimism, and goal‐directed motivation emerge from the dynamic interplay between individual strengths and supportive social contexts (Bowers & Bowers, 2023; Callina et al. 2015; Ismailov, 2026). Creativity's association with hope further extends PYD scholarship by highlighting the importance of adaptive problem‐solving and generative thinking in enabling youth to envision positive versions of themselves amid uncertainty about the future (Dimitrova et al. 2021; Said‐Metwaly et al. 2017).

As for life satisfaction, confidence, connection, character, and contribution showed significant positive associations, whereas competence, caring, and creativity were not directly related to young people's satisfaction with life in the overall model. These patterns mirror recent evidence suggesting that certain PYD attributes may not uniformly promote well‐being across contexts or outcomes (Buenconsejo & Datu, 2024; Geldhof et al. 2019). For instance, competence may carry heightened performance pressures in some educational systems (Buenconsejo & Datu, 2024), while caring may incur emotional or role‐related burden if not supported by adequate social resources (Geldhof et al. 2019). The nonsignificant direct paths of these Cs to life satisfaction, coupled with their more modest or indirect links via hope, reinforce the importance of examining PYD dimensions individually rather than assuming additive or uniformly positive effects. This is particularly salient in contexts characterized by relatively stronger collectivistic orientations, where sociocultural and familial expectations may shape how strengths are enacted and experienced (Yeung et al. 2018).

### Hope links confidence, connection, and creativity with life satisfaction

In line with the second objective, the results indicated that hope served as a significant indirect pathway linking distinct PYD dimensions to life satisfaction. In the overall SEM, confidence, connection, and creativity showed significant indirect relations with life satisfaction through hope, whereas competence, character, caring, and contribution did not. This pattern is consistent with theoretical propositions that hopeful thinking integrates agency, optimistic pathways of thinking, and relational support in the pursuit of meaningful life goals (Assari et al. 2024; Yarcheski & Mahon, 2016), while also suggesting that not all strengths translate into well‐being via the same psychological mechanism. Prior research examining hope as an indirect pathway has focused largely on the 5Cs (Abdul‐Kadir & Mohd, 2021). Extending this work, the present findings suggest that creativity may also show an indirect association with life satisfaction through hope. Intrapersonal‐immediate PYD assets such as confidence and creativity may foster hope by strengthening young people's beliefs in their capacity to initiate action and generate alternative strategies when confronted by challenges, thereby sustaining motivation toward future goals (Murphy, 2023; Satici, 2016). Similarly, interpersonal‐proximal PYD resources such as connection may promote hope by anchoring young people in supportive relationships that provide guidance, direction, and perseverance, which may promote satisfaction with life (Bowers & Bowers, 2023; Callina et al. 2015; Ismailov, 2026).

The observed patterns of direct and indirect associations further clarify how specific PYD attributes were differentially related to life satisfaction. The significant indirect paths for confidence and connection, combined with their remaining significant direct paths to life satisfaction, indicate that hope partially mediated the relations of these strengths. Aside from hope, young people who feel more confident and well‐connected tend to be more satisfied with their lives because these resources also support well‐being through greater self‐efficacy, self‐esteem, and interpersonal support (Bowers & Bowers, 2023; Murphy, 2023). In contrast, the significant indirect path for creativity, together with its nonsignificant direct path to life satisfaction, suggests that hope fully mediated the relation between creativity and life satisfaction. This result highlights hope as a primary psychological mechanism through which adaptive problem‐solving and generative thinking are translated into greater life satisfaction (Satici, 2016; Yalçın & Malkoç, 2015). Creativity may enable emerging adults to envision novel pathways and solutions when conventional routes are blocked (Said‐Metwaly et al. 2017). Taken together, these indirect associations identify hope as a plausible pathway linking confidence, connection, and creativity with life satisfaction.

Although competence, caring, and contribution did not exhibit significant indirect relations with life satisfaction via hope in the overall model, these findings should be interpreted cautiously and at a general level across all emerging adult samples from the six Southeast Asian countries. First, these PYD dimensions still showed positive bivariate associations with both hope and life satisfaction, indicating that they are not irrelevant to youth well‐being but may exert more modest effects once other strengths and covariates are controlled. Rather than functioning uniformly across all settings, it is possible that their associations may be more context‐dependent, operate through alternative mediators, or become salient under specific cultural, structural, or developmental conditions within Southeast Asian contexts. In high‐pressure educational and career settings, for example, competence may be bound up with performance demands that dampen its link to hope (Buenconsejo & Datu, 2024), and caring or contribution may carry emotional or role‐related burdens when young people are expected to support others despite limited resources (Geldhof et al. 2019). These distinct patterns underscore that PYD attributes may differ in how they relate to hopeful life satisfaction and highlight the importance of identifying when, where, and how particular strengths relate to well‐being.

### Connection shows the most consistent indirect association across countries

Addressing the third objective, the country‐specific analyses revealed both shared and contextually distinct pathways linking PYD attributes, hope, and life satisfaction. Prior to examining these structural relations, the results of the MGCFA demonstrated configural, metric, and scalar invariance for the 7Cs of PYD and hope across the six Southeast Asian countries. These findings provide additional evidence for the cross‐cultural applicability of the 7Cs framework among youth in the Majority World and support the meaningful comparison of PYD processes across diverse settings (Abdul Kadir et al. 2021; Buenconsejo, Ferrer‐Wreder, Dimitrova, et al. 2026; Manrique‐Millones et al. 2021).

At the structural level, connection showed the most consistent pattern, exhibiting significant indirect associations with life satisfaction through hope across all six countries. This pattern suggests that supportive relationships may represent a particularly ubiquitous developmental asset among Southeast Asian emerging adults. Given the collectivistic orientation of many Southeast Asian societies, family cohesion, interpersonal obligations, and social connectedness often serve as important sources of meaning, guidance, and psychological security, thereby nurturing hopeful thinking and well‐being (Bernardo, 2010; Wong et al. 2021; Yeung et al. 2018). During the COVID‐19 pandemic, when disruptions to education, employment, and social routines heightened uncertainty about the future, supportive relationships have become particularly important sources of emotional reassurance and future‐oriented optimism (Buenconsejo, Ferrer‐Wreder, Dimitrova, et al. 2026; Exner‐Cortens et al. 2022). This may help explain why connection emerged as the most consistent pathway to hope and life satisfaction across all six countries.

Beyond connection, confidence and creativity also demonstrated largely consistent, although not universal, indirect associations with life satisfaction through hope. The indirect association for confidence was significant in all countries except Thailand, whereas the indirect association for creativity was significant in all countries except Singapore. These findings suggest that both positive self‐beliefs and adaptive problem‐solving abilities may function as important psychological resources for young people to navigate uncertainty and envision attainable future goals (Murphy, 2023; Said‐Metwaly et al. 2017). At the same time, the exceptions observed in Thailand and Singapore highlight how broader sociocultural and structural conditions may shape the roles of specific PYD attributes. Compared with many Western contexts, Thai society has traditionally emphasized interpersonal harmony, humility, and relational adjustment (McKenzie, 2018), values that may reduce the centrality of individual self‐confidence for future‐oriented well‐being. Positive thinking among Thai youth may also depend more on relational or contextual resources than on personal beliefs of self‐worth (Balthip et al. 2022). In highly structured educational and occupational environments such as Singapore, the benefits of creativity for future‐oriented well‐being may depend on whether young people perceive sufficient opportunities to translate novel ideas into meaningful goals and outcomes (Kwang & Smith, 2004; Leu et al. 2023). Additionally, strict public health measures, educational disruptions, and uncertainty about academic and career trajectories during the COVID‐19 pandemic may have constrained opportunities for Singaporean youth to translate creative capacities into hopeful future planning. Indeed, research shows that the adverse effects of the pandemic have intensified socio‐economic and well‐being disparities among young people in Singapore (Chua et al. 2026).

A more distinct pattern emerged for character, which exhibited a significant indirect association with life satisfaction through hope only among Filipino emerging adults. This finding may reflect the strong integration of moral values, religiosity, and prosocial responsibility in the Filipino context, where ethical conduct is often tied to purpose, future aspirations, and perceptions of a meaningful life (Bernardo, 2010; Buenconsejo & Datu, 2023b). Within a predominantly Christian country like the Philippines, moral commitments and faith‐based worldviews may provide a sense of direction and meaning that sustains optimism about the future despite social and economic adversities (Buenconsejo, Contreras, et al. 2026; Del Castillo & Alino, 2020). Character strengths such as integrity, responsibility, and concern for others may likely foster hopeful thinking among Filipino emerging adults, which may subsequently contribute to greater life satisfaction (Montano & Yabut, 2026).

Competence, caring, and contribution, however, did not exhibit significant indirect effects in any of the country‐specific models despite their positive bivariate associations with hope and life satisfaction. These findings suggest that such strengths may relate to well‐being through alternative pathways or become more salient under specific developmental and contextual conditions not captured in the present study. Given that the samples were recruited through convenience sampling and were not nationally representative, the country‐specific findings should be interpreted cautiously. Nevertheless, the overall pattern of findings suggests that while specific PYD assets, particularly connection, confidence, and creativity, may represent broadly relevant resources for fostering hope and life satisfaction, their developmental significance remains embedded within specific cultural, social, and institutional contexts. These results underscore the importance of advancing culturally relevant PYD scholarship that simultaneously identifies shared developmental assets and recognizes context‐specific pathways to thriving in Majority World settings (Koller & Verma, 2017; Wiium & Dimitrova, 2019).

### Implications for context‐sensitive PYD theory and practice

Theoretically, the present findings extend PYD scholarship by clarifying how specific developmental assets operate through psychological mechanisms within Majority World contexts. By identifying hope as a central pathway linking confidence, connection, and creativity to life satisfaction, this study advances a process‐oriented understanding of PYD that moves beyond static or additive models of youth strengths (Buenconsejo & Datu, 2022; Qi et al. 2020). The findings further suggest that PYD attributes may not relate to well‐being through identical developmental processes. Rather, different strengths may operate through distinct pathways, with some dimensions exhibiting direct relations with life satisfaction and others exerting their influence indirectly through hope.

Prior scholarship has called for greater contextual sensitivity in PYD theorizing (Wiium & Dimitrova, 2019). Consistent with this perspective, the present findings provide additional support for the cross‐cultural applicability of the 7Cs framework while showing that the salience of specific PYD dimensions may vary across Southeast Asian samples. For researchers, these findings highlight the importance of examining both common and context‐specific pathways linking PYD attributes to well‐being, particularly in underrepresented Majority World settings where cultural values, social relationships, and structural opportunities may shape developmental processes in unique ways (Koller & Verma, 2017).

In terms of practice, the findings offer actionable insights for youth practitioners, educators, and policymakers in Southeast Asia. Although cross‐sectional, our results suggest that programs aimed at supporting youth well‐being could consider cultivating hope by strengthening intrapersonal‐immediate assets such as confidence and creativity, alongside interpersonal‐proximal assets such as connection, which showed the most consistent associations with hope and life satisfaction across countries. Schools, universities, and community organizations could consider incorporating opportunities for goal‐setting, future planning, creative problem‐solving, and strengths reflection, while simultaneously fostering supportive relationships through mentoring programs, peer‐support initiatives, and family‐school partnerships. The particularly robust role of connection across all six countries further suggests that programs may benefit from emphasizing positive relationships with families, peers, schools, and communities. Importantly, the observed country‐specific patterns indicate that PYD programs should be adapted to local cultural values, educational systems, and developmental realities rather than relying on one‐size‐fits‐all approaches. Such context‐sensitive strategies are likely to enhance the relevance, sustainability, and effectiveness of PYD initiatives across diverse Southeast Asian settings (Buenconsejo & Datu, 2023a; Wiium & Dimitrova, 2019).

### Limitations and future directions

The present study has methodological limitations that warrant consideration. First, the use of cross‐sectional data precludes causal inferences regarding the directional relations among the 7Cs of PYD, hope, and life satisfaction. Although the proposed model was theoretically grounded, longitudinal or experimental designs are necessary to establish their temporal ordering (Maxwell & Cole, 2007). Second, the constructs were assessed using self‐reported measures, which may be subject to shared method variance and social desirability biases. Third, the emerging adult samples from each country were obtained through convenience sampling and were not nationally representative, with the overall sample consisting predominantly of female participants and university students. Although gender, age, and educational attainment were statistically controlled in the SEM, the sample composition may nevertheless limit the generalizability of the findings to broader youth populations within each country. Consequently, observed cross‐country differences may partially reflect sample characteristics rather than population‐level variations. Fourth, the study focused primarily on emerging adults aged 18 to 25 years. Given that definitions and developmental expectations associated with “youth” and “emerging adulthood” may vary across cultural and geographical contexts, the findings may not fully generalize to younger adolescents or other developmental periods (Arnett, 2024). Lastly, although measurement invariance was supported by the multi‐group analyses, the study relied primarily on quantitative indicators that may not fully capture culturally embedded meanings of PYD constructs, especially competence, character, caring, and contribution.

Future research may address these limitations by adopting longitudinal and mixed‐methods approaches to fully elucidate PYD processes in the Majority World. Longitudinal designs would enable researchers to examine developmental trajectories, reciprocal relations, and long‐term effects of PYD attributes on well‐being outcomes. Incorporating qualitative methods (e.g., interviews, focus group discussions, and youth participatory action research) would allow for a deeper exploration of how young people in diverse contexts interpret and enact PYD strengths in their everyday lives (Koller & Verma, 2017; Wiium & Dimitrova, 2019). Additionally, future studies should strive for more representative and demographically balanced sampling strategies and consider integrating multi‐informant or behavioral indicators to reduce reliance on self‐reports. Future investigations should also examine whether the pathways linking PYD, hope, and life satisfaction differ across developmental stages, particularly among early, middle, and late adolescents, as well as emerging adults. Such work would help clarify whether the developmental processes identified in the present study are consistent across age groups or are specific to particular periods of the life course. Comparative research that explicitly examines structural factors (e.g., educational systems, labor markets, and social policies; Rattanasevee, 2023) with psychosocial processes may further clarify how contextual conditions shape pathways to youth thriving, thereby strengthening the cultural relevance and applied relevance of PYD scholarship in the Majority World.

## CONCLUSION

This study advances Positive Youth Development (PYD) scholarship by demonstrating that the 7Cs function as meaningful yet contextually embedded psychosocial resources that are associated with youth well‐being in Southeast Asia, a diverse region that remains underrepresented in developmental research. By identifying hope as a plausible mechanism linking confidence, connection, and creativity to life satisfaction, the findings clarify how specific developmental assets can translate into well‐being outcomes among young people in the Majority World. The observed cross‐national similarities affirm the broader applicability of the 7Cs framework, while the country‐specific variations underscore the importance of situating PYD processes within sociocultural, institutional, and structural realities that young people navigate in their respective contexts. Collectively, this work moves PYD research beyond universalistic assumptions, offering empirically grounded insights that can inform culturally responsive theory development, measurement, and practice aimed at fostering youth thriving across diverse Southeast Asian contexts.

## AUTHOR CONTRIBUTIONS

**Jet Uy Buenconsejo:** Conceptualization; methodology; software; data curation; formal analysis; visualization; writing – review and editing; writing – original draft. **Laura Ferrer‐Wreder:** Conceptualization; writing – review and editing. **Lu Yu:** Conceptualization; writing – review and editing. **Steven Krauss:** Conceptualization; investigation; project administration; writing – review and editing. **Nor Ba'yah Abdul‐Kadir:** Investigation; project administration; writing – review and editing. **Yue Yu:** Investigation; project administration; writing – review and editing. **John Jamir Benzon R. Aruta:** Investigation; project administration; writing – review and editing. **Angela Oktavia Suryani:** Writing – review and editing; investigation; project administration. **Priyoth Kittiteerasack:** Investigation; project administration; writing – review and editing. **Duong Cong Doanh:** Investigation; writing – review and editing; project administration. **Radosveta Dimitrova:** Conceptualization; data curation; project administration; investigation; supervision; writing – review and editing.

## FUNDING STATEMENT

Open Access was provided by the Department of Applied Social Sciences of The Hong Kong Polytechnic University.

## CONFLICT OF INTEREST STATEMENT

The authors declare no conflicts of interest.

## ETHICS STATEMENT

The study complied with the ethical standards for conducting research with human subjects as specified in the 1964 Helsinki declaration and its later amendments. This study was part of a larger project called “International and Multidimensional Perspectives on the Impact of COVID‐19” or “IMPACT‐C19”, which was led by the Research Initiatives Work Group of the American Psychological Association Interdivisional Task Force on the Pandemic. Ethical approval was obtained from the authors' respective university in each country site.

## CONSENT

Informed consent from the participants was obtained before the data collection.

## AUTHORS' REFLEXIVITY STATEMENT

The authors of this study represent a diverse and interdisciplinary team of scholars with personal, cultural, and/or professional ties to the Southeast Asian contexts examined in this research. Several authors are native to, reside in, or have long‐standing academic and community engagement within Indonesia, Malaysia, the Philippines, Singapore, Thailand, and Vietnam. This proximity to local cultural norms, languages, and developmental systems informed both the conceptual framing and contextual interpretation of the study. Our positionalities include female and male, majority and minority status within our respective nations, and from a disciplinary standpoint span developmental psychology, cross‐cultural psychology, education, public health, economics, and youth studies, allowing for a holistic and culturally responsive approach to Positive Youth Development. We recognize the potential influence of our own values, academic training (both within and outside the Majority World), and institutional affiliations on how research was framed, data were interpreted, and findings were situated. Throughout the research process, we prioritized cultural grounding, open dialogue across national boundaries, and a commitment to strengths‐based perspectives on youth. This reflexive stance aimed to ensure that the study did not merely apply PYD models to Southeast Asia but engaged meaningfully with how such models may be expanded, adapted, or challenged by local realities.

## Supporting information


**Supplementary Material A.** Table A.1–A.6.
**Supplementary Material B**. Table B.1–B.6.
**Supplementary Material C**. Table C.1–C.2.
**Supplementary Material D**. Table D.1–D.7.
**Supplementary Material E**. Table E.1–E.7.
**Supplementary Material F**. Table F.1–F.7.
**Supplementary Material G**. Table G.1–G.7.
**Supplementary Material H**. Table H.1–H.7.
**Supplementary Material I**. Table I.1–I.7.
**Supplementary Material J**. Table J.1–J.7.
**Supplementary Material K**. Table K.

## Data Availability

The data that support the findings of this study are available on request from the corresponding author. The data are not publicly available due to privacy or ethical restrictions.

## Footnotes

^1^
We have tested an alternative structural equation model specifying hope as the predictor variable, the 7Cs of PYD as mediators, and life satisfaction as the outcome variable. Although the model demonstrated acceptable global fit indices, several theoretically inconsistent negative direct and indirect effects that emerged, suggesting possible suppression effects when hope is treated as the predictor variable. Accordingly, we retained the hypothesized model specifying the 7Cs as antecedent variables, hope as the intervening variable, and life satisfaction as the outcome. Detailed results of the alternative model are presented in Supplementary Material K.
