# Supplementary material for: Pathways of positive youth development, hope, and life satisfaction: A multi‐country study among Southeast Asian youth
Source: J Res Adolesc. 2026 Nov 5;36(4):e70313. doi: 10.1111/jora.70313 (PMC13740660; doi:10.1111/jora.70313)
Supplement: Supplementary file 1 [file JORA-36-0-s001.pdf]

Pathways of Positive Youth Development, Hope, and Life Satisfaction: A Multi-Country Study Among Southeast Asian Youth

Jet Uy Buenconsejo, Laura Ferrer-Wreder, Lu Yu, Steven Krauss, Nor Ba'yah Abdul-Kadir, Yue Yu, John Jamir Benzon R. Aruta, Angela Oktavia Suryani, Priyoth Kittiteerasack, Duong Cong Doanh, & Radosveta Dimitrova

Supplementary Materials

Supplementary Material A

Table A.1. Descriptive Statistics and Reliability Coefficients of the 7Cs of Positive Youth Development, Hope, and Life Satisfaction Among Youth from Indonesia (n = 227)
Table A.2. Descriptive Statistics and Reliability Coefficients of the 7Cs of Positive Youth Development, Hope, and Life Satisfaction Among Youth from Malaysia (n = 280)
Table A.3. Descriptive Statistics and Reliability Coefficients of the 7Cs of Positive Youth Development, Hope, and Life Satisfaction Among Youth from the Philippines (n = 479)
Table A.4. Descriptive Statistics and Reliability Coefficients of the 7Cs of Positive Youth Development, Hope, and Life Satisfaction Among Youth from Singapore (n = 247)
Table A.5. Descriptive Statistics and Reliability Coefficients of the 7Cs of Positive Youth Development, Hope, and Life Satisfaction Among Youth from Thailand (n = 486)
Table A.6. Descriptive Statistics and Reliability Coefficients of the 7Cs of Positive Youth Development, Hope, and Life Satisfaction Among Youth from Vietnam (n = 248)

Supplementary Material B

Table B.1. Bivariate Correlations of the 7Cs of Positive Youth Development, Hope, and Life Satisfaction Among Youth from Indonesia (n = 227)
Table B.2. Bivariate Correlations of the 7Cs of Positive Youth Development, Hope, and Life Satisfaction Among Youth from Malaysia (n = 280)
Table B.3. Bivariate Correlations of the 7Cs of Positive Youth Development, Hope, and Life Satisfaction Among Youth from the Philippines (n = 479)
Table B.4. Bivariate Correlations of the 7Cs of Positive Youth Development, Hope, and Life Satisfaction Among Youth from Singapore (n = 247)
Table B.5. Bivariate Correlations of the 7Cs of Positive Youth Development, Hope, and Life Satisfaction Among Youth from Thailand (n = 486)
Table B.6. Bivariate Correlations of the 7Cs of Positive Youth Development, Hope, and Life Satisfaction Among Youth from Vietnam (n = 248)

Supplementary Material C

Table C.1. Results of the Multi-group Confirmatory Factor Analyses on the Measurement Model of the 7Cs of Positive Youth Development Across the Six Southeast Asian Countries
Table C.2. Results of the Multi-group Confirmatory Factor Analyses on the Measurement Model of Hope Across the Six Southeast Asian Countries

Supplementary Material D

Table D.1. Factor Loadings of the 7Cs Model of Positive Youth Development Using the Overall Youth Sample (n = 1,967)

Table D.2. Factor Loadings of the 7Cs Model of Positive Youth Development Using the Youth Sample from Indonesia (n = 227)
Table D.3. Factor Loadings of the 7Cs Model of Positive Youth Development Using the Youth Sample from Malaysia (n = 280)
Table D.4. Factor Loadings of the 7Cs Model of Positive Youth Development Using the Youth Sample from the Philippines (n = 479)
Table D.5. Factor Loadings of the 7Cs Model of Positive Youth Development Using the Youth Sample from Singapore (n = 247)
Table D.6. Factor Loadings of the 7Cs Model of Positive Youth Development Using the Youth Sample from Thailand (n = 486)
Table D.7. Factor Loadings of the 7Cs Model of Positive Youth Development Using the Youth Sample from Vietnam (n = 248)

Supplementary Material E

Table E.1. Factor Loadings of the Measurement Model of Hope Using the Overall Youth Sample (n = 1,967)
Table E.2. Factor Loadings of the Measurement Model of Hope Using the Youth Sample from Indonesia (n = 227)
Table E.3. Factor Loadings of the Measurement Model of Hope Using the Youth Sample from Malaysia (n = 280)
Table E.4. Factor Loadings of the Measurement Model of Hope Using the Youth Sample from the Philippines (n = 479)
Table E.5. Factor Loadings of the Measurement Model of Hope Using the Youth Sample from Singapore (n = 247)
Table E.6. Factor Loadings of the Measurement Model of Hope Using the Youth Sample from Thailand (n = 486)
Table E.7. Factor Loadings of the Measurement Model of Hope Using the Youth Sample from Vietnam (n = 248)

Supplementary Material F

Table F.1. Factor Loadings of the Measurement Model of Life Satisfaction Using the Overall Youth Sample (n = 1,967)
Table F.2. Factor Loadings of the Measurement Model of Life Satisfaction Using the Youth Sample from Indonesia (n = 227)
Table F.3. Factor Loadings of the Measurement Model of Life Satisfaction Using the Youth Sample from Malaysia (n = 280)
Table F.4. Factor Loadings of the Measurement Model of Life Satisfaction Using the Youth Sample from the Philippines (n = 479)
Table F.5. Factor Loadings of the Measurement Model of Life Satisfaction Using the Youth Sample from Singapore (n = 247)
Table F.6. Factor Loadings of the Measurement Model of Life Satisfaction Using the Youth Sample from Thailand (n = 486)
Table F.7. Factor Loadings of the Measurement Model of Life Satisfaction Using the Youth Sample from Vietnam (n = 248)

Supplementary Material G

Table G.1. Factor Loadings of the Measurement Model (Step 1) of the 7Cs of Positive Youth Development, Hope, and Life Satisfaction Using the Overall Youth Sample (n = 1,967)
Table G.2. Factor Loadings of the Measurement Model (Step 1) of the 7Cs of Positive Youth Development, Hope, and Life Satisfaction Using the Youth Sample from Indonesia (n = 227)
Table G.3. Factor Loadings of the Measurement Model (Step 1) of the 7Cs of Positive Youth Development, Hope, and Life Satisfaction Using the Youth Sample from Malaysia (n = 280)
Table G.4. Factor Loadings of the Measurement Model (Step 1) of the 7Cs of Positive Youth Development, Hope, and Life Satisfaction Using the Youth Sample from the Philippines (n = 479)

Table G.5. Factor Loadings of the Measurement Model (Step 1) of the 7Cs of Positive Youth Development, Hope, and Life Satisfaction Using the Youth Sample from Singapore (n = 247)
Table G.6. Factor Loadings of the Measurement Model (Step 1) of the 7Cs of Positive Youth Development, Hope, and Life Satisfaction Using the Youth Sample from Thailand (n = 486)
Table G.7. Factor Loadings of the Measurement Model (Step 1) of the 7Cs of Positive Youth Development, Hope, and Life Satisfaction Using the Youth Sample from Vietnam (n = 248)

Supplementary Material H

Table H.1. Factor Loadings of the Structural Equation Model (Step 2) of the 7Cs of Positive Youth Development, Hope, and Life Satisfaction Controlling for Gender, Age, and Education Using the Overall Youth Sample (n = 1,967)
Table H.2. Factor Loadings of the Structural Equation Model (Step 2) of the 7Cs of Positive Youth Development, Hope, and Life Satisfaction Controlling for Gender, Age, and Education Using the Youth Sample from Indonesia (n = 227)
Table H.3. Factor Loadings of the Structural Equation Model (Step 2) of the 7Cs of Positive Youth Development, Hope, and Life Satisfaction Controlling for Gender, Age, and Education Using the Youth Sample from Malaysia (n = 280)
Table H.4. Factor Loadings of the Structural Equation Model (Step 2) of the 7Cs of Positive Youth Development, Hope, and Life Satisfaction Controlling for Gender, Age, and Education Using the Youth Sample from the Philippines (n = 479)
Table H.5. Factor Loadings of the Structural Equation Model (Step 2) of the 7Cs of Positive Youth Development, Hope, and Life Satisfaction Controlling for Gender, Age, and Education Using the Youth Sample from Singapore (n = 247)
Table H.6. Factor Loadings of the Structural Equation Model (Step 2) of the 7Cs of Positive Youth Development, Hope, and Life Satisfaction Controlling for Gender, Age, and Education Using the Youth Sample from Thailand (n = 486)
Table H.7. Factor Loadings of the Structural Equation Model (Step 2) of the 7Cs of Positive Youth Development, Hope, and Life Satisfaction Controlling for Gender, Age, and Education Using the Youth Sample from Vietnam (n = 248)

Supplementary Material I

Table I.1. Path Coefficients of the Structural Equation Model (Step 2) of the 7Cs of Positive Youth Development, Hope, and Life Satisfaction Controlling for Gender, Age, and Education Using the Overall Youth Sample (n = 1,967)
Table I.2. Path Coefficients of the Structural Equation Model (Step 2) of the 7Cs of Positive Youth Development, Hope, and Life Satisfaction Controlling for Gender, Age, and Education Using the Youth Sample from Indonesia (n = 227)
Table I.3. Path Coefficients of the Structural Equation Model (Step 2) of the 7Cs of Positive Youth Development, Hope, and Life Satisfaction Controlling for Gender, Age, and Education Using the Youth Sample from Malaysia (n = 280)
Table I.4. Path Coefficients of the Structural Equation Model (Step 2) of the 7Cs of Positive Youth Development, Hope, and Life Satisfaction Controlling for Gender, Age, and Education Using the Youth Sample from the Philippines (n = 479)
Table I.5. Path Coefficients of the Structural Equation Model (Step 2) of the 7Cs of Positive Youth Development, Hope, and Life Satisfaction Controlling for Gender, Age, and Education Using the Youth Sample from Singapore (n = 247)

Table I.6. Path Coefficients of the Structural Equation Model (Step 2) of the 7Cs of Positive Youth Development, Hope, and Life Satisfaction Controlling for Gender, Age, and Education Using the Youth Sample from Thailand (n = 486)
Table I.7. Path Coefficients of the Structural Equation Model (Step 2) of the 7Cs of Positive Youth Development, Hope, and Life Satisfaction Controlling for Gender, Age, and Education Using the Youth Sample from Vietnam (n = 248)

Supplementary Material J

Table J.1. Indirect Relations of the 7Cs of Positive Youth Development on Life Satisfaction via Hope Controlling for Gender, Age, and Education Using the Overall Youth Sample (n = 1,967)
Table J.2. Indirect Relations of the 7Cs of Positive Youth Development on Life Satisfaction via Hope Controlling for Gender, Age, and Education Using the Youth Sample from Indonesia (n = 227)
Table J.3. Indirect Relations of the 7Cs of Positive Youth Development on Life Satisfaction via Hope Controlling for Gender, Age, and Education Using the Youth Sample from Malaysia (n = 280)
Table J.4. Indirect Relations of the 7Cs of Positive Youth Development on Life Satisfaction via Hope Controlling for Gender, Age, and Education Using the Youth Sample from the Philippines (n = 479)
Table J.5. Indirect Relations of the 7Cs of Positive Youth Development on Life Satisfaction via Hope Controlling for Gender, Age, and Education Using the Youth Sample from Singapore (n = 247)
Table J.6. Indirect Relations of the 7Cs of Positive Youth Development on Life Satisfaction via Hope Controlling for Gender, Age, and Education Using the Youth Sample from Thailand (n = 486)
Table J.7. Indirect Relations of the 7Cs of Positive Youth Development on Life Satisfaction via Hope Controlling for Gender, Age, and Education Using the Youth Sample from Vietnam (n = 248)

Supplementary Material K

Table K. Indirect Relations of Hope on Life Satisfaction via the 7Cs of Positive Youth Development Controlling for Gender, Age, and Education Using the Overall Youth Sample (Alternative Structural Equation Modelling)

Supplementary Material A

Table A.1.
Descriptive Statistics and Reliability Coefficients of the 7Cs of Positive Youth Development, Hope, and Life Satisfaction Among Youth from Indonesia

Variable	N	Minimum	Minimum	Mean	Standard Deviation	Skewness	Kurtosis	Cronbach's α	McDonald's ω
Competence	227	1.00	5.00	3.21	0.68	0.13	0.18	0.72	0.74
Confidence	227	1.00	5.00	3.38	0.89	-0.22	-0.14	0.86	0.87
Character	227	2.50	5.00	3.87	0.49	0.10	0.18	0.73	0.74
Caring	227	2.33	5.00	4.17	0.58	-0.26	-0.07	0.83	0.84
Connection	227	2.00	5.00	3.59	0.65	0.16	-0.11	0.78	0.79
Contribution	227	1.00	5.00	2.61	0.88	0.36	0.01	0.72	0.80
Creativity	227	1.89	5.00	3.47	0.60	0.20	0.17	0.89	0.93
Hope	227	1.50	5.00	3.75	0.58	-0.27	0.61	0.88	0.90
Life Satisfaction	227	1.00	7.00	4.12	1.24	-0.17	-0.37	0.84	0.85

Table A.2.
Descriptive Statistics and Reliability Coefficients of the 7Cs of Positive Youth Development, Hope, and Life Satisfaction Among Youth from Malaysia

Variable	N	Minimum	Minimum	Mean	Standard Deviation	Skewness	Kurtosis	Cronbach's α	McDonald's ω
Competence	280	1.33	5.00	3.51	0.80	0.01	-0.26	0.76	0.78
Confidence	280	1.00	5.00	3.62	0.95	-0.46	-0.14	0.83	0.85
Character	280	2.75	5.00	4.00	0.60	0.02	-0.83	0.78	0.80
Caring	280	2.67	5.00	4.18	0.62	-0.19	-0.98	0.73	0.78
Connection	280	1.50	5.00	3.70	0.85	-0.09	-0.77	0.75	0.76
Contribution	280	1.00	5.00	2.61	0.94	0.56	-0.43	0.81	0.88
Creativity	280	1.00	5.00	3.48	0.85	-0.08	0.00	0.96	0.95
Hope	280	1.25	5.00	3.93	0.75	-0.52	0.20	0.93	0.95
Life Satisfaction	280	1.00	7.00	4.90	1.29	-0.38	0.21	0.87	0.89

Table A.3.
Descriptive Statistics and Reliability Coefficients of the 7Cs of Positive Youth Development, Hope, and Life Satisfaction Among Youth from the Philippines

Variable	N	Minimum	Minimum	Mean	Standard Deviation	Skewness	Kurtosis	Cronbach's α	McDonald's ω
Competence	479	1.00	5.00	3.23	0.75	-0.03	-0.15	0.77	0.80
Confidence	479	1.00	5.00	3.45	0.90	-0.52	-0.15	0.82	0.82
Character	479	1.00	5.00	4.13	0.54	-1.39	2.63	0.74	0.75
Caring	479	1.00	5.00	4.30	0.68	-1.29	1.33	0.89	0.89
Connection	479	1.00	5.00	3.75	0.70	-0.42	0.31	0.74	0.76
Contribution	479	1.00	5.00	2.74	0.89	0.30	-0.35	0.73	0.83
Creativity	479	1.22	5.00	3.64	0.66	-0.49	0.64	0.91	0.94
Hope	479	1.50	5.00	3.90	0.62	-0.50	0.79	0.90	0.93
Life Satisfaction	479	1.00	7.00	4.51	1.40	-0.62	-0.24	0.86	0.86

Table A.4.
Descriptive Statistics and Reliability Coefficients of the 7Cs of Positive Youth Development, Hope, and Life Satisfaction Among Youth from Singapore

Variable	N	Minimum	Minimum	Mean	Standard Deviation	Skewness	Kurtosis	Cronbach's α	McDonald's ω
Competence	247	1.00	4.33	2.85	0.69	-0.07	-0.40	0.72	0.74
Confidence	247	1.00	5.00	3.21	0.87	-0.41	0.03	0.82	0.83
Character	247	1.25	5.00	3.87	0.53	-0.70	2.30	0.76	0.77
Caring	247	1.00	5.00	4.15	0.59	-0.70	2.80	0.83	0.85
Connection	247	1.25	5.00	3.58	0.62	-0.53	1.13	0.70	0.73
Contribution	247	1.00	4.67	2.35	0.78	0.48	-0.20	0.79	0.81
Creativity	247	1.00	5.00	3.43	0.75	-0.36	0.57	0.94	0.96
Hope	247	1.00	5.00	3.59	0.64	-0.70	1.53	0.90	0.92
Life Satisfaction	247	1.00	7.00	4.38	1.34	-0.55	-0.27	0.88	0.88

Table A.5.

Descriptive Statistics and Reliability Coefficients of the 7Cs of Positive Youth Development, Hope, and Life Satisfaction Among Youth from Thailand

Variable	N	Minimum	Minimum	Mean	Standard Deviation	Skewness	Kurtosis	Cronbach's α	McDonald's ω
Competence	486	1.00	5.00	3.01	0.69	0.29	0.20	0.74	0.78
Confidence	486	1.33	5.00	3.66	0.81	-0.30	-0.40	0.80	0.83
Character	486	2.00	5.00	3.75	0.67	-0.20	-0.40	0.71	0.74
Caring	486	2.00	5.00	3.95	0.77	-0.43	-0.44	0.88	0.88
Connection	486	1.75	5.00	3.62	0.74	-0.08	-0.51	0.72	0.80
Contribution	486	1.00	5.00	2.80	0.96	0.16	-0.50	0.84	0.89
Creativity	486	1.67	5.00	3.50	0.73	-0.02	-0.45	0.94	0.96
Hope	486	2.33	5.00	3.71	0.67	0.11	-0.88	0.92	0.94
Life Satisfaction	486	1.00	7.00	3.99	1.40	-0.02	-0.52	0.89	0.89

Table A.6.

Descriptive Statistics and Reliability Coefficients of the 7Cs of Positive Youth Development, Hope, and Life Satisfaction Among Youth from Vietnam

Variable	N	Minimum	Minimum	Mean	Standard Deviation	Skewness	Kurtosis	Cronbach's α	McDonald's ω
Competence	248	1.00	5.00	3.24	0.70	0.12	0.18	0.71	0.74
Confidence	248	1.67	5.00	3.62	0.72	-0.07	-0.31	0.76	0.78
Character	248	1.00	5.00	3.47	0.54	-0.16	1.86	0.76	0.76
Caring	248	2.00	5.00	3.78	0.66	-0.20	0.29	0.82	0.83
Connection	248	1.50	5.00	3.62	0.64	-0.17	0.15	0.73	0.78
Contribution	248	1.00	5.00	2.84	0.93	0.15	-0.53	0.84	0.88
Creativity	248	1.56	5.00	3.38	0.64	0.26	0.35	0.93	0.95
Hope	248	1.08	5.00	3.61	0.61	-0.38	1.29	0.92	0.94
Life Satisfaction	248	1.00	7.00	4.33	1.16	-0.01	-0.41	0.88	0.89

Supplementary Material B

Table B.1.
Bivariate Correlations of the 7Cs of Positive Youth Development, Hope, and Life Satisfaction Among Youth from Indonesia (n = 227)

	Competence	Confidence	Character	Caring	Connection	Contribution	Creativity	Hope	Life Satisfaction
Competence	--								
Confidence	.496**	--							
Character	.349**	.497**	--						
Caring	0.076	0.065	.369**	--					
Connection	.538**	.543**	.474**	.298**	--				
Contribution	.247**	.208**	.173**	.140*	.300**	--			
Creativity	.351**	.301**	.349**	.229**	.331**	.311**	--		
Hope	.361**	.594**	.395**	.254**	.588**	.337**	.430**	--	
Life Satisfaction	.250**	.516**	.247**	0.038	.419**	0.11	.258**	.438**	--

**. Correlation is significant at the 0.01 level (2-tailed).

*. Correlation is significant at the 0.05 level (2-tailed).

Table B.2.
Bivariate Correlations of the 7Cs of Positive Youth Development, Hope, and Life Satisfaction Among Youth from Malaysia (n = 280)

	Competence	Confidence	Character	Caring	Connection	Contribution	Creativity	Hope	Life Satisfaction
Competence	--								
Confidence	.613**	--							
Character	.460**	.440**	--						
Caring	.293**	.268**	.525**	--					
Connection	.608**	.639**	.442**	.293**	--				
Contribution	.215**	.176**	.217**	.198**	.311**	--			
Creativity	.447**	.430**	.428**	.266**	.479**	.316**	--		
Hope	.524**	.732**	.516**	.335**	.698**	.230**	.523**	--	
Life Satisfaction	.468**	.658**	.378**	.272**	.599**	.161**	.342**	.696**	--

**. Correlation is significant at the 0.01 level (2-tailed).

Table B.3.
Bivariate Correlations of the 7Cs of Positive Youth Development, Hope, and Life Satisfaction Among Youth from the Philippines (n = 479)

	Competence	Confidence	Character	Caring	Connection	Contribution	Creativity	Hope	Life Satisfaction
Competence	--								
Confidence	.439**	--							
Character	.352**	.408**	--						
Caring	.207**	.140**	.602**	--					
Connection	.564**	.436**	.449**	.355**	--				
Contribution	.216**	0.038	.191**	.198**	.311**	--			
Creativity	.384**	.202**	.334**	.279**	.365**	.313**	--		
Hope	.426**	.539**	.424**	.300**	.526**	.200**	.386**	--	
Life Satisfaction	.315**	.485**	.167**	0.058	.420**	0.048	.197**	.472**	--

**. Correlation is significant at the 0.01 level (2-tailed).

Table B.4.
Bivariate Correlations of the 7Cs of Positive Youth Development, Hope, and Life Satisfaction Among Youth from Singapore (n = 247)

	Competence	Confidence	Character	Caring	Connection	Contribution	Creativity	Hope	Life Satisfaction
Competence	--								
Confidence	.413**	--							
Character	.215**	.351**	--						
Caring	.149*	0.119	.497**	--					
Connection	.374**	.571**	.437**	.298**	--				
Contribution	.155*	0.075	.194**	.175**	.230**	--			
Creativity	.231**	.228**	.240**	.214**	.183**	.131*	--		
Hope	.441**	.702**	.518**	.303**	.662**	.172**	.305**	--	
Life Satisfaction	.306**	.621**	.358**	.172**	.486**	0.019	0.106	.587**	--

**. Correlation is significant at the 0.01 level (2-tailed); *. Correlation is significant at the 0.05 level (2-tailed).

Table B.5.
Bivariate Correlations of the 7Cs of Positive Youth Development, Hope, and Life Satisfaction Among Youth from Thailand (n = 486)

	Competence	Confidence	Character	Caring	Connection	Contribution	Creativity	Hope	Life Satisfaction
Competence	--								
Confidence	.330**	--							
Character	.311**	.617**	--						
Caring	.202**	.421**	.667**	--					
Connection	.504**	.596**	.550**	.543**	--				
Contribution	.192**	.257**	.261**	.253**	.347**	--			
Creativity	.316**	.361**	.400**	.314**	.420**	.357**	--		
Hope	.370**	.553**	.485**	.409**	.607**	.275**	.474**	--	
Life Satisfaction	.335**	.463**	.286**	.186**	.458**	.214**	.293**	.514**	--

**. Correlation is significant at the 0.01 level (2-tailed).

Table B.6.
Bivariate Correlations of the 7Cs of Positive Youth Development, Hope, and Life Satisfaction Among Youth from Vietnam (n = 248)

	Competence	Confidence	Character	Caring	Connection	Contribution	Creativity	Hope	Life Satisfaction
Competence	--								
Confidence	.493**	--							
Character	.479**	.555**	--						
Caring	.290**	.360**	.567**	--					
Connection	.557**	.499**	.478**	.582**	--				
Contribution	.389**	.266**	.223**	.188**	.387**	--			
Creativity	.457**	.381**	.452**	.350**	.394**	.343**	--		
Hope	.382**	.534**	.446**	.452**	.535**	.321**	.621**	--	
Life Satisfaction	.314**	.418**	.368**	.394**	.361**	.244**	.337**	.482**	--

**. Correlation is significant at the 0.01 level (2-tailed).

Supplementary Material C

Table C.1.
Results of the Multi-group Confirmatory Factor Analyses on the Measurement Model of the 7Cs of Positive Youth Development Across the Six Southeast Asian Countries (n = 1,967)

Model	χ^2	*df*	CFI	RMSEA	SRMR	ΔCFI	ΔRMSEA	ΔSRMR	Invariant?
Overall Model	4907.574	443	0.985	0.069	0.056	--	--	--	--
Indonesia	1006.969	443	0.963	0.064	0.066	--	--	--	--
Malaysia	989.910	443	0.993	0.063	0.068	--	--	--	--
Philippines	1798.587	443	0.975	0.065	0.057	--	--	--	--
Singapore	826.966	443	0.989	0.063	0.069	--	--	--	--
Thailand	1519.560	443	0.988	0.065	0.059	--	--	--	--
Vietnam	1269.770	443	0.975	0.065	0.061	--	--	--	--
Configural Model	4388.291	2658	0.975	0.042	0.063	--	--	--	Yes
Metric Model	4474.866	2783	0.968	0.046	0.072	0.007	-0.004	-0.009	Yes
Scalar Model	5319.426	2908	0.959	0.057	0.079	0.009	-0.011	-0.007	Yes

Note: $\chi 2$ = chi-square test statistic; *df* = degrees of freedom; CFI = comparative fit index; RMSEA = root mean square error of approximation; SRMR = standardized root mean square residual; ΔCFI = change in comparative fit index; ΔRMSEA = change in root mean square error of approximation; ΔSRMR = change in standardized root mean square residual.

Table C.2.
Results of the Multi-group Confirmatory Factor Analyses on the Measurement Model of Hope Across the Six Southeast Asian Countries (n = 1,967)

Model	χ^2	*df*	CFI	RMSEA	SRMR	ΔCFI	ΔRMSEA	ΔSRMR	Invariant?
Overall Model	1493.132	54	0.993	0.058	0.046	--	--	--	--
Indonesia	302.700	54	0.975	0.074	0.060	--	--	--	--
Malaysia	262.964	54	0.997	0.051	0.045	--	--	--	--
Philippines	483.377	54	0.990	0.060	0.061	--	--	--	--
Singapore	226.484	54	0.993	0.074	0.060	--	--	--	--
Thailand	659.231	54	0.987	0.052	0.067	--	--	--	--
Vietnam	340.311	54	0.992	0.064	0.061	--	--	--	--
Configural Model	857.333	324	0.987	0.048	0.053	--	--	--	Yes
Metric Model	767.815	379	0.979	0.046	0.061	0.008	0.002	-0.008	Yes
Scalar Model	1069.023	434	0.976	0.060	0.062	0.003	-0.014	-0.001	Yes

Note: χ2 = chi-square test statistic; *df* = degrees of freedom; CFI = comparative fit index; RMSEA = root mean square error of approximation; SRMR = standardized root mean square residual; ΔCFI = change in comparative fit index; ΔRMSEA = change in root mean square error of approximation; ΔSRMR = change in standardized root mean square residual.

Supplementary Material D

Table D.1.
Factor Loadings of the 7Cs Model of Positive Youth Development Using the Overall Youth Sample (n = 1,967)

Items	*β*	*b*	SE	z-value	*p*-value	95% ULCI	95% LLCI
Competence =~							
Comp_1	0.653	0.653	0.018	35.590	< .001	0.617	0.689
Comp_2	0.727	0.727	0.017	42.984	< .001	0.694	0.761
Comp_3	0.426	0.426	0.022	19.156	< .001	0.382	0.569
Confidence =~							
Conf_1	0.757	0.757	0.014	55.071	< .001	0.730	0.784
Conf_2	0.761	0.761	0.013	60.249	< .001	0.737	0.786
Conf_3	0.917	0.917	0.011	85.262	< .001	0.896	0.938
Character =~							
Char_1	0.492	0.492	0.021	18.600	< .001	0.351	0.533
Char_2	0.735	0.735	0.014	53.170	< .001	0.708	0.762
Char_3	0.763	0.763	0.013	56.910	< .001	0.737	0.790
Char_4	0.603	0.603	0.016	37.393	< .001	0.572	0.635
Caring =~							
Care_1	0.736	0.736	0.013	55.160	< .001	0.710	0.762
Care_2	0.876	0.876	0.009	95.554	< .001	0.858	0.894
Care_3	0.953	0.953	0.008	117.280	< .001	0.937	0.969
Connection =~							
Conn_1	0.685	0.685	0.015	45.850	< .001	0.656	0.715
Conn_2	0.746	0.746	0.014	53.779	< .001	0.719	0.773
Conn_3	0.679	0.679	0.014	48.760	< .001	0.652	0.706
Conn_4	0.573	0.573	0.020	29.354	< .001	0.535	0.611
Contribution =~							

Cont_1	0.693	0.693	0.020	34.585	< .001	0.654	0.733
Cont_2	0.694	0.694	0.017	40.270	< .001	0.660	0.728
Cont_3	0.655	0.655	0.021	30.671	< .001	0.613	0.697
Cont_4	0.704	0.704	0.017	41.957	< .001	0.671	0.737
Cont_5	0.672	0.672	0.019	36.182	< .001	0.635	0.708
Cont_6	0.657	0.657	0.020	32.086	< .001	0.617	0.698
Creativity =~							
Crea_1	0.720	0.720	0.011	64.719	< .001	0.698	0.742
Crea_2	0.834	0.834	0.007	111.792	< .001	0.819	0.848
Crea_3	0.845	0.845	0.007	124.366	< .001	0.832	0.858
Crea_4	0.779	0.779	0.009	85.831	< .001	0.761	0.797
Crea_5	0.828	0.828	0.008	103.483	< .001	0.812	0.844
Crea_6	0.820	0.820	0.007	112.354	< .001	0.806	0.834
Crea_7	0.787	0.787	0.009	92.178	< .001	0.770	0.803
Crea_8	0.920	0.920	0.004	212.234	< .001	0.912	0.929
Crea_9	0.924	0.924	0.004	214.182	< .001	0.915	0.932

Note: β = standardized coefficient; *b* = unstandardized coefficient; SE = standard error; ULCI = upper limit confidence interval; LLCI = lower limit confidence interval.

Table D.2.
Factor Loadings of the 7Cs Model of Positive Youth Development Using the Youth Sample from Indonesia (n = 227)

Items	*β*	*b*	SE	z-value	*p*-value	95% ULCI	95% LLCI
Competence =~							
Comp_1	0.721	0.696	0.063	11.057	< .001	0.573	0.820
Comp_2	0.645	0.484	0.059	8.257	< .001	0.369	0.599
Comp_3	0.451	0.484	0.099	3.894	< .001	0.191	0.578
Confidence =~							
Conf_1	0.803	0.850	0.063	13.595	< .001	0.727	0.972
Conf_2	0.809	0.806	0.063	12.736	< .001	0.682	0.930
Conf_3	0.865	0.822	0.053	15.480	< .001	0.718	0.926
Character =~							
Char_1	0.246	0.259	0.089	2.913	< .001	0.085	0.333
Char_2	0.461	0.357	0.054	6.626	< .001	0.252	0.463
Char_3	0.561	0.442	0.055	7.985	< .001	0.333	0.650
Char_4	0.535	0.367	0.053	6.915	< .001	0.263	0.671
Caring =~							
Care_1	0.760	0.529	0.051	10.337	< .001	0.429	0.630
Care_2	0.745	0.477	0.044	10.930	< .001	0.391	0.562
Care_3	0.866	0.594	0.050	11.789	< .001	0.495	0.692
Connection =~							
Conn_1	0.608	0.529	0.061	8.641	< .001	0.409	0.649
Conn_2	0.552	0.523	0.064	8.119	< .001	0.396	0.649
Conn_3	0.621	0.580	0.069	8.430	< .001	0.445	0.714
Conn_4	0.509	0.442	0.056	7.894	< .001	0.332	0.551
Contribution =~							
Cont_1	0.423	0.554	0.112	4.942	< .001	0.334	0.773
Cont_2	0.459	0.583	0.121	4.836	< .001	0.347	0.820

Cont_3	0.633	0.907	0.105	8.658	< .001	0.701	1.112
Cont_4	0.728	0.891	0.089	10.023	< .001	0.717	1.066
Cont_5	0.504	0.717	0.111	6.461	< .001	0.500	0.935
Cont_6	0.508	0.769	0.115	6.680	< .001	0.543	0.995
Creativity =~							
Crea_1	0.607	0.448	0.050	8.990	< .001	0.351	0.546
Crea_2	0.752	0.634	0.049	12.922	< .001	0.538	0.730
Crea_3	0.692	0.595	0.050	11.910	< .001	0.497	0.693
Crea_4	0.530	0.486	0.068	7.172	< .001	0.353	0.618
Crea_5	0.585	0.504	0.063	8.049	< .001	0.382	0.627
Crea_6	0.741	0.611	0.055	11.149	< .001	0.503	0.718
Crea_7	0.772	0.616	0.050	12.267	< .001	0.517	0.714
Crea_8	0.675	0.525	0.057	9.274	< .001	0.414	0.636
Crea_9	0.720	0.592	0.050	11.850	< .001	0.494	0.690

Note: β = standardized coefficient; *b* = unstandardized coefficient; SE = standard error; ULCI = upper limit confidence interval; LLCI = lower limit confidence interval.

Table D.3.
Factor Loadings of the 7Cs Model of Positive Youth Development Using the Youth Sample from Malaysia (n = 280)

Items	*β*	*b*	SE	z-value	*p*-value	95% ULCI	95% LLCI
Competence =~							
Comp_1	0.492	0.554	0.075	7.380	< .001	0.407	0.701
Comp_2	0.640	0.552	0.054	10.304	< .001	0.447	0.656
Comp_3	0.531	0.671	0.080	8.420	< .001	0.515	0.827
Confidence =~							
Conf_1	0.813	0.905	0.059	15.243	< .001	0.789	1.022
Conf_2	0.741	0.822	0.066	12.395	< .001	0.692	0.952
Conf_3	0.822	0.872	0.057	15.327	< .001	0.760	0.983
Character =~							
Char_1	0.467	0.516	0.073	7.080	< .001	0.373	0.658
Char_2	0.396	0.342	0.054	6.327	< .001	0.236	0.448
Char_3	0.679	0.513	0.038	13.535	< .001	0.439	0.587
Char_4	0.572	0.471	0.048	9.857	< .001	0.378	0.565
Caring =~							
Care_1	0.433	0.511	0.086	5.968	< .001	0.343	0.679
Care_2	0.647	0.452	0.049	9.134	< .001	0.355	0.549
Care_3	0.696	0.425	0.050	8.474	< .001	0.327	0.524
Connection =~							
Conn_1	0.576	0.628	0.065	9.725	< .001	0.501	0.754
Conn_2	0.753	0.791	0.053	14.833	< .001	0.687	0.896
Conn_3	0.828	1.024	0.059	17.232	< .001	0.908	1.141
Conn_4	0.466	0.503	0.068	7.444	< .001	0.371	0.636
Contribution =~							
Cont_1	0.765	0.935	0.089	10.515	< .001	0.760	1.109
Cont_2	0.653	0.781	0.080	9.713	< .001	0.624	0.939

Cont_3	0.439	0.527	0.079	6.694	< .001	0.373	0.681
Cont_4	0.689	0.826	0.076	10.834	< .001	0.677	0.976
Cont_5	0.619	0.904	0.108	8.352	< .001	0.692	1.117
Cont_6	0.713	1.064	0.094	11.282	< .001	0.879	1.249
Creativity =~							
Crea_1	0.789	0.770	0.053	14.545	< .001	0.667	0.874
Crea_2	0.834	0.827	0.049	16.797	< .001	0.730	0.923
Crea_3	0.871	0.859	0.046	18.575	< .001	0.768	0.950
Crea_4	0.860	0.902	0.046	19.790	< .001	0.813	0.992
Crea_5	0.826	0.829	0.055	15.087	< .001	0.722	0.937
Crea_6	0.840	0.797	0.047	16.960	< .001	0.705	0.889
Crea_7	0.827	0.822	0.047	17.415	< .001	0.730	0.915
Crea_8	0.878	0.853	0.044	19.228	< .001	0.766	0.940
Crea_9	0.852	0.854	0.048	17.726	< .001	0.760	0.948

Note: β = standardized coefficient; b = unstandardized coefficient; SE = standard error; ULCI = upper limit confidence interval; LLCI = lower limit confidence interval.

Table D.4.
Factor Loadings of the 7Cs Model of Positive Youth Development Using the Youth Sample from the Philippines (n = 479)

Items	*β*	*b*	SE	z-value	*p*-value	95% ULCI	95% LLCI
Competence =~							
Comp_1	0.632	0.675	0.053	12.744	< .001	0.572	0.779
Comp_2	0.615	0.516	0.047	11.075	< .001	0.425	0.608
Comp_3	0.455	0.525	0.057	9.146	< .001	0.412	0.637
Confidence =~							
Conf_1	0.793	0.863	0.053	16.197	< .001	0.759	0.968
Conf_2	0.659	0.720	0.058	12.445	< .001	0.606	0.833
Conf_3	0.876	0.861	0.055	15.512	< .001	0.752	0.969
Character =~							
Char_1	0.442	0.346	0.065	5.312	< .001	0.219	0.574
Char_2	0.598	0.399	0.056	7.166	< .001	0.290	0.508
Char_3	0.610	0.425	0.053	8.088	< .001	0.322	0.528
Char_4	0.654	0.524	0.057	9.179	< .001	0.412	0.636
Caring =~							
Care_1	0.943	0.708	0.046	15.363	< .001	0.617	0.798
Care_2	0.744	0.567	0.051	11.081	< .001	0.467	0.667
Care_3	0.798	0.587	0.052	11.263	< .001	0.485	0.689
Connection =~							
Conn_1	0.635	0.639	0.052	12.383	< .001	0.538	0.740
Conn_2	0.703	0.705	0.052	13.591	< .001	0.603	0.806
Conn_3	0.720	0.760	0.048	15.700	< .001	0.665	0.855
Conn_4	0.432	0.363	0.058	4.545	< .001	0.250	0.477
Contribution =~							
Cont_1	0.697	0.834	0.066	12.687	< .001	0.705	0.963
Cont_2	0.558	0.708	0.073	9.748	< .001	0.566	0.850

Cont_3	0.435	0.516	0.069	7.429	< .001	0.380	0.652
Cont_4	0.672	0.887	0.063	14.067	< .001	0.763	1.010
Cont_5	0.510	0.780	0.082	9.491	< .001	0.619	0.941
Cont_6	0.545	0.849	0.081	10.451	< .001	0.689	1.008
Creativity =~							
Crea_1	0.628	0.550	0.044	12.438	< .001	0.463	0.636
Crea_2	0.712	0.633	0.043	14.551	< .001	0.548	0.718
Crea_3	0.794	0.703	0.040	17.670	< .001	0.625	0.781
Crea_4	0.621	0.561	0.043	13.188	< .001	0.478	0.644
Crea_5	0.773	0.633	0.037	16.951	< .001	0.560	0.706
Crea_6	0.786	0.668	0.037	17.822	< .001	0.594	0.741
Crea_7	0.781	0.657	0.039	16.728	< .001	0.580	0.734
Crea_8	0.743	0.616	0.040	15.406	< .001	0.538	0.695
Crea_9	0.757	0.643	0.039	16.334	< .001	0.566	0.721

Note: β = standardized coefficient; *b* = unstandardized coefficient; SE = standard error; ULCI = upper limit confidence interval; LLCI = lower limit confidence interval.

Table D.5.
Factor Loadings of the 7Cs Model of Positive Youth Development Using the Youth Sample from Singapore (n = 247)

Items	*β*	*b*	SE	z-value	*p*-value	95% ULCI	95% LLCI
Competence =~							
Comp_1	0.575	0.537	0.084	6.360	< .001	0.372	0.703
Comp_2	0.597	0.542	0.073	7.456	< .001	0.400	0.684
Comp_3	0.463	0.488	0.089	4.342	< .001	0.213	0.563
Confidence =~							
Conf_1	0.816	0.857	0.060	14.344	< .001	0.740	0.974
Conf_2	0.674	0.669	0.068	9.889	< .001	0.536	0.801
Conf_3	0.846	0.840	0.065	12.850	< .001	0.712	0.968
Character =~							
Char_1	0.441	0.444	0.098	1.468	< .001	0.348	0.536
Char_2	0.705	0.592	0.075	7.847	< .001	0.444	0.740
Char_3	0.520	0.300	0.056	5.378	< .001	0.191	0.409
Char_4	0.676	0.542	0.067	8.051	< .001	0.410	0.674
Caring =~							
Care_1	0.855	0.626	0.058	10.727	< .001	0.512	0.741
Care_2	0.690	0.463	0.061	7.540	< .001	0.343	0.583
Care_3	0.790	0.517	0.057	9.098	< .001	0.406	0.628
Connection =~							
Conn_1	0.550	0.477	0.058	8.196	< .001	0.363	0.591
Conn_2	0.636	0.557	0.076	7.358	< .001	0.409	0.706
Conn_3	0.726	0.683	0.069	9.939	< .001	0.548	0.817
Conn_4	0.438	0.329	0.074	4.459	< .001	0.184	0.474
Contribution =~							
Cont_1	0.489	0.556	0.098	5.649	< .001	0.363	0.748
Cont_2	0.612	0.622	0.084	7.416	< .001	0.457	0.786

Cont_3	0.444	0.500	0.102	4.894	< .001	0.300	0.700
Cont_4	0.621	0.708	0.095	7.431	< .001	0.521	0.895
Cont_5	0.578	0.860	0.112	7.675	< .001	0.641	1.080
Cont_6	0.429	0.632	0.127	4.995	< .001	0.384	0.880
Creativity =~							
Crea_1	0.724	0.605	0.048	12.724	< .001	0.512	0.698
Crea_2	0.836	0.779	0.049	15.829	< .001	0.682	0.875
Crea_3	0.810	0.748	0.054	13.833	< .001	0.642	0.854
Crea_4	0.660	0.657	0.061	10.704	< .001	0.537	0.778
Crea_5	0.835	0.791	0.047	16.852	< .001	0.699	0.883
Crea_6	0.868	0.788	0.046	17.278	< .001	0.699	0.878
Crea_7	0.723	0.643	0.055	11.785	< .001	0.536	0.750
Crea_8	0.818	0.759	0.045	16.780	< .001	0.671	0.848
Crea_9	0.795	0.727	0.049	14.738	< .001	0.630	0.824

Note: β = standardized coefficient; b = unstandardized coefficient; SE = standard error; ULCI = upper limit confidence interval; LLCI = lower limit confidence interval.

Table D.6.
Factor Loadings of the 7Cs Model of Positive Youth Development Using the Youth Sample from Thailand (n = 486)

Items	*β*	*b*	SE	z-value	*p*-value	95% ULCI	95% LLCI
Competence =~							
Comp_1	0.565	0.561	0.052	10.714	< .001	0.459	0.664
Comp_2	0.802	0.657	0.052	12.761	< .001	0.556	0.758
Comp_3	0.424	0.436	0.067	3.514	< .001	0.304	0.567
Confidence =~							
Conf_1	0.616	0.603	0.048	12.659	< .001	0.509	0.696
Conf_2	0.820	0.767	0.036	21.397	< .001	0.697	0.837
Conf_3	0.875	0.838	0.035	24.010	< .001	0.770	0.906
Character =~							
Char_1	0.479	0.494	0.050	7.897	< .001	0.296	0.591
Char_2	0.717	0.623	0.036	17.303	< .001	0.552	0.693
Char_3	0.721	0.612	0.033	18.778	< .001	0.548	0.675
Char_4	0.619	0.610	0.041	15.039	< .001	0.530	0.689
Caring =~							
Care_1	0.878	0.762	0.037	20.332	< .001	0.688	0.835
Care_2	0.750	0.641	0.036	17.614	< .001	0.570	0.713
Care_3	0.877	0.744	0.031	23.626	< .001	0.682	0.805
Connection =~							
Conn_1	0.724	0.724	0.040	18.088	< .001	0.646	0.803
Conn_2	0.748	0.752	0.045	16.853	< .001	0.665	0.840
Conn_3	0.512	0.591	0.051	11.646	< .001	0.492	0.691
Conn_4	0.565	0.465	0.042	10.954	< .001	0.382	0.548
Contribution =~							
Cont_1	0.519	0.671	0.070	9.525	< .001	0.533	0.810
Cont_2	0.679	0.832	0.057	14.539	< .001	0.720	0.944

Cont_3	0.746	0.963	0.063	15.236	< .001	0.839	1.087
Cont_4	0.802	0.977	0.055	17.854	< .001	0.870	1.084
Cont_5	0.743	1.017	0.058	17.414	< .001	0.902	1.131
Cont_6	0.565	0.759	0.069	10.989	< .001	0.623	0.894
Creativity =~							
Crea_1	0.739	0.593	0.032	18.579	< .001	0.530	0.655
Crea_2	0.800	0.698	0.034	20.567	< .001	0.631	0.764
Crea_3	0.825	0.718	0.032	22.697	< .001	0.656	0.780
Crea_4	0.765	0.646	0.032	20.170	< .001	0.583	0.708
Crea_5	0.775	0.726	0.033	21.818	< .001	0.660	0.791
Crea_6	0.768	0.713	0.037	19.218	< .001	0.640	0.785
Crea_7	0.734	0.631	0.034	18.580	< .001	0.564	0.697
Crea_8	0.836	0.789	0.034	23.177	< .001	0.722	0.855
Crea_9	0.852	0.835	0.034	24.733	< .001	0.769	0.902

Note: β = standardized coefficient; *b* = unstandardized coefficient; SE = standard error; ULCI = upper limit confidence interval; LLCI = lower limit confidence interval.

Table D.7.
Factor Loadings of the 7Cs Model of Positive Youth Development Using the Youth Sample from Vietnam (n = 248)

Items	*β*	*b*	SE	z-value	*p*-value	95% ULCI	95% LLCI
Competence =~							
Comp_1	0.669	0.648	0.065	9.952	< .001	0.520	0.775
Comp_2	0.712	0.576	0.051	11.343	< .001	0.477	0.676
Comp_3	0.403	0.412	0.076	5.382	< .001	0.262	0.562
Confidence =~							
Conf_1	0.763	0.675	0.051	13.297	< .001	0.575	0.774
Conf_2	0.572	0.531	0.063	8.491	< .001	0.408	0.653
Conf_3	0.819	0.679	0.055	12.327	< .001	0.571	0.787
Character =~							
Char_1	0.489	0.443	0.064	5.330	< .001	0.217	0.569
Char_2	0.675	0.582	0.068	8.546	< .001	0.448	0.715
Char_3	0.581	0.457	0.066	6.902	< .001	0.327	0.587
Char_4	0.421	0.426	0.087	1.446	< .001	0.298	0.445
Caring =~							
Care_1	0.871	0.687	0.063	10.981	< .001	0.564	0.809
Care_2	0.606	0.459	0.063	7.310	< .001	0.336	0.582
Care_3	0.840	0.655	0.046	14.236	< .001	0.565	0.746
Connection =~							
Conn_1	0.686	0.570	0.056	10.167	< .001	0.460	0.680
Conn_2	0.698	0.617	0.056	11.046	< .001	0.508	0.727
Conn_3	0.623	0.584	0.060	9.674	< .001	0.465	0.702
Conn_4	0.545	0.436	0.062	7.064	< .001	0.315	0.557
Contribution =~							
Cont_1	0.818	1.057	0.073	14.582	< .001	0.915	1.200
Cont_2	0.775	0.822	0.063	13.077	< .001	0.699	0.945

Cont_3	0.637	0.736	0.081	9.130	< .001	0.578	0.894
Cont_4	0.631	0.767	0.080	9.632	< .001	0.611	0.923
Cont_5	0.615	0.867	0.086	10.114	< .001	0.699	1.035
Cont_6	0.619	0.870	0.084	10.354	< .001	0.705	1.035
Creativity =~							
Crea_1	0.670	0.522	0.059	8.836	< .001	0.406	0.637
Crea_2	0.806	0.640	0.045	14.353	< .001	0.553	0.727
Crea_3	0.833	0.677	0.047	14.384	< .001	0.585	0.770
Crea_4	0.798	0.634	0.045	14.042	< .001	0.545	0.722
Crea_5	0.703	0.575	0.052	11.117	< .001	0.474	0.676
Crea_6	0.698	0.583	0.051	11.396	< .001	0.482	0.683
Crea_7	0.754	0.573	0.045	12.871	< .001	0.486	0.661
Crea_8	0.828	0.670	0.055	12.136	< .001	0.562	0.778
Crea_9	0.805	0.633	0.056	11.408	< .001	0.524	0.742

Note: β = standardized coefficient; b = unstandardized coefficient; SE = standard error; ULCI = upper limit confidence interval; LLCI = lower limit confidence interval.

Supplementary Material E

Table E.1.
Factor Loadings of the Measurement Model of Hope Using the Overall Youth Sample (n = 1,967)

Items	*β*	*b*	SE	z-value	*p*-value	95% ULCI	95% LLCI
Hope =~							
Hope_1	0.794	0.794	0.009	87.360	< .001	0.776	0.812
Hope_2	0.757	0.757	0.010	75.633	< .001	0.738	0.777
Hope_3	0.683	0.683	0.012	58.148	< .001	0.660	0.706
Hope_4	0.757	0.757	0.010	75.774	< .001	0.738	0.777
Hope_5	0.735	0.735	0.011	68.351	< .001	0.714	0.757
Hope_6	0.529	0.529	0.016	33.962	< .001	0.498	0.559
Hope_7	0.726	0.726	0.011	63.633	< .001	0.704	0.749
Hope_8	0.743	0.743	0.010	72.940	< .001	0.723	0.763
Hope_9	0.726	0.726	0.011	66.199	< .001	0.705	0.748
Hope_10	0.767	0.767	0.010	80.004	< .001	0.748	0.786
Hope_11	0.844	0.844	0.007	113.407	< .001	0.830	0.859
Hope_12	0.848	0.848	0.007	115.407	< .001	0.833	0.862

Note: *β* = standardized coefficient; *b* = unstandardized coefficient; SE = standard error; ULCI = upper limit confidence interval; LLCI = lower limit confidence interval.

Table E.2.
Factor Loadings of the Measurement Model of Hope Using the Youth Sample from Indonesia (n = 227)

Items	*β*	*b*	SE	z-value	*p*-value	95% ULCI	95% LLCI
Hope =~							
Hope_1	0.755	0.755	0.029	26.165	< .001	0.699	0.812
Hope_2	0.606	0.606	0.036	16.672	< .001	0.535	0.678
Hope_3	0.562	0.562	0.043	12.934	< .001	0.477	0.647
Hope_4	0.674	0.674	0.034	19.700	< .001	0.607	0.742
Hope_5	0.700	0.700	0.034	20.411	< .001	0.633	0.767
Hope_6	0.605	0.605	0.040	15.236	< .001	0.527	0.682
Hope_7	0.682	0.682	0.040	17.157	< .001	0.604	0.760
Hope_8	0.614	0.614	0.040	15.243	< .001	0.535	0.693
Hope_9	0.672	0.672	0.036	18.600	< .001	0.601	0.743
Hope_10	0.713	0.713	0.031	22.652	< .001	0.651	0.775
Hope_11	0.815	0.815	0.026	30.899	< .001	0.763	0.867
Hope_12	0.752	0.752	0.029	25.800	< .001	0.695	0.809

Note: *β* = standardized coefficient; *b* = unstandardized coefficient; SE = standard error; ULCI = upper limit confidence interval; LLCI = lower limit confidence interval.

Table E.3.
Factor Loadings of the Measurement Model of Hope Using the Youth Sample from Malaysia (n = 280)

Items	*β*	*b*	SE	z-value	*p*-value	95% ULCI	95% LLCI
Hope =~							
Hope_1	0.831	0.831	0.020	41.070	< .001	0.791	0.870
Hope_2	0.825	0.825	0.019	42.491	< .001	0.787	0.863
Hope_3	0.756	0.756	0.026	28.684	< .001	0.704	0.807
Hope_4	0.775	0.775	0.023	33.216	< .001	0.729	0.821
Hope_5	0.787	0.787	0.024	32.460	< .001	0.739	0.834
Hope_6	0.561	0.561	0.037	15.007	< .001	0.488	0.634
Hope_7	0.770	0.770	0.025	30.718	< .001	0.721	0.819
Hope_8	0.770	0.770	0.025	31.152	< .001	0.721	0.818
Hope_9	0.688	0.688	0.031	22.041	< .001	0.627	0.749
Hope_10	0.843	0.843	0.020	41.880	< .001	0.804	0.883
Hope_11	0.874	0.874	0.017	50.711	< .001	0.840	0.908
Hope_12	0.912	0.912	0.014	67.455	< .001	0.885	0.938

Note: *β* = standardized coefficient; *b* = unstandardized coefficient; SE = standard error; ULCI = upper limit confidence interval; LLCI = lower limit confidence interval.

Table E.4.
Factor Loadings of Measurement Model of Hope Using the Youth Sample from the Philippines (n = 479)

Items	*β*	*b*	SE	z-value	*p*-value	95% ULCI	95% LLCI
Hope =~							
Hope_1	0.781	0.781	0.020	38.374	< .001	0.741	0.821
Hope_2	0.759	0.759	0.022	34.568	< .001	0.716	0.802
Hope_3	0.701	0.701	0.023	31.039	< .001	0.657	0.745
Hope_4	0.735	0.735	0.022	33.058	< .001	0.692	0.779
Hope_5	0.757	0.757	0.021	35.844	< .001	0.716	0.798
Hope_6	0.508	0.508	0.033	15.308	< .001	0.443	0.574
Hope_7	0.723	0.723	0.023	30.994	< .001	0.677	0.768
Hope_8	0.782	0.782	0.019	42.096	< .001	0.746	0.819
Hope_9	0.767	0.767	0.021	36.604	< .001	0.726	0.808
Hope_10	0.755	0.755	0.021	36.025	< .001	0.714	0.796
Hope_11	0.870	0.870	0.014	62.518	< .001	0.842	0.897
Hope_12	0.822	0.822	0.017	48.631	< .001	0.789	0.855

Note: *β* = standardized coefficient; *b* = unstandardized coefficient; SE = standard error; ULCI = upper limit confidence interval; LLCI = lower limit confidence interval.

Table E.5.

Factor Loadings of the Measurement Model of Hope Using the Youth Sample from Singapore (n = 247)

Items	*β*	*b*	SE	z-value	*p*-value	95% ULCI	95% LLCI
Hope =~							
Hope_1	0.856	0.856	0.022	39.298	< .001	0.814	0.899
Hope_2	0.763	0.763	0.030	25.415	< .001	0.704	0.822
Hope_3	0.654	0.654	0.036	18.300	< .001	0.584	0.724
Hope_4	0.753	0.753	0.032	23.250	< .001	0.690	0.816
Hope_5	0.586	0.586	0.044	13.435	< .001	0.500	0.671
Hope_6	0.556	0.556	0.043	12.990	< .001	0.472	0.640
Hope_7	0.620	0.620	0.042	14.667	< .001	0.537	0.703
Hope_8	0.762	0.762	0.030	25.372	< .001	0.703	0.821
Hope_9	0.712	0.712	0.032	22.370	< .001	0.650	0.775
Hope_10	0.788	0.788	0.027	29.586	< .001	0.736	0.840
Hope_11	0.780	0.780	0.029	27.128	< .001	0.723	0.836
Hope_12	0.900	0.900	0.017	54.036	< .001	0.867	0.932

Note: *β* = standardized coefficient; *b* = unstandardized coefficient; SE = standard error; ULCI = upper limit confidence interval; LLCI = lower limit confidence interval.

Table E.6.

Factor Loadings of the Measurement Model of Hope Using the Youth Sample from Thailand (n = 486)

Items	*β*	*b*	SE	z-value	*p*-value	95% ULCI	95% LLCI
Hope =~							
Hope_1	0.782	0.782	0.017	45.136	< .001	0.748	0.815
Hope_2	0.758	0.758	0.020	38.217	< .001	0.719	0.797
Hope_3	0.745	0.745	0.020	37.297	< .001	0.706	0.784
Hope_4	0.770	0.770	0.020	37.906	< .001	0.730	0.810
Hope_5	0.830	0.830	0.015	54.046	< .001	0.800	0.860
Hope_6	0.507	0.507	0.031	16.602	< .001	0.447	0.567
Hope_7	0.772	0.772	0.019	40.601	< .001	0.735	0.809
Hope_8	0.747	0.747	0.020	38.089	< .001	0.708	0.785
Hope_9	0.701	0.701	0.018	37.952	< .001	0.665	0.737
Hope_10	0.744	0.744	0.020	37.730	< .001	0.706	0.783
Hope_11	0.823	0.823	0.015	55.178	< .001	0.794	0.853
Hope_12	0.835	0.835	0.017	48.902	< .001	0.801	0.868

Note: *β* = standardized coefficient; *b* = unstandardized coefficient; SE = standard error; ULCI = upper limit confidence interval; LLCI = lower limit confidence interval.

Table E.7.

Factor Loadings of the Measurement Model of Hope Using the Youth Sample from Vietnam (n = 248)

Items	*β*	*b*	SE	z-value	*p*-value	95% ULCI	95% LLCI
Hope =~							
Hope_1	0.791	0.791	0.023	34.583	< .001	0.746	0.835
Hope_2	0.730	0.730	0.029	25.107	< .001	0.673	0.787
Hope_3	0.555	0.555	0.036	15.277	< .001	0.483	0.626
Hope_4	0.843	0.843	0.020	41.182	< .001	0.803	0.884
Hope_5	0.742	0.742	0.026	28.331	< .001	0.690	0.793
Hope_6	0.633	0.633	0.034	18.458	< .001	0.566	0.701
Hope_7	0.762	0.762	0.025	30.194	< .001	0.713	0.812
Hope_8	0.761	0.761	0.024	31.135	< .001	0.713	0.809
Hope_9	0.817	0.817	0.020	40.185	< .001	0.777	0.857
Hope_10	0.797	0.797	0.022	37.028	< .001	0.754	0.839
Hope_11	0.831	0.831	0.019	43.782	< .001	0.794	0.869
Hope_12	0.896	0.896	0.018	50.759	< .001	0.861	0.931

Note: *β* = standardized coefficient; *b* = unstandardized coefficient; SE = standard error; ULCI = upper limit confidence interval; LLCI = lower limit confidence interval.

Supplementary Material F

Table F.1.
Factor Loadings of the Measurement Model of Life Satisfaction Using the Overall Youth Sample (n = 1,967)

Items	*β*	*b*	SE	z-value	*p*-value	95% ULCI	95% LLCI
Life Satisfaction =~							
SWLS_1	0.812	0.812	0.008	96.934	< .001	0.795	0.828
SWLS_2	0.908	0.908	0.006	141.344	< .001	0.896	0.921
SWLS_3	0.845	0.845	0.008	110.307	< .001	0.830	0.860

Note: *β* = standardized coefficient; *b* = unstandardized coefficient; SE = standard error; ULCI = upper limit confidence interval; LLCI = lower limit confidence interval.

Table F.2.
Factor Loadings of the Measurement Model of Life Satisfaction Using the Youth Sample from Indonesia (n = 227)

Items	*β*	*b*	SE	z-value	*p*-value	95% ULCI	95% LLCI
Life Satisfaction =~							
SWLS_1	0.754	0.754	0.038	19.758	< .001	0.679	0.829
SWLS_2	0.898	0.898	0.027	32.886	< .001	0.844	0.951
SWLS_3	0.819	0.819	0.034	23.978	< .001	0.752	0.886

Note: *β* = standardized coefficient; *b* = unstandardized coefficient; SE = standard error; ULCI = upper limit confidence interval; LLCI = lower limit confidence interval.

Table F.3.
Factor Loadings of the Measurement Model of Life Satisfaction Using the Youth Sample from Malaysia (n = 280)

Items	*β*	*b*	SE	z-value	*p*-value	95% ULCI	95% LLCI
Life Satisfaction =~							
SWLS_1	0.676	0.676	0.029	23.160	< .001	0.618	0.733
SWLS_2	0.996	0.996	0.014	71.200	< .001	0.969	1.024
SWLS_3	0.895	0.895	0.017	52.309	< .001	0.862	0.929

Note: *β* = standardized coefficient; *b* = unstandardized coefficient; SE = standard error; ULCI = upper limit confidence interval; LLCI = lower limit confidence interval.

Table F.4.
Factor Loadings of the Measurement Model of Life Satisfaction Using the Youth Sample from the Philippines (n = 479)

Items	*β*	*b*	SE	z-value	*p*-value	95% ULCI	95% LLCI
Life Satisfaction =~							
SWLS_1	0.793	0.793	0.019	40.947	< .001	0.755	0.831
SWLS_2	0.883	0.883	0.018	48.675	< .001	0.848	0.919
SWLS_3	0.828	0.828	0.020	42.421	< .001	0.789	0.866

Note: *β* = standardized coefficient; *b* = unstandardized coefficient; SE = standard error; ULCI = upper limit confidence interval; LLCI = lower limit confidence interval.

Table F.5.
Factor Loadings of the Measurement Model of Life Satisfaction Using the Youth Sample from Singapore (n = 247)

Items	*β*	*b*	SE	z-value	*p*-value	95% ULCI	95% LLCI
Life Satisfaction =~							
SWLS_1	0.846	0.846	0.023	36.739	< .001	0.801	0.891
SWLS_2	0.854	0.854	0.022	37.959	< .001	0.810	0.898
SWLS_3	0.901	0.901	0.021	43.159	< .001	0.860	0.942

Note: *β* = standardized coefficient; *b* = unstandardized coefficient; SE = standard error; ULCI = upper limit confidence interval; LLCI = lower limit confidence interval.

Table F.6.
Factor Loadings of the Measurement Model of Life Satisfaction Using the Youth Sample from Thailand (n = 486)

Items	*β*	*b*	SE	z-value	*p*-value	95% ULCI	95% LLCI
Life Satisfaction =~							
SWLS_1	0.896	0.896	0.011	79.548	< .001	0.874	0.918
SWLS_2	0.922	0.922	0.011	83.146	< .001	0.900	0.944
SWLS_3	0.800	0.800	0.017	47.219	< .001	0.767	0.833

Note: *β* = standardized coefficient; *b* = unstandardized coefficient; SE = standard error; ULCI = upper limit confidence interval; LLCI = lower limit confidence interval.

Table F.7.
Factor Loadings of the Measurement Model of Life Satisfaction Using the Youth Sample from Vietnam (n = 248)

Items	*β*	*b*	SE	z-value	*p*-value	95% ULCI	95% LLCI
Life Satisfaction =~							
SWLS_1	0.823	0.823	0.019	42.533	< .001	0.785	0.861
SWLS_2	0.870	0.870	0.019	46.533	< .001	0.834	0.907
SWLS_3	0.922	0.922	0.017	55.693	< .001	0.889	0.954

Note: *β* = standardized coefficient; *b* = unstandardized coefficient; SE = standard error; ULCI = upper limit confidence interval; LLCI = lower limit confidence interval.

Supplementary Material G

Table G.1.
Factor Loadings of the Measurement Model (Step 1) of the 7Cs of Positive Youth Development, Hope, and Life Satisfaction Using the Overall Youth Sample (n = 1,967)

Items	*β*	*b*	SE	z-value	*p*-value	95% ULCI	95% LLCI
Competence =~							
Comp_1	0.640	0.640	0.018	35.169	< .001	0.605	0.676
Comp_2	0.751	0.751	0.017	44.766	< .001	0.718	0.784
Comp_3	0.408	0.408	0.023	17.995	< .001	0.364	0.453
Confidence =~							
Conf_1	0.782	0.782	0.013	58.665	< .001	0.756	0.808
Conf_2	0.738	0.738	0.013	58.096	< .001	0.713	0.763
Conf_3	0.918	0.918	0.010	94.356	< .001	0.899	0.937
Character =~							
Char_1	0.425	0.425	0.022	19.775	< .001	0.383	0.468
Char_2	0.726	0.726	0.015	49.356	< .001	0.697	0.754
Char_3	0.769	0.769	0.014	54.725	< .001	0.741	0.796
Char_4	0.587	0.587	0.017	34.266	< .001	0.553	0.620
Caring =~							
Care_1	0.752	0.752	0.015	49.394	< .001	0.722	0.782
Care_2	0.866	0.866	0.011	82.266	< .001	0.845	0.886
Care_3	0.957	0.957	0.010	99.294	< .001	0.939	0.976
Connection =~							
Conn_1	0.683	0.683	0.015	46.747	< .001	0.654	0.711
Conn_2	0.769	0.769	0.013	59.632	< .001	0.744	0.795
Conn_3	0.663	0.663	0.014	47.389	< .001	0.636	0.691
Conn_4	0.564	0.564	0.019	29.560	< .001	0.526	0.601
Contribution =~							

Cont_1	0.687	0.687	0.023	30.544	< .001	0.643	0.732
Cont_2	0.680	0.680	0.019	35.296	< .001	0.642	0.718
Cont_3	0.695	0.695	0.024	28.890	< .001	0.648	0.742
Cont_4	0.702	0.702	0.019	37.706	< .001	0.665	0.738
Cont_5	0.662	0.662	0.021	31.651	< .001	0.621	0.703
Cont_6	0.652	0.652	0.023	28.570	< .001	0.607	0.696
Creativity =~							
Crea_1	0.741	0.741	0.012	63.417	< .001	0.718	0.764
Crea_2	0.832	0.832	0.008	101.884	< .001	0.816	0.848
Crea_3	0.849	0.849	0.007	118.844	< .001	0.835	0.863
Crea_4	0.767	0.767	0.010	78.958	< .001	0.748	0.786
Crea_5	0.821	0.821	0.008	97.238	< .001	0.805	0.838
Crea_6	0.819	0.819	0.008	104.550	< .001	0.804	0.834
Crea_7	0.803	0.803	0.009	91.925	< .001	0.786	0.820
Crea_8	0.916	0.916	0.005	193.826	< .001	0.907	0.925
Crea_9	0.921	0.921	0.005	194.694	< .001	0.912	0.930
Hope =~							
Hope_1	0.794	0.794	0.010	79.974	< .001	0.774	0.813
Hope_2	0.746	0.746	0.011	67.944	< .001	0.724	0.767
Hope_3	0.690	0.690	0.012	56.054	< .001	0.666	0.714
Hope_4	0.765	0.765	0.011	70.828	< .001	0.744	0.786
Hope_5	0.734	0.734	0.012	63.314	< .001	0.711	0.757
Hope_6	0.526	0.526	0.016	32.022	< .001	0.494	0.559
Hope_7	0.726	0.726	0.013	57.131	< .001	0.701	0.751
Hope_8	0.747	0.747	0.011	68.963	< .001	0.726	0.768
Hope_9	0.737	0.737	0.012	63.564	< .001	0.715	0.760
Hope_10	0.764	0.764	0.010	73.034	< .001	0.744	0.785
Hope_11	0.840	0.840	0.008	100.000	< .001	0.823	0.856

Hope_12	0.845	0.845	0.008	105.307	< .001	0.829	0.861
Life Satisfaction =~							
SWLS_1	0.844	0.844	0.011	77.075	< .001	0.823	0.866
SWLS_2	0.848	0.848	0.009	90.145	< .001	0.829	0.866
SWLS_3	0.884	0.884	0.009	95.258	< .001	0.866	0.903

Note: β = standardized coefficient; *b* = unstandardized coefficient; SE = standard error; ULCI = upper limit confidence interval; LLCI = lower limit confidence interval.

Table G.2.
Factor Loadings of the Measurement Model (Step 1) of the 7Cs of Positive Youth Development, Hope, and Life Satisfaction Using the Youth Sample from Indonesia (n = 227)

Items	*β*	*b*	SE	z-value	*p*-value	95% ULCI	95% LLCI
Competence =~							
Comp_1	0.763	0.763	0.042	18.157	< .001	0.681	0.846
Comp_2	0.745	0.745	0.044	17.062	< .001	0.660	0.831
Comp_3	0.474	0.474	0.073	5.112	< .001	0.330	0.517
Confidence =~							
Conf_1	0.851	0.851	0.026	32.950	< .001	0.801	0.902
Conf_2	0.838	0.838	0.023	37.010	< .001	0.794	0.882
Conf_3	0.921	0.921	0.019	47.888	< .001	0.883	0.958
Character =~							
Char_1	0.487	0.487	0.063	6.182	< .001	0.364	0.509
Char_2	0.571	0.571	0.046	12.437	< .001	0.481	0.661
Char_3	0.706	0.706	0.048	14.656	< .001	0.611	0.800
Char_4	0.626	0.626	0.041	15.268	< .001	0.545	0.706
Caring =~							
Care_1	0.809	0.809	0.037	21.647	< .001	0.736	0.883
Care_2	0.852	0.852	0.032	26.844	< .001	0.789	0.914
Care_3	0.938	0.938	0.030	31.785	< .001	0.881	0.996
Connection =~							
Conn_1	0.654	0.654	0.044	14.826	< .001	0.568	0.741
Conn_2	0.708	0.708	0.037	19.367	< .001	0.637	0.780
Conn_3	0.731	0.731	0.037	19.793	< .001	0.659	0.803
Conn_4	0.587	0.587	0.052	11.193	< .001	0.484	0.690
Contribution =~							
Cont_1	0.518	0.518	0.076	6.828	< .001	0.369	0.667

Cont_2	0.561	0.561	0.071	7.935	< .001	0.423	0.700
Cont_3	0.715	0.715	0.060	12.022	< .001	0.599	0.832
Cont_4	0.741	0.741	0.053	13.848	< .001	0.636	0.845
Cont_5	0.537	0.537	0.066	8.098	< .001	0.407	0.666
Cont_6	0.625	0.625	0.070	8.911	< .001	0.487	0.762
Creativity =~							
Crea_1	0.739	0.739	0.044	16.922	< .001	0.653	0.824
Crea_2	0.765	0.765	0.032	24.171	< .001	0.703	0.827
Crea_3	0.760	0.760	0.033	22.831	< .001	0.695	0.825
Crea_4	0.589	0.589	0.043	13.763	< .001	0.505	0.673
Crea_5	0.674	0.674	0.039	17.077	< .001	0.596	0.751
Crea_6	0.792	0.792	0.032	25.013	< .001	0.730	0.855
Crea_7	0.841	0.841	0.023	36.475	< .001	0.796	0.886
Crea_8	0.766	0.766	0.028	27.119	< .001	0.710	0.821
Crea_9	0.847	0.847	0.023	36.968	< .001	0.802	0.891
Hope =~							
Hope_1	0.749	0.749	0.033	22.382	< .001	0.684	0.815
Hope_2	0.560	0.560	0.043	13.008	< .001	0.476	0.645
Hope_3	0.639	0.639	0.043	14.887	< .001	0.555	0.723
Hope_4	0.691	0.691	0.038	18.340	< .001	0.617	0.765
Hope_5	0.670	0.670	0.039	17.112	< .001	0.593	0.747
Hope_6	0.632	0.632	0.040	15.790	< .001	0.553	0.710
Hope_7	0.667	0.667	0.048	13.865	< .001	0.573	0.762
Hope_8	0.618	0.618	0.042	14.813	< .001	0.536	0.699
Hope_9	0.678	0.678	0.036	18.683	< .001	0.607	0.749
Hope_10	0.732	0.732	0.035	21.073	< .001	0.664	0.800
Hope_11	0.809	0.809	0.030	27.033	< .001	0.750	0.868
Hope_12	0.720	0.720	0.035	20.740	< .001	0.652	0.788

Life Satisfaction =~							
SWLS_1	0.785	0.785	0.040	19.806	< .001	0.708	0.863
SWLS_2	0.843	0.843	0.032	26.744	< .001	0.782	0.905
SWLS_3	0.854	0.854	0.034	25.228	< .001	0.788	0.921

Note: β = standardized coefficient; b = unstandardized coefficient; SE = standard error; ULCI = upper limit confidence interval; LLCI = lower limit confidence interval.

Table G.3.
Factor Loadings of the Measurement Model (Step 1) of the 7Cs of Positive Youth Development, Hope, and Life Satisfaction Using the Youth Sample from Malaysia (n = 280)

Items	*β*	*b*	SE	z-value	*p*-value	95% ULCI	95% LLCI
Competence =~							
Comp_1	0.574	0.574	0.049	11.673	< .001	0.478	0.671
Comp_2	0.728	0.728	0.044	16.375	< .001	0.641	0.816
Comp_3	0.525	0.525	0.048	11.053	< .001	0.432	0.619
Confidence =~							
Conf_1	0.853	0.853	0.026	33.225	< .001	0.802	0.903
Conf_2	0.762	0.762	0.031	24.405	< .001	0.701	0.823
Conf_3	0.901	0.901	0.020	46.063	< .001	0.863	0.940
Character =~							
Char_1	0.570	0.570	0.055	10.284	< .001	0.461	0.678
Char_2	0.498	0.498	0.056	8.948	< .001	0.389	0.607
Char_3	0.845	0.845	0.033	25.909	< .001	0.781	0.909
Char_4	0.647	0.647	0.035	18.330	< .001	0.578	0.717
Caring =~							
Care_1	0.461	0.461	0.064	7.187	< .001	0.335	0.587
Care_2	0.861	0.861	0.047	18.282	< .001	0.769	0.953
Care_3	0.901	0.901	0.044	20.662	< .001	0.815	0.986
Connection =~							
Conn_1	0.708	0.708	0.037	19.300	< .001	0.636	0.780
Conn_2	0.799	0.799	0.027	29.458	< .001	0.746	0.852
Conn_3	0.791	0.791	0.028	28.431	< .001	0.736	0.845
Conn_4	0.564	0.564	0.043	13.236	< .001	0.481	0.648
Contribution =~							
Cont_1	0.888	0.888	0.062	14.240	< .001	0.766	1.010

Cont_2	0.668	0.668	0.050	13.387	< .001	0.570	0.766
Cont_3	0.491	0.491	0.072	6.848	< .001	0.351	0.632
Cont_4	0.722	0.722	0.046	15.636	< .001	0.631	0.812
Cont_5	0.748	0.748	0.052	14.377	< .001	0.646	0.850
Cont_6	0.801	0.801	0.045	17.946	< .001	0.714	0.889
Creativity =~							
Crea_1	0.835	0.835	0.021	39.753	< .001	0.794	0.876
Crea_2	0.873	0.873	0.018	49.105	< .001	0.839	0.908
Crea_3	0.908	0.908	0.012	77.802	< .001	0.885	0.930
Crea_4	0.903	0.903	0.013	68.611	< .001	0.877	0.928
Crea_5	0.871	0.871	0.016	54.994	< .001	0.840	0.902
Crea_6	0.865	0.865	0.017	49.608	< .001	0.831	0.899
Crea_7	0.888	0.888	0.013	68.205	< .001	0.862	0.913
Crea_8	0.961	0.961	0.007	139.853	< .001	0.948	0.975
Crea_9	0.934	0.934	0.010	95.896	< .001	0.915	0.954
Hope =~							
Hope_1	0.822	0.822	0.023	35.735	< .001	0.777	0.867
Hope_2	0.808	0.808	0.024	34.146	< .001	0.761	0.854
Hope_3	0.752	0.752	0.029	25.978	< .001	0.695	0.809
Hope_4	0.805	0.805	0.023	34.389	< .001	0.759	0.851
Hope_5	0.791	0.791	0.025	31.749	< .001	0.743	0.840
Hope_6	0.551	0.551	0.040	13.624	< .001	0.472	0.631
Hope_7	0.780	0.780	0.027	28.976	< .001	0.727	0.833
Hope_8	0.792	0.792	0.024	33.204	< .001	0.745	0.839
Hope_9	0.683	0.683	0.033	20.985	< .001	0.619	0.747
Hope_10	0.852	0.852	0.021	40.281	< .001	0.810	0.893
Hope_11	0.855	0.855	0.021	41.299	< .001	0.814	0.896
Hope_12	0.908	0.908	0.015	59.330	< .001	0.878	0.938

Life Satisfaction =~							
SWLS_1	0.777	0.777	0.035	22.115	< .001	0.708	0.845
SWLS_2	0.923	0.923	0.015	59.784	< .001	0.893	0.953
SWLS_3	0.943	0.943	0.014	65.207	< .001	0.915	0.972

Note: β = standardized coefficient; *b* = unstandardized coefficient; SE = standard error; ULCI = upper limit confidence interval; LLCI = lower limit confidence interval.

Table G.4.
Factor Loadings of the Measurement Model (Step 1) of the 7Cs of Positive Youth Development, Hope, and Life Satisfaction Using the Youth Sample from the Philippines (n = 479)

Items	*β*	*b*	SE	z-value	*p*-value	95% ULCI	95% LLCI
Competence =~							
Comp_1	0.669	0.669	0.040	16.925	< .001	0.591	0.746
Comp_2	0.696	0.696	0.036	19.591	< .001	0.627	0.766
Comp_3	0.484	0.484	0.042	11.580	< .001	0.402	0.566
Confidence =~							
Conf_1	0.824	0.824	0.028	29.375	< .001	0.769	0.879
Conf_2	0.685	0.685	0.030	22.607	< .001	0.626	0.745
Conf_3	0.922	0.922	0.025	37.611	< .001	0.874	0.970
Character =~							
Char_1	0.481	0.481	0.044	8.624	< .001	0.295	0.468
Char_2	0.769	0.769	0.029	26.355	< .001	0.712	0.827
Char_3	0.742	0.742	0.031	24.155	< .001	0.682	0.802
Char_4	0.707	0.707	0.034	20.740	< .001	0.640	0.774
Caring =~							
Care_1	0.889	0.889	0.021	42.392	< .001	0.848	0.930
Care_2	0.957	0.957	0.015	62.217	< .001	0.927	0.987
Care_3	0.970	0.970	0.014	71.657	< .001	0.943	0.996
Connection =~							
Conn_1	0.680	0.680	0.033	20.314	< .001	0.614	0.745
Conn_2	0.811	0.811	0.026	30.757	< .001	0.760	0.863
Conn_3	0.723	0.723	0.027	26.843	< .001	0.671	0.776
Conn_4	0.462	0.462	0.049	9.487	< .001	0.367	0.558
Contribution =~							
Cont_1	0.811	0.811	0.042	19.177	< .001	0.728	0.894

Cont_2	0.609	0.609	0.046	13.346	< .001	0.520	0.699
Cont_3	0.551	0.551	0.058	9.453	< .001	0.437	0.665
Cont_4	0.714	0.714	0.037	19.235	< .001	0.642	0.787
Cont_5	0.583	0.583	0.050	11.762	< .001	0.486	0.680
Cont_6	0.635	0.635	0.047	13.402	< .001	0.542	0.728
Creativity =~							
Crea_1	0.664	0.664	0.026	25.068	< .001	0.612	0.715
Crea_2	0.800	0.800	0.018	44.610	< .001	0.765	0.835
Crea_3	0.853	0.853	0.014	62.221	< .001	0.826	0.880
Crea_4	0.695	0.695	0.023	29.712	< .001	0.649	0.741
Crea_5	0.840	0.840	0.015	57.395	< .001	0.811	0.869
Crea_6	0.830	0.830	0.017	49.103	< .001	0.797	0.863
Crea_7	0.801	0.801	0.017	45.825	< .001	0.767	0.836
Crea_8	0.877	0.877	0.012	74.584	< .001	0.854	0.900
Crea_9	0.895	0.895	0.012	72.735	< .001	0.871	0.920
Hope =~							
Hope_1	0.793	0.793	0.022	36.770	< .001	0.751	0.836
Hope_2	0.748	0.748	0.023	32.746	< .001	0.703	0.793
Hope_3	0.704	0.704	0.023	29.958	< .001	0.658	0.750
Hope_4	0.737	0.737	0.025	30.028	< .001	0.689	0.785
Hope_5	0.745	0.745	0.023	32.596	< .001	0.700	0.790
Hope_6	0.505	0.505	0.034	14.709	< .001	0.438	0.572
Hope_7	0.708	0.708	0.025	28.039	< .001	0.659	0.758
Hope_8	0.784	0.784	0.020	39.123	< .001	0.744	0.823
Hope_9	0.784	0.784	0.021	36.775	< .001	0.742	0.826
Hope_10	0.754	0.754	0.022	35.076	< .001	0.712	0.796
Hope_11	0.867	0.867	0.015	56.739	< .001	0.837	0.897
Hope_12	0.827	0.827	0.018	46.058	< .001	0.792	0.862

Life Satisfaction =~							
SWLS_1	0.870	0.870	0.021	40.712	< .001	0.828	0.911
SWLS_2	0.785	0.785	0.022	35.930	< .001	0.742	0.828
SWLS_3	0.864	0.864	0.022	39.122	< .001	0.821	0.907

Note: β = standardized coefficient; *b* = unstandardized coefficient; SE = standard error; ULCI = upper limit confidence interval; LLCI = lower limit confidence interval.

Table G.5.
Factor Loadings of the Measurement Model (Step 1) of the 7Cs of Positive Youth Development, Hope, and Life Satisfaction Using the Youth Sample from Singapore (n = 247)

Items	*β*	*b*	SE	z-value	*p*-value	95% ULCI	95% LLCI
Competence =~							
Comp_1	0.548	0.548	0.068	8.095	< .001	0.415	0.680
Comp_2	0.701	0.701	0.061	11.488	< .001	0.581	0.821
Comp_3	0.426	0.426	0.066	6.472	< .001	0.297	0.555
Confidence =~							
Conf_1	0.904	0.904	0.024	37.030	< .001	0.856	0.952
Conf_2	0.692	0.692	0.035	19.713	< .001	0.623	0.761
Conf_3	0.873	0.873	0.023	37.919	< .001	0.828	0.918
Character =~							
Char_1	0.472	0.472	0.064	4.252	< .001	0.347	0.598
Char_2	0.737	0.737	0.041	18.157	< .001	0.657	0.817
Char_3	0.624	0.624	0.049	12.765	< .001	0.528	0.719
Char_4	0.701	0.701	0.042	16.825	< .001	0.620	0.783
Caring =~							
Care_1	0.860	0.860	0.034	24.997	< .001	0.793	0.928
Care_2	0.876	0.876	0.026	33.653	< .001	0.825	0.927
Care_3	0.881	0.881	0.025	35.749	< .001	0.832	0.929
Connection =~							
Conn_1	0.559	0.559	0.049	11.304	< .001	0.462	0.656
Conn_2	0.731	0.731	0.036	20.511	< .001	0.661	0.801
Conn_3	0.767	0.767	0.033	23.489	< .001	0.703	0.831
Conn_4	0.513	0.513	0.056	9.216	< .001	0.404	0.622
Contribution =~							
Cont_1	0.601	0.601	0.081	7.419	< .001	0.442	0.760

Cont_2	0.668	0.668	0.064	10.481	< .001	0.543	0.793
Cont_3	0.457	0.457	0.085	5.373	< .001	0.291	0.624
Cont_4	0.659	0.659	0.070	9.439	< .001	0.522	0.796
Cont_5	0.613	0.613	0.070	8.786	< .001	0.477	0.750
Cont_6	0.491	0.491	0.088	5.590	< .001	0.319	0.663
Creativity =~							
Crea_1	0.801	0.801	0.024	33.962	< .001	0.754	0.847
Crea_2	0.910	0.910	0.014	64.907	< .001	0.882	0.937
Crea_3	0.893	0.893	0.016	55.858	< .001	0.862	0.924
Crea_4	0.750	0.750	0.027	28.231	< .001	0.698	0.802
Crea_5	0.871	0.871	0.018	47.798	< .001	0.835	0.907
Crea_6	0.879	0.879	0.016	54.040	< .001	0.847	0.911
Crea_7	0.795	0.795	0.024	33.155	< .001	0.748	0.843
Crea_8	0.962	0.962	0.009	109.347	< .001	0.945	0.980
Crea_9	0.946	0.946	0.011	89.518	< .001	0.925	0.967
Hope =~							
Hope_1	0.880	0.880	0.020	43.478	< .001	0.840	0.920
Hope_2	0.737	0.737	0.032	22.881	< .001	0.674	0.801
Hope_3	0.643	0.643	0.037	17.419	< .001	0.570	0.715
Hope_4	0.783	0.783	0.031	25.473	< .001	0.723	0.843
Hope_5	0.600	0.600	0.043	13.992	< .001	0.516	0.684
Hope_6	0.576	0.576	0.042	13.653	< .001	0.493	0.659
Hope_7	0.634	0.634	0.048	13.146	< .001	0.539	0.728
Hope_8	0.754	0.754	0.031	24.389	< .001	0.693	0.815
Hope_9	0.707	0.707	0.033	21.516	< .001	0.643	0.772
Hope_10	0.758	0.758	0.029	25.887	< .001	0.701	0.816
Hope_11	0.772	0.772	0.028	27.190	< .001	0.717	0.828
Hope_12	0.895	0.895	0.016	55.779	< .001	0.864	0.926

Life Satisfaction =~							
SWLS_1	0.856	0.856	0.024	35.004	< .001	0.808	0.904
SWLS_2	0.798	0.798	0.026	30.146	< .001	0.746	0.850
SWLS_3	0.949	0.949	0.019	51.122	< .001	0.913	0.985

Note: β = standardized coefficient; b = unstandardized coefficient; SE = standard error; ULCI = upper limit confidence interval; LLCI = lower limit confidence interval.

Table G.6.
Factor Loadings of the Measurement Model (Step 1) of the 7Cs of Positive Youth Development, Hope, and Life Satisfaction Using the Youth Sample from Thailand (n = 486)

Items	*β*	*b*	SE	z-value	*p*-value	95% ULCI	95% LLCI
Competence =~							
Comp_1	0.578	0.578	0.037	15.535	< .001	0.505	0.650
Comp_2	0.830	0.830	0.039	21.042	< .001	0.752	0.907
Comp_3	0.467	0.467	0.052	5.124	< .001	0.365	0.570
Confidence =~							
Conf_1	0.676	0.676	0.034	19.603	< .001	0.608	0.744
Conf_2	0.892	0.892	0.016	54.852	< .001	0.860	0.923
Conf_3	0.944	0.944	0.015	62.024	< .001	0.914	0.974
Character =~							
Char_1	0.466	0.466	0.038	12.268	< .001	0.392	0.541
Char_2	0.789	0.789	0.021	37.168	< .001	0.748	0.831
Char_3	0.793	0.793	0.025	32.055	< .001	0.744	0.841
Char_4	0.642	0.642	0.030	21.455	< .001	0.583	0.700
Caring =~							
Care_1	0.897	0.897	0.021	42.388	< .001	0.855	0.938
Care_2	0.859	0.859	0.016	53.064	< .001	0.827	0.890
Care_3	0.957	0.957	0.013	75.721	< .001	0.932	0.982
Connection =~							
Conn_1	0.746	0.746	0.025	29.955	< .001	0.698	0.795
Conn_2	0.815	0.815	0.023	35.183	< .001	0.769	0.860
Conn_3	0.564	0.564	0.030	18.760	< .001	0.505	0.623
Conn_4	0.659	0.659	0.028	23.769	< .001	0.604	0.713
Contribution =~							
Cont_1	0.621	0.621	0.040	15.548	< .001	0.543	0.700

Cont_2	0.723	0.723	0.029	24.786	< .001	0.666	0.780
Cont_3	0.843	0.843	0.032	26.037	< .001	0.780	0.907
Cont_4	0.831	0.831	0.027	30.691	< .001	0.778	0.884
Cont_5	0.805	0.805	0.029	28.240	< .001	0.749	0.861
Cont_6	0.686	0.686	0.035	19.337	< .001	0.616	0.756
Creativity =~							
Crea_1	0.821	0.821	0.019	43.789	< .001	0.784	0.858
Crea_2	0.841	0.841	0.016	54.193	< .001	0.811	0.871
Crea_3	0.859	0.859	0.014	59.686	< .001	0.831	0.887
Crea_4	0.827	0.827	0.017	48.985	< .001	0.794	0.860
Crea_5	0.837	0.837	0.015	56.055	< .001	0.807	0.866
Crea_6	0.824	0.824	0.014	57.004	< .001	0.796	0.853
Crea_7	0.779	0.779	0.020	38.363	< .001	0.739	0.819
Crea_8	0.932	0.932	0.008	110.262	< .001	0.916	0.949
Crea_9	0.944	0.944	0.008	114.918	< .001	0.928	0.960
Hope =~							
Hope_1	0.769	0.769	0.020	38.519	< .001	0.730	0.808
Hope_2	0.764	0.764	0.022	34.400	< .001	0.721	0.808
Hope_3	0.767	0.767	0.021	37.416	< .001	0.727	0.807
Hope_4	0.752	0.752	0.023	32.924	< .001	0.707	0.796
Hope_5	0.830	0.830	0.017	49.896	< .001	0.798	0.863
Hope_6	0.481	0.481	0.033	14.692	< .001	0.417	0.545
Hope_7	0.788	0.788	0.022	36.427	< .001	0.746	0.830
Hope_8	0.725	0.725	0.022	32.500	< .001	0.681	0.768
Hope_9	0.728	0.728	0.021	35.276	< .001	0.688	0.769
Hope_10	0.718	0.718	0.023	31.649	< .001	0.674	0.763
Hope_11	0.826	0.826	0.017	48.515	< .001	0.793	0.860
Hope_12	0.843	0.843	0.018	46.782	< .001	0.808	0.879

Life Satisfaction =~							
SWLS_1	0.873	0.873	0.019	45.576	< .001	0.835	0.911
SWLS_2	0.910	0.910	0.018	50.716	< .001	0.875	0.945
SWLS_3	0.861	0.861	0.021	41.373	< .001	0.820	0.902

Note: β = standardized coefficient; *b* = unstandardized coefficient; SE = standard error; ULCI = upper limit confidence interval; LLCI = lower limit confidence interval.

Table G.7.
Factor Loadings of the Measurement Model (Step 1) of the 7Cs of Positive Youth Development, Hope, and Life Satisfaction Using the Youth Sample from Vietnam (n = 248)

Items	*β*	*b*	SE	z-value	*p*-value	95% ULCI	95% LLCI
Competence =~							
Comp_1	0.684	0.684	0.037	18.283	< .001	0.610	0.757
Comp_2	0.783	0.783	0.032	24.220	< .001	0.720	0.847
Comp_3	0.494	0.494	0.058	6.843	< .001	0.381	0.606
Confidence =~							
Conf_1	0.808	0.808	0.027	30.409	< .001	0.756	0.860
Conf_2	0.593	0.593	0.044	13.393	< .001	0.507	0.680
Conf_3	0.901	0.901	0.029	31.180	< .001	0.844	0.958
Character =~							
Char_1	0.450	0.450	0.054	8.287	< .001	0.343	0.556
Char_2	0.938	0.938	0.023	41.622	< .001	0.894	0.982
Char_3	0.875	0.875	0.020	44.609	< .001	0.836	0.913
Char_4	0.429	0.429	0.054	0.532	< .001	0.378	0.536
Caring =~							
Care_1	0.907	0.907	0.021	42.882	< .001	0.866	0.949
Care_2	0.661	0.661	0.039	16.771	< .001	0.584	0.738
Care_3	0.898	0.898	0.024	37.654	< .001	0.852	0.945
Connection =~							
Conn_1	0.693	0.693	0.039	17.714	< .001	0.616	0.770
Conn_2	0.812	0.812	0.035	23.112	< .001	0.743	0.881
Conn_3	0.599	0.599	0.042	14.148	< .001	0.516	0.682
Conn_4	0.650	0.650	0.041	16.011	< .001	0.571	0.730
Contribution =~							
Cont_1	0.849	0.849	0.033	25.885	< .001	0.784	0.913

Cont_2	0.814	0.814	0.032	25.819	< .001	0.753	0.876
Cont_3	0.827	0.827	0.052	15.835	< .001	0.725	0.930
Cont_4	0.699	0.699	0.037	19.137	< .001	0.628	0.771
Cont_5	0.672	0.672	0.045	14.836	< .001	0.583	0.761
Cont_6	0.655	0.655	0.048	13.559	< .001	0.560	0.749
Creativity =~							
Crea_1	0.742	0.742	0.033	22.733	< .001	0.678	0.806
Crea_2	0.855	0.855	0.020	43.526	< .001	0.817	0.894
Crea_3	0.876	0.876	0.022	40.026	< .001	0.833	0.919
Crea_4	0.843	0.843	0.022	38.164	< .001	0.800	0.886
Crea_5	0.742	0.742	0.029	25.983	< .001	0.686	0.798
Crea_6	0.744	0.744	0.028	26.783	< .001	0.689	0.798
Crea_7	0.834	0.834	0.026	32.669	< .001	0.784	0.884
Crea_8	0.898	0.898	0.019	46.335	< .001	0.860	0.935
Crea_9	0.878	0.878	0.018	48.410	< .001	0.843	0.914
Hope =~							
Hope_1	0.794	0.794	0.025	31.952	< .001	0.745	0.842
Hope_2	0.710	0.710	0.031	22.774	< .001	0.649	0.771
Hope_3	0.552	0.552	0.040	13.905	< .001	0.474	0.630
Hope_4	0.851	0.851	0.021	40.694	< .001	0.810	0.892
Hope_5	0.728	0.728	0.030	24.361	< .001	0.669	0.786
Hope_6	0.667	0.667	0.033	20.137	< .001	0.602	0.732
Hope_7	0.728	0.728	0.028	25.747	< .001	0.673	0.784
Hope_8	0.788	0.788	0.027	29.695	< .001	0.736	0.840
Hope_9	0.813	0.813	0.024	33.936	< .001	0.766	0.860
Hope_10	0.815	0.815	0.024	34.296	< .001	0.769	0.862
Hope_11	0.845	0.845	0.021	40.693	< .001	0.804	0.886
Hope_12	0.877	0.877	0.021	42.616	< .001	0.837	0.918

Life Satisfaction =~							
SWLS_1	0.869	0.869	0.023	37.101	< .001	0.823	0.915
SWLS_2	0.818	0.818	0.027	29.749	< .001	0.764	0.872
SWLS_3	0.932	0.932	0.022	43.117	< .001	0.889	0.974

Note: β = standardized coefficient; *b* = unstandardized coefficient; SE = standard error; ULCI = upper limit confidence interval; LLCI = lower limit confidence interval.

Supplementary Material H

Table H.1.
Factor Loadings of the Structural Equation Model (Step 2) of the 7Cs of Positive Youth Development, Hope, and Life Satisfaction Controlling for Gender, Age, and Education Using the Overall Youth Sample (n = 1,967)

Items	*β*	*b*	SE	z-value	*p*-value	95% ULCI	95% LLCI
Competence =~							
Comp_1	0.641	0.641	0.018	35.471	< .001	0.606	0.677
Comp_2	0.752	0.752	0.017	44.655	< .001	0.719	0.785
Comp_3	0.401	0.401	0.023	17.587	< .001	0.356	0.445
Confidence =~							
Conf_1	0.781	0.781	0.013	58.266	< .001	0.755	0.807
Conf_2	0.737	0.737	0.013	57.638	< .001	0.712	0.762
Conf_3	0.919	0.919	0.010	93.492	< .001	0.899	0.938
Character =~							
Char_1	0.424	0.424	0.021	19.787	< .001	0.382	0.466
Char_2	0.725	0.725	0.015	49.619	< .001	0.696	0.753
Char_3	0.767	0.767	0.014	55.184	< .001	0.740	0.795
Char_4	0.596	0.596	0.017	35.191	< .001	0.563	0.629
Caring =~							
Care_1	0.766	0.766	0.015	49.938	< .001	0.736	0.797
Care_2	0.869	0.869	0.010	83.291	< .001	0.849	0.890
Care_3	0.960	0.960	0.010	99.729	< .001	0.941	0.978
Connection =~							
Conn_1	0.686	0.686	0.015	47.103	< .001	0.657	0.714
Conn_2	0.769	0.769	0.013	59.430	< .001	0.744	0.795
Conn_3	0.661	0.661	0.014	46.953	< .001	0.633	0.689
Conn_4	0.570	0.570	0.019	30.292	< .001	0.533	0.607
Contribution =~							

Cont_1	0.681	0.681	0.023	30.026	< .001	0.636	0.725
Cont_2	0.672	0.672	0.020	34.200	< .001	0.634	0.711
Cont_3	0.691	0.691	0.024	28.307	< .001	0.643	0.739
Cont_4	0.701	0.701	0.019	37.400	< .001	0.665	0.738
Cont_5	0.661	0.661	0.021	31.162	< .001	0.619	0.702
Cont_6	0.648	0.648	0.023	28.139	< .001	0.603	0.694
Creativity =~							
Crea_1	0.720	0.720	0.012	62.074	< .001	0.697	0.743
Crea_2	0.779	0.779	0.009	88.323	< .001	0.762	0.797
Crea_3	0.797	0.797	0.008	99.729	< .001	0.781	0.812
Crea_4	0.732	0.732	0.010	74.471	< .001	0.713	0.751
Crea_5	0.979	0.979	0.005	205.045	< .001	0.970	0.989
Crea_6	0.978	0.978	0.005	199.331	< .001	0.968	0.987
Crea_7	0.948	0.948	0.004	229.154	< .001	0.940	0.956
Crea_8	0.847	0.847	0.006	149.886	< .001	0.836	0.858
Crea_9	0.815	1.015	0.004	247.348	< .001	0.807	0.923
Hope =~							
Hope_1	0.794	0.473	0.012	38.558	< .001	0.449	0.497
Hope_2	0.746	0.444	0.012	37.276	< .001	0.421	0.468
Hope_3	0.690	0.411	0.012	35.526	< .001	0.388	0.433
Hope_4	0.766	0.456	0.012	38.027	< .001	0.433	0.480
Hope_5	0.737	0.439	0.012	36.569	< .001	0.415	0.462
Hope_6	0.524	0.312	0.012	26.347	< .001	0.288	0.335
Hope_7	0.728	0.434	0.012	35.321	< .001	0.410	0.458
Hope_8	0.747	0.445	0.012	37.882	< .001	0.422	0.468
Hope_9	0.740	0.441	0.012	37.393	< .001	0.418	0.464
Hope_10	0.763	0.455	0.012	37.545	< .001	0.431	0.478
Hope_11	0.841	0.501	0.012	40.165	< .001	0.477	0.526

Hope_12	0.846	0.504	0.013	39.783	< .001	0.479	0.529
Life Satisfaction =~							
SWLS_1	0.843	0.614	0.014	44.010	< .001	0.587	0.642
SWLS_2	0.850	0.619	0.012	49.785	< .001	0.595	0.643
SWLS_3	0.884	0.645	0.014	46.793	< .001	0.618	0.672

Note: β = standardized coefficient; *b* = unstandardized coefficient; SE = standard error; ULCI = upper limit confidence interval; LLCI = lower limit confidence interval.

Table H.2.
Factor Loadings of the Structural Equation Model (Step 2) of the 7Cs of Positive Youth Development, Hope, and Life Satisfaction Controlling for Gender, Age, and Education Using the Youth Sample from Indonesia (n = 227)

Items	*β*	*b*	SE	z-value	*p*-value	95% ULCI	95% LLCI
Competence =~							
Comp_1	0.769	0.769	0.043	18.019	< .001	0.685	0.853
Comp_2	0.748	0.748	0.043	17.398	< .001	0.664	0.832
Comp_3	0.464	0.464	0.069	5.278	< .001	0.429	0.500
Confidence =~							
Conf_1	0.853	0.853	0.026	32.471	< .001	0.802	0.904
Conf_2	0.840	0.840	0.023	36.467	< .001	0.795	0.885
Conf_3	0.917	0.917	0.019	47.357	< .001	0.879	0.955
Character =~							
Char_1	0.402	0.402	0.062	6.512	< .001	0.281	0.523
Char_2	0.563	0.563	0.047	12.003	< .001	0.471	0.655
Char_3	0.697	0.697	0.049	14.312	< .001	0.602	0.793
Char_4	0.618	0.618	0.042	14.746	< .001	0.536	0.700
Caring =~							
Care_1	0.808	0.808	0.037	21.565	< .001	0.735	0.882
Care_2	0.848	0.848	0.031	27.555	< .001	0.787	0.908
Care_3	0.943	0.943	0.030	31.148	< .001	0.884	1.002
Connection =~							
Conn_1	0.666	0.666	0.044	15.106	< .001	0.579	0.752
Conn_2	0.721	0.721	0.036	19.783	< .001	0.650	0.793
Conn_3	0.734	0.734	0.035	21.250	< .001	0.666	0.802
Conn_4	0.602	0.602	0.052	11.550	< .001	0.500	0.704
Contribution =~							
Cont_1	0.509	0.509	0.074	6.913	< .001	0.365	0.654

Cont_2	0.562	0.562	0.070	7.993	< .001	0.424	0.699
Cont_3	0.714	0.714	0.055	12.952	< .001	0.606	0.822
Cont_4	0.737	0.737	0.052	14.116	< .001	0.635	0.840
Cont_5	0.544	0.544	0.065	8.366	< .001	0.416	0.671
Cont_6	0.628	0.628	0.067	9.448	< .001	0.498	0.759
Creativity =~							
Crea_1	0.737	0.737	0.044	16.660	< .001	0.650	0.823
Crea_2	0.761	0.761	0.033	23.345	< .001	0.697	0.825
Crea_3	0.763	0.763	0.034	22.114	< .001	0.695	0.830
Crea_4	0.575	0.575	0.043	13.296	< .001	0.490	0.660
Crea_5	0.660	0.660	0.039	16.754	< .001	0.583	0.738
Crea_6	0.790	0.790	0.032	24.711	< .001	0.728	0.853
Crea_7	0.849	0.849	0.026	32.462	< .001	0.798	0.900
Crea_8	0.765	0.765	0.028	27.411	< .001	0.710	0.820
Crea_9	0.855	0.855	0.023	36.530	< .001	0.809	0.901
Hope =~							
Hope_1	0.773	0.398	0.041	9.707	< .001	0.318	0.478
Hope_2	0.561	0.289	0.034	8.509	< .001	0.222	0.355
Hope_3	0.643	0.331	0.037	8.983	< .001	0.259	0.403
Hope_4	0.695	0.357	0.039	9.165	< .001	0.281	0.434
Hope_5	0.691	0.355	0.038	9.456	< .001	0.282	0.429
Hope_6	0.633	0.326	0.035	9.393	< .001	0.258	0.394
Hope_7	0.672	0.346	0.041	8.430	< .001	0.265	0.426
Hope_8	0.622	0.320	0.036	8.982	< .001	0.250	0.390
Hope_9	0.704	0.362	0.040	9.161	< .001	0.284	0.439
Hope_10	0.739	0.380	0.040	9.587	< .001	0.303	0.458
Hope_11	0.813	0.419	0.042	10.055	< .001	0.337	0.500
Hope_12	0.724	0.372	0.039	9.639	< .001	0.297	0.448

Life Satisfaction =~							
SWLS_1	0.788	0.530	0.065	8.124	< .001	0.402	0.658
SWLS_2	0.853	0.575	0.066	8.701	< .001	0.445	0.704
SWLS_3	0.857	0.577	0.069	8.411	< .001	0.443	0.711

Note: *β* = standardized coefficient; *b* = unstandardized coefficient; SE = standard error; ULCI = upper limit confidence interval; LLCI = lower limit confidence interval.

Table H.3.
Factor Loadings of the Structural Equation Model (Step 2) of the 7Cs of Positive Youth Development, Hope, and Life Satisfaction Controlling for Gender, Age, and Education Using the Youth Sample from Malaysia (n = 280)

Items	*β*	*b*	SE	z-value	*p*-value	95% ULCI	95% LLCI
Competence =~							
Comp_1	0.533	0.599	0.072	8.330	< .001	0.458	0.740
Comp_2	0.622	0.535	0.053	10.144	< .001	0.431	0.638
Comp_3	0.505	0.636	0.076	8.320	< .001	0.486	0.786
Confidence =~							
Conf_1	0.788	0.877	0.056	15.598	< .001	0.766	0.987
Conf_2	0.734	0.813	0.058	14.106	< .001	0.700	0.926
Conf_3	0.864	0.915	0.052	17.579	< .001	0.813	1.017
Character =~							
Char_1	0.425	0.358	0.063	5.646	< .001	0.234	0.482
Char_2	0.504	0.434	0.050	8.640	< .001	0.336	0.533
Char_3	0.772	0.583	0.033	17.641	< .001	0.518	0.647
Char_4	0.610	0.502	0.043	11.796	< .001	0.419	0.586
Caring =~							
Care_1	0.486	0.336	0.061	5.491	< .001	0.216	0.456
Care_2	0.733	0.511	0.035	14.596	< .001	0.442	0.580
Care_3	0.865	0.528	0.037	14.270	< .001	0.456	0.601
Connection =~							
Conn_1	0.627	0.682	0.060	11.374	< .001	0.565	0.800
Conn_2	0.736	0.772	0.056	13.795	< .001	0.662	0.881
Conn_3	0.763	0.942	0.054	17.507	< .001	0.837	1.048
Conn_4	0.512	0.552	0.062	8.844	< .001	0.430	0.674
Contribution =~							
Cont_1	0.682	0.832	0.074	11.249	< .001	0.687	0.977

Cont_2	0.711	0.848	0.061	13.833	< .001	0.728	0.969
Cont_3	0.431	0.517	0.064	8.099	< .001	0.392	0.642
Cont_4	0.621	0.743	0.061	12.123	< .001	0.623	0.863
Cont_5	0.704	1.028	0.077	13.405	< .001	0.877	1.178
Cont_6	0.748	1.115	0.066	16.980	< .001	0.986	1.243
Creativity =~							
Crea_1	0.757	0.738	0.050	14.700	< .001	0.639	0.836
Crea_2	0.812	0.803	0.049	16.329	< .001	0.707	0.900
Crea_3	0.865	0.851	0.044	19.440	< .001	0.766	0.937
Crea_4	0.858	0.899	0.044	20.612	< .001	0.813	0.984
Crea_5	0.839	0.841	0.049	17.129	< .001	0.744	0.937
Crea_6	0.826	0.782	0.048	16.462	< .001	0.689	0.875
Crea_7	0.856	0.849	0.044	19.338	< .001	0.763	0.935
Crea_8	0.893	0.866	0.042	20.846	< .001	0.785	0.948
Crea_9	0.866	0.866	0.045	19.440	< .001	0.779	0.954
Hope =~							
Hope_1	0.772	0.177	0.380	0.467	< .001	0.568	0.923
Hope_2	0.739	0.166	0.358	0.464	< .001	0.535	0.868
Hope_3	0.700	0.207	0.447	0.465	< .001	0.668	0.813
Hope_4	0.714	0.157	0.340	0.463	< .001	0.509	0.824
Hope_5	0.718	0.160	0.342	0.467	< .001	0.510	0.829
Hope_6	0.485	0.150	0.326	0.461	< .001	0.489	0.789
Hope_7	0.716	0.173	0.373	0.465	< .001	0.557	0.904
Hope_8	0.720	0.178	0.381	0.466	< .001	0.569	0.924
Hope_9	0.625	0.151	0.328	0.461	< .001	0.491	0.793
Hope_10	0.774	0.198	0.429	0.463	< .001	0.642	0.831
Hope_11	0.820	0.194	0.417	0.466	< .001	0.623	0.912
Hope_12	0.871	0.214	0.459	0.466	< .001	0.686	0.914

Life Satisfaction =~							
SWLS_1	0.663	0.547	0.084	6.500	< .001	0.382	0.712
SWLS_2	0.933	0.791	0.089	8.849	< .001	0.615	0.966
SWLS_3	0.919	0.782	0.092	8.475	< .001	0.601	0.963

Note: *β* = standardized coefficient; *b* = unstandardized coefficient; SE = standard error; ULCI = upper limit confidence interval; LLCI = lower limit confidence interval.

Table H.4.
Factor Loadings of the Structural Equation Model (Step 2) of the 7Cs of Positive Youth Development, Hope, and Life Satisfaction Controlling for Gender, Age, and Education Using the Youth Sample from the Philippines (n = 479)

Items	*β*	*b*	SE	z-value	*p*-value	95% ULCI	95% LLCI
Competence =~							
Comp_1	0.669	0.669	0.038	17.581	< .001	0.594	0.744
Comp_2	0.694	0.694	0.035	19.899	< .001	0.625	0.762
Comp_3	0.496	0.496	0.040	12.315	< .001	0.417	0.574
Confidence =~							
Conf_1	0.822	0.822	0.028	29.108	< .001	0.766	0.877
Conf_2	0.682	0.682	0.030	22.632	< .001	0.623	0.741
Conf_3	0.922	0.922	0.025	37.288	< .001	0.874	0.971
Character =~							
Char_1	0.483	0.483	0.043	8.836	< .001	0.298	0.467
Char_2	0.761	0.761	0.029	26.175	< .001	0.704	0.818
Char_3	0.733	0.733	0.031	23.856	< .001	0.672	0.793
Char_4	0.710	0.710	0.033	21.298	< .001	0.645	0.775
Caring =~							
Care_1	0.922	0.922	0.023	40.907	< .001	0.878	0.966
Care_2	0.886	0.886	0.018	49.812	< .001	0.852	0.921
Care_3	0.889	0.889	0.017	52.024	< .001	0.856	0.923
Connection =~							
Conn_1	0.680	0.680	0.033	20.402	< .001	0.615	0.746
Conn_2	0.811	0.811	0.026	30.836	< .001	0.759	0.862
Conn_3	0.719	0.719	0.027	26.573	< .001	0.666	0.772
Conn_4	0.474	0.474	0.048	9.838	< .001	0.380	0.569
Contribution =~							
Cont_1	0.809	0.809	0.042	19.266	< .001	0.727	0.891

Cont_2	0.621	0.621	0.046	13.628	< .001	0.531	0.710
Cont_3	0.537	0.537	0.059	9.102	< .001	0.421	0.652
Cont_4	0.710	0.710	0.038	18.803	< .001	0.636	0.784
Cont_5	0.575	0.575	0.050	11.439	< .001	0.476	0.673
Cont_6	0.631	0.631	0.048	13.200	< .001	0.537	0.724
Creativity =~							
Crea_1	0.660	0.660	0.026	25.032	< .001	0.608	0.712
Crea_2	0.798	0.798	0.018	44.493	< .001	0.763	0.833
Crea_3	0.853	0.853	0.014	62.699	< .001	0.826	0.879
Crea_4	0.693	0.693	0.024	29.331	< .001	0.646	0.739
Crea_5	0.839	0.839	0.015	56.217	< .001	0.810	0.869
Crea_6	0.829	0.829	0.017	48.770	< .001	0.796	0.863
Crea_7	0.812	0.812	0.018	45.858	< .001	0.778	0.847
Crea_8	0.878	0.878	0.012	74.361	< .001	0.855	0.901
Crea_9	0.895	0.895	0.013	71.480	< .001	0.870	0.919
Hope =~							
Hope_1	0.795	0.518	0.028	18.404	< .001	0.463	0.573
Hope_2	0.750	0.488	0.026	18.999	< .001	0.438	0.539
Hope_3	0.702	0.456	0.026	17.774	< .001	0.406	0.507
Hope_4	0.742	0.483	0.025	19.020	< .001	0.433	0.533
Hope_5	0.747	0.486	0.026	19.044	< .001	0.436	0.536
Hope_6	0.510	0.331	0.027	12.356	< .001	0.279	0.384
Hope_7	0.711	0.463	0.025	18.860	< .001	0.415	0.511
Hope_8	0.784	0.511	0.027	19.078	< .001	0.458	0.563
Hope_9	0.786	0.512	0.026	19.788	< .001	0.461	0.562
Hope_10	0.757	0.492	0.026	18.746	< .001	0.441	0.544
Hope_11	0.870	0.567	0.027	20.621	< .001	0.513	0.621
Hope_12	0.831	0.541	0.027	20.409	< .001	0.489	0.593

Life Satisfaction =~							
SWLS_1	0.872	0.612	0.037	16.592	< .001	0.539	0.684
SWLS_2	0.788	0.553	0.031	17.649	< .001	0.491	0.614
SWLS_3	0.861	0.604	0.037	16.498	< .001	0.532	0.676

Note: *β* = standardized coefficient; *b* = unstandardized coefficient; SE = standard error; ULCI = upper limit confidence interval; LLCI = lower limit confidence interval.

Table H.5.
Factor Loadings of the Structural Equation Model (Step 2) of the 7Cs of Positive Youth Development, Hope, and Life Satisfaction Controlling for Gender, Age, and Education Using the Youth Sample from Singapore (n = 247)

Items	*β*	*b*	SE	z-value	*p*-value	95% ULCI	95% LLCI
Competence =~							
Comp_1	0.550	0.550	0.065	8.479	< .001	0.423	0.677
Comp_2	0.697	0.697	0.060	11.581	< .001	0.579	0.815
Comp_3	0.411	0.411	0.066	6.265	< .001	0.283	0.540
Confidence =~							
Conf_1	0.904	0.904	0.024	37.776	< .001	0.857	0.950
Conf_2	0.690	0.690	0.034	20.005	< .001	0.622	0.758
Conf_3	0.876	0.876	0.023	38.039	< .001	0.831	0.921
Character =~							
Char_1	0.472	0.472	0.063	4.320	< .001	0.149	0.596
Char_2	0.751	0.751	0.040	18.734	< .001	0.672	0.830
Char_3	0.618	0.618	0.047	13.070	< .001	0.526	0.711
Char_4	0.705	0.705	0.040	17.581	< .001	0.626	0.783
Caring =~							
Care_1	0.864	0.864	0.036	24.210	< .001	0.794	0.934
Care_2	0.882	0.882	0.026	33.290	< .001	0.830	0.933
Care_3	0.880	0.880	0.025	35.019	< .001	0.830	0.929
Connection =~							
Conn_1	0.562	0.562	0.048	11.608	< .001	0.467	0.656
Conn_2	0.736	0.736	0.036	20.427	< .001	0.665	0.806
Conn_3	0.768	0.768	0.032	24.089	< .001	0.705	0.830
Conn_4	0.515	0.515	0.054	9.609	< .001	0.410	0.620
Contribution =~							
Cont_1	0.614	0.614	0.080	7.699	< .001	0.458	0.771

Cont_2	0.671	0.671	0.063	10.632	< .001	0.548	0.795
Cont_3	0.478	0.478	0.079	6.016	< .001	0.322	0.634
Cont_4	0.650	0.650	0.067	9.696	< .001	0.519	0.782
Cont_5	0.640	0.640	0.066	9.697	< .001	0.511	0.769
Cont_6	0.497	0.497	0.084	5.897	< .001	0.332	0.662
Creativity =~							
Crea_1	0.799	0.799	0.024	33.332	< .001	0.752	0.846
Crea_2	0.905	0.905	0.015	59.702	< .001	0.875	0.934
Crea_3	0.890	0.890	0.017	52.487	< .001	0.857	0.923
Crea_4	0.743	0.743	0.027	27.344	< .001	0.689	0.796
Crea_5	0.863	0.863	0.019	45.722	< .001	0.826	0.900
Crea_6	0.876	0.876	0.017	52.578	< .001	0.843	0.908
Crea_7	0.780	0.780	0.025	31.271	< .001	0.731	0.829
Crea_8	0.992	0.992	0.009	114.037	< .001	0.975	1.009
Crea_9	0.978	0.978	0.009	108.890	< .001	0.960	0.996
Hope =~							
Hope_1	0.886	0.345	0.032	10.881	< .001	0.283	0.407
Hope_2	0.742	0.289	0.027	10.550	< .001	0.235	0.343
Hope_3	0.641	0.250	0.025	9.905	< .001	0.200	0.299
Hope_4	0.787	0.307	0.030	10.118	< .001	0.247	0.366
Hope_5	0.611	0.238	0.025	9.595	< .001	0.189	0.286
Hope_6	0.575	0.224	0.026	8.494	< .001	0.172	0.276
Hope_7	0.637	0.248	0.029	8.474	< .001	0.191	0.306
Hope_8	0.760	0.296	0.029	10.309	< .001	0.240	0.352
Hope_9	0.711	0.277	0.027	10.199	< .001	0.224	0.330
Hope_10	0.762	0.297	0.028	10.764	< .001	0.243	0.351
Hope_11	0.779	0.304	0.028	10.963	< .001	0.249	0.358
Hope_12	0.895	0.349	0.032	10.995	< .001	0.287	0.411

Life Satisfaction =~							
SWLS_1	0.857	0.552	0.037	14.994	< .001	0.480	0.624
SWLS_2	0.802	0.516	0.031	16.495	< .001	0.455	0.578
SWLS_3	0.952	0.613	0.040	15.512	< .001	0.536	0.691

Note: *β* = standardized coefficient; *b* = unstandardized coefficient; SE = standard error; ULCI = upper limit confidence interval; LLCI = lower limit confidence interval.

Table H.6.
Factor Loadings of the Structural Equation Model (Step 2) of the 7Cs of Positive Youth Development, Hope, and Life Satisfaction Controlling for Gender, Age, and Education Using the Youth Sample from Thailand (n = 486)

Items	*β*	*b*	SE	z-value	*p*-value	95% ULCI	95% LLCI
Competence =~							
Comp_1	0.586	0.582	0.048	12.139	< .001	0.488	0.675
Comp_2	0.710	0.581	0.044	13.144	< .001	0.494	0.668
Comp_3	0.418	0.425	0.060	5.454	< .001	0.308	0.541
Confidence =~							
Conf_1	0.555	0.543	0.045	12.087	< .001	0.455	0.631
Conf_2	0.860	0.804	0.031	25.910	< .001	0.743	0.865
Conf_3	0.908	0.868	0.031	27.736	< .001	0.807	0.930
Character =~							
Char_1	0.404	0.419	0.045	9.294	< .001	0.331	0.507
Char_2	0.750	0.651	0.034	19.320	< .001	0.585	0.717
Char_3	0.772	0.654	0.031	21.113	< .001	0.593	0.715
Char_4	0.547	0.539	0.041	13.115	< .001	0.458	0.619
Caring =~							
Care_1	0.751	0.651	0.035	18.793	< .001	0.583	0.719
Care_2	0.871	0.744	0.028	26.863	< .001	0.690	0.798
Care_3	0.909	0.770	0.027	28.943	< .001	0.718	0.822
Connection =~							
Conn_1	0.733	0.733	0.038	19.033	< .001	0.657	0.808
Conn_2	0.740	0.743	0.041	18.324	< .001	0.664	0.823
Conn_3	0.512	0.591	0.049	11.943	< .001	0.494	0.688
Conn_4	0.595	0.488	0.039	12.561	< .001	0.412	0.565
Contribution =~							
Cont_1	0.608	0.786	0.057	13.865	< .001	0.675	0.897

Cont_2	0.737	0.901	0.045	19.833	< .001	0.812	0.990
Cont_3	0.688	0.888	0.052	17.005	< .001	0.785	0.990
Cont_4	0.759	0.923	0.044	21.177	< .001	0.838	1.008
Cont_5	0.719	0.984	0.047	20.903	< .001	0.892	1.076
Cont_6	0.599	0.804	0.056	14.320	< .001	0.694	0.914
Creativity =~							
Crea_1	0.702	0.562	0.031	18.409	< .001	0.502	0.622
Crea_2	0.806	0.703	0.033	21.515	< .001	0.639	0.767
Crea_3	0.804	0.699	0.032	21.894	< .001	0.637	0.762
Crea_4	0.756	0.638	0.032	20.135	< .001	0.576	0.700
Crea_5	0.791	0.740	0.033	22.247	< .001	0.675	0.805
Crea_6	0.805	0.746	0.036	20.617	< .001	0.676	0.817
Crea_7	0.696	0.597	0.034	17.333	< .001	0.530	0.665
Crea_8	0.872	0.821	0.032	25.371	< .001	0.758	0.885
Crea_9	0.872	0.854	0.032	26.688	< .001	0.791	0.917
Hope =~							
Hope_1	0.738	0.387	0.029	13.334	< .001	0.330	0.443
Hope_2	0.710	0.396	0.032	12.425	< .001	0.333	0.458
Hope_3	0.722	0.466	0.035	13.291	< .001	0.398	0.535
Hope_4	0.722	0.394	0.033	11.893	< .001	0.329	0.459
Hope_5	0.781	0.440	0.033	13.395	< .001	0.376	0.505
Hope_6	0.466	0.320	0.037	8.679	< .001	0.248	0.392
Hope_7	0.709	0.395	0.034	11.636	< .001	0.328	0.461
Hope_8	0.696	0.399	0.033	11.949	< .001	0.333	0.464
Hope_9	0.641	0.338	0.027	12.587	< .001	0.285	0.390
Hope_10	0.672	0.387	0.032	12.230	< .001	0.325	0.450
Hope_11	0.773	0.420	0.031	13.586	< .001	0.359	0.481
Hope_12	0.782	0.444	0.034	13.211	< .001	0.378	0.510

Life Satisfaction =~							
SWLS_1	0.878	0.948	0.063	14.958	< .001	0.823	1.072
SWLS_2	0.893	1.070	0.070	15.394	< .001	0.934	1.206
SWLS_3	0.795	0.964	0.064	15.056	< .001	0.839	1.090

Note: β = standardized coefficient; *b* = unstandardized coefficient; SE = standard error; ULCI = upper limit confidence interval; LLCI = lower limit confidence interval.

Table H.7.
Factor Loadings of the Structural Equation Model (Step 2) of the 7Cs of Positive Youth Development, Hope, and Life Satisfaction Controlling for Gender, Age, and Education Using the Youth Sample from Vietnam (n = 248)

Items	*β*	*b*	SE	z-value	*p*-value	95% ULCI	95% LLCI
Competence =~							
Comp_1	0.679	0.657	0.069	9.538	< .001	0.522	0.792
Comp_2	0.739	0.599	0.052	11.442	< .001	0.496	0.701
Comp_3	0.458	0.466	0.081	4.497	< .001	0.207	0.526
Confidence =~							
Conf_1	0.764	0.676	0.051	13.248	< .001	0.576	0.776
Conf_2	0.553	0.513	0.065	7.954	< .001	0.387	0.640
Conf_3	0.836	0.693	0.055	12.572	< .001	0.585	0.801
Character =~							
Char_1	0.471	0.426	0.069	4.698	< .001	0.190	0.463
Char_2	0.751	0.647	0.067	9.692	< .001	0.517	0.778
Char_3	0.654	0.515	0.068	7.585	< .001	0.382	0.648
Char_4	0.529	0.531	0.093	6.327	< .001	0.253	0.614
Caring =~							
Care_1	0.880	0.694	0.067	10.340	< .001	0.562	0.826
Care_2	0.587	0.445	0.067	6.640	< .001	0.313	0.576
Care_3	0.851	0.664	0.048	13.688	< .001	0.569	0.759
Connection =~							
Conn_1	0.660	0.549	0.060	9.082	< .001	0.430	0.667
Conn_2	0.755	0.667	0.055	12.091	< .001	0.559	0.775
Conn_3	0.583	0.546	0.063	8.665	< .001	0.422	0.669
Conn_4	0.558	0.447	0.063	7.054	< .001	0.323	0.571
Contribution =~							
Cont_1	0.808	1.045	0.077	13.656	< .001	0.895	1.195

Cont_2	0.772	0.819	0.067	12.147	< .001	0.687	0.951
Cont_3	0.734	0.848	0.089	9.539	< .001	0.674	1.022
Cont_4	0.581	0.705	0.085	8.253	< .001	0.538	0.873
Cont_5	0.568	0.802	0.094	8.551	< .001	0.618	0.985
Cont_6	0.589	0.827	0.091	9.100	< .001	0.649	1.006
Creativity =~							
Crea_1	0.682	0.531	0.065	8.229	< .001	0.405	0.658
Crea_2	0.799	0.635	0.044	14.572	< .001	0.549	0.720
Crea_3	0.826	0.672	0.050	13.506	< .001	0.574	0.769
Crea_4	0.788	0.626	0.048	12.901	< .001	0.530	0.721
Crea_5	0.646	0.528	0.055	9.521	< .001	0.419	0.637
Crea_6	0.673	0.562	0.057	9.933	< .001	0.451	0.673
Crea_7	0.792	0.602	0.046	13.131	< .001	0.512	0.692
Crea_8	0.866	0.701	0.057	12.203	< .001	0.589	0.814
Crea_9	0.812	0.638	0.059	10.738	< .001	0.522	0.755
Hope =~							
Hope_1	0.755	0.385	0.120	3.218	< .001	0.151	0.620
Hope_2	0.652	0.306	0.088	3.487	< .001	0.134	0.478
Hope_3	0.501	0.277	0.083	3.353	< .001	0.115	0.439
Hope_4	0.810	0.383	0.114	3.374	< .001	0.161	0.606
Hope_5	0.676	0.321	0.093	3.446	< .001	0.139	0.504
Hope_6	0.622	0.320	0.090	3.539	< .001	0.143	0.497
Hope_7	0.661	0.314	0.095	3.305	< .001	0.128	0.500
Hope_8	0.758	0.396	0.121	3.280	< .001	0.159	0.633
Hope_9	0.734	0.336	0.106	3.174	< .001	0.128	0.543
Hope_10	0.758	0.362	0.114	3.175	< .001	0.139	0.586
Hope_11	0.803	0.390	0.106	3.676	< .001	0.182	0.597
Hope_12	0.798	0.374	0.111	3.379	< .001	0.157	0.591

Life Satisfaction =~							
SWLS_1	0.875	0.768	0.085	9.061	< .001	0.602	0.934
SWLS_2	0.764	0.742	0.082	9.004	< .001	0.580	0.903
SWLS_3	0.900	0.959	0.094	10.201	< .001	0.774	1.143

Note: β = standardized coefficient; *b* = unstandardized coefficient; SE = standard error; ULCI = upper limit confidence interval; LLCI = lower limit confidence interval.

Supplementary Material I

Table I.1. Path Coefficients of the Structural Equation Model (Step 2) of the 7Cs of Positive Youth Development, Hope, and Life Satisfaction Controlling for Gender, Age, and Education Using the Overall Youth Sample (n = 1,967)

Variables	*β*	*b*	SE	z-value	*p*-value	95% ULCI	95% LLCI
Hope regressed by:							
Competence	-0.108	-0.181	0.091	-1.981	0.048	-0.360	-0.002
Confidence	0.281	0.473	0.066	7.131	< .001	0.343	0.603
Character	0.128	0.215	0.128	1.683	0.092	-0.035	0.466
Caring	0.017	0.028	0.104	0.268	0.789	-0.175	0.231
Connection	0.474	0.796	0.124	6.405	< .001	0.553	1.040
Contribution	-0.023	-0.038	0.035	-1.084	0.279	-0.108	0.031
Creativity	0.165	0.277	0.029	9.676	< .001	0.221	0.333
Age	0.002	0.002	0.024	0.063	0.950	-0.045	0.048
Gender	-0.044	-0.168	0.091	-1.842	0.065	-0.346	0.011
Education	0.059	0.191	0.080	2.386	0.017	0.034	0.347
Life Satisfaction regressed by:							
Competence	0.113	0.156	0.092	1.687	0.092	-0.025	0.337
Confidence	0.333	0.460	0.074	6.217	< .001	0.315	0.605
Character	0.303	0.418	0.145	2.877	0.004	0.133	0.703
Caring	0.118	0.162	0.117	1.389	0.165	-0.067	0.391
Connection	0.193	0.267	0.118	2.264	0.024	0.036	0.498
Contribution	0.090	0.124	0.034	3.625	< .001	0.057	0.191
Creativity	0.004	0.006	0.029	0.193	0.847	-0.052	0.063
Hope	0.321	0.264	0.032	8.315	< .001	0.202	0.326
Age	0.066	0.051	0.017	3.003	0.003	0.018	0.084
Gender	-0.010	-0.032	0.065	-0.491	0.623	-0.159	0.095
Education	0.075	0.198	0.062	3.183	< .001	0.076	0.320

Note: β = standardized coefficient; *b* = unstandardized coefficient; SE = standard error; ULCI = upper limit confidence interval; LLCI = lower limit confidence interval.

Table I.2. Path Coefficients of the Structural Equation Model (Step 2) of the 7Cs of Positive Youth Development, Hope, and Life Satisfaction Controlling for Gender, Age, and Education Using the Youth Sample from Indonesia (n = 227)

Items	*β*	*b*	SE	z-value	*p*-value	95% ULCI	95% LLCI
Hope regressed by:							
Competence	-0.359	-0.699	0.307	-2.277	0.023	-1.300	-0.097
Confidence	0.651	1.268	0.412	3.076	0.002	0.460	2.076
Character	-0.397	-0.772	0.612	-1.261	0.207	-1.972	0.428
Caring	0.293	0.570	0.462	1.234	0.217	-0.336	1.476
Connection	0.565	1.100	0.353	3.115	0.002	0.408	1.793
Contribution	0.053	0.104	0.152	0.682	0.495	-0.195	0.403
Creativity	0.291	0.567	0.175	3.240	0.001	0.224	0.909
Age	-0.047	-0.057	0.096	-0.593	0.553	-0.245	0.131
Gender	-0.008	-0.035	0.301	-0.116	0.908	-0.625	0.556
Education	0.082	0.306	0.310	0.985	0.325	-0.303	0.914
Life Satisfaction regressed by:							
Competence	-0.321	-0.480	0.372	-1.290	0.197	-1.209	0.249
Confidence	0.918	1.373	0.642	2.138	0.033	0.114	2.631
Character	-0.674	-1.007	0.774	-1.301	0.193	-2.525	0.511
Caring	0.259	0.387	0.521	0.744	0.457	-0.633	1.408
Connection	0.644	0.963	0.436	2.211	0.027	0.110	1.817
Contribution	-0.131	-0.196	0.125	-1.570	0.116	-0.441	0.049
Creativity	0.321	0.479	0.255	1.881	0.060	-0.020	0.979
Hope	0.280	0.215	0.175	1.231	0.018	0.127	0.557
Age	0.022	0.021	0.090	0.231	0.818	-0.155	0.197
Gender	0.001	0.005	0.273	0.019	0.985	-0.530	0.541
Education	0.136	0.390	0.288	1.355	0.175	-0.174	0.954

Note: *β* = standardized coefficient; *b* = unstandardized coefficient; SE = standard error; ULCI = upper limit confidence interval; LLCI = lower limit confidence interval.

Table I.3. Path Coefficients of the Structural Equation Model (Step 2) of the 7Cs of Positive Youth Development, Hope, and Life Satisfaction Controlling for Gender, Age, and Education Using the Youth Sample from Malaysia (n = 280)

Items	*β*	*b*	SE	z-value	*p*-value	95% ULCI	95% LLCI
Hope regressed by:							
Competence	-1.504	-6.077	23.179	-0.262	0.793	-0.507	0.353
Confidence	0.482	0.971	0.150	0.327	0.004	0.284	0.843
Character	0.618	2.497	8.825	0.283	0.777	-0.800	0.794
Caring	-0.094	-0.378	2.371	-0.160	0.873	-0.025	0.268
Connection	0.370	0.852	0.346	0.296	0.006	0.370	0.865
Contribution	-0.106	-0.430	1.755	-0.245	0.806	-0.870	0.009
Creativity	0.606	0.780	0.779	0.304	0.021	0.427	0.946
Age	0.094	0.228	0.494	0.461	0.645	-0.740	0.195
Gender	0.006	0.065	0.349	0.185	0.853	-0.620	0.749
Education	-0.091	-0.934	2.031	-0.460	0.646	-0.915	0.046
Life Satisfaction regressed by:							
Competence	-0.494	-0.853	6.770	-0.126	0.900	-0.122	0.416
Confidence	0.643	1.110	3.901	0.285	0.776	0.534	0.755
Character	0.014	0.025	2.519	0.010	0.992	-0.913	0.963
Caring	0.020	0.035	0.520	0.067	0.947	-0.984	0.054
Connection	0.551	0.952	4.468	0.213	0.831	0.405	0.708
Contribution	-0.106	-0.184	0.511	-0.360	0.719	-1.185	0.817
Creativity	-0.110	-0.189	0.516	-0.367	0.714	-1.201	0.822
Hope	0.213	0.291	0.951	0.096	0.024	0.174	0.456
Age	-0.087	-0.091	0.174	-0.521	0.602	-0.433	0.251
Gender	0.009	0.041	0.189	0.218	0.828	-0.329	0.411
Education	0.033	0.146	0.743	0.197	0.844	-1.311	1.603

Note: *β* = standardized coefficient; *b* = unstandardized coefficient; SE = standard error; ULCI = upper limit confidence interval; LLCI = lower limit confidence interval.

Table I.4. Path Coefficients of the Structural Equation Model (Step 2) of the 7Cs of Positive Youth Development, Hope, and Life Satisfaction Controlling for Gender, Age, and Education Using the Youth Sample from the Philippines (n = 479)

Items	*β*	*b*	SE	z-value	*p*-value	95% ULCI	95% LLCI
Hope regressed by:							
Competence	-0.174	-0.269	0.229	-1.173	0.241	-0.717	0.180
Confidence	0.437	0.674	0.124	5.440	< .001	0.431	0.917
Character	0.324	0.437	0.217	0.169	0.006	0.289	0.463
Caring	0.178	0.274	0.180	1.522	0.128	-0.079	0.628
Connection	0.348	0.536	0.220	2.440	0.015	0.106	0.967
Contribution	0.023	0.035	0.080	0.440	0.660	-0.121	0.192
Creativity	0.178	0.274	0.065	4.195	< .001	0.146	0.402
Age	0.001	0.002	0.068	0.023	0.981	-0.132	0.135
Gender	-0.026	-0.085	0.160	-0.532	0.595	-0.400	0.229
Education	0.111	0.343	0.149	2.305	0.021	0.051	0.634
Life Satisfaction regressed by:							
Competence	-0.149	-0.214	0.299	-0.715	0.475	-0.799	0.372
Confidence	0.415	0.594	0.163	3.650	< .001	0.275	0.913
Character	0.437	0.624	0.273	2.283	0.022	0.160	0.588
Caring	0.058	0.084	0.224	0.373	0.709	-0.356	0.523
Connection	0.504	0.720	0.296	2.433	0.015	0.140	1.300
Contribution	0.139	0.198	0.087	2.285	0.022	0.128	0.368
Creativity	0.097	0.138	0.081	1.717	0.086	-0.020	0.296
Hope	0.253	0.235	0.067	3.516	< .001	0.104	0.366
Age	0.026	0.030	0.057	0.531	0.595	-0.081	0.142
Gender	0.031	0.094	0.139	0.678	0.498	-0.178	0.366
Education	0.036	0.104	0.130	0.800	0.424	-0.151	0.360

Note: *β* = standardized coefficient; *b* = unstandardized coefficient; SE = standard error; ULCI = upper limit confidence interval; LLCI = lower limit confidence interval.

Table I.5. Path Coefficients of the Structural Equation Model (Step 2) of the 7Cs of Positive Youth Development, Hope, and Life Satisfaction Controlling for Gender, Age, and Education Using the Youth Sample from Singapore (n = 247)

Items	*β*	*b*	SE	z-value	*p*-value	95% ULCI	95% LLCI
Hope regressed by:							
Competence	0.073	0.188	0.208	0.903	0.366	-0.220	0.595
Confidence	0.366	0.940	0.274	3.427	0.001	0.402	1.478
Character	0.286	0.734	0.319	2.300	0.021	0.109	1.360
Caring	-0.065	-0.168	0.264	-0.634	0.526	-0.686	0.351
Connection	0.375	0.964	0.342	2.818	0.005	0.294	1.635
Contribution	-0.046	-0.117	0.149	-0.785	0.432	-0.409	0.175
Creativity	0.052	0.132	0.097	1.372	0.170	-0.057	0.322
Age	-0.037	-0.073	0.147	-0.493	0.622	-0.361	0.216
Gender	-0.059	-0.348	0.404	-0.861	0.389	-1.139	0.444
Education	-0.027	-0.139	0.359	-0.388	0.698	-0.843	0.564
Life Satisfaction regressed by:							
Competence	-0.029	-0.045	0.131	-0.341	0.733	-0.301	0.212
Confidence	0.745	1.159	0.256	4.523	< .001	0.657	0.961
Character	-0.168	-0.262	0.326	-0.804	0.421	-0.901	0.377
Caring	0.267	0.415	0.252	1.648	0.099	-0.078	0.908
Connection	-0.083	-0.130	0.244	-0.531	0.596	-0.608	0.349
Contribution	-0.068	-0.106	0.112	-0.946	0.344	-0.324	0.113
Creativity	-0.072	-0.111	0.073	-1.523	0.128	-0.254	0.032
Hope	0.172	0.104	0.091	1.140	0.004	0.075	0.283
Age	-0.017	-0.020	0.083	-0.237	0.813	-0.181	0.142
Gender	0.052	0.187	0.227	0.825	0.409	-0.257	0.632
Education	0.028	0.088	0.200	0.442	0.658	-0.303	0.480

Note: *β* = standardized coefficient; *b* = unstandardized coefficient; SE = standard error; ULCI = upper limit confidence interval; LLCI = lower limit confidence interval.

Table I.6. Path Coefficients of the Structural Equation Model (Step 2) of the 7Cs of Positive Youth Development, Hope, and Life Satisfaction Controlling for Gender, Age, and Education Using the Youth Sample from Thailand (n = 486)

Items	*β*	*b*	SE	z-value	*p*-value	95% ULCI	95% LLCI
Hope regressed by:							
Competence	-0.192	-0.309	0.305	-1.015	0.310	-0.906	0.288
Confidence	0.049	0.080	0.231	0.344	0.731	-0.373	0.533
Character	0.058	0.094	0.367	0.257	0.797	-0.624	0.813
Caring	-0.149	-0.239	0.307	-0.779	0.436	-0.841	0.363
Connection	0.830	1.336	0.482	2.772	0.006	0.391	2.281
Contribution	-0.036	-0.057	0.094	-0.606	0.545	-0.242	0.128
Creativity	0.202	0.326	0.091	3.586	< .001	0.148	0.504
Age	0.061	0.065	0.035	1.845	0.065	-0.004	0.134
Gender	-0.062	-0.250	0.132	-1.897	0.058	-0.508	0.008
Education	0.089	0.268	0.105	2.540	0.011	0.061	0.475
Life Satisfaction regressed by:							
Competence	0.288	0.384	0.280	1.371	0.170	-0.165	0.934
Confidence	0.410	0.547	0.248	2.204	0.028	0.061	1.033
Character	-0.513	-0.684	0.413	-1.658	0.097	-1.494	0.125
Caring	0.141	0.188	0.312	0.601	0.548	-0.424	0.800
Connection	0.061	0.081	0.405	0.200	0.841	-0.712	0.875
Contribution	0.086	0.115	0.088	1.308	0.191	-0.057	0.288
Creativity	-0.004	-0.005	0.087	-0.055	0.956	-0.174	0.165
Hope	0.284	0.235	0.093	2.519	0.012	0.052	0.418
Age	0.067	0.059	0.032	1.864	0.062	-0.003	0.121
Gender	0.002	0.005	0.136	0.039	0.969	-0.261	0.271
Education	0.090	0.226	0.099	2.283	0.022	0.032	0.420

Note: *β* = standardized coefficient; *b* = unstandardized coefficient; SE = standard error; ULCI = upper limit confidence interval; LLCI = lower limit confidence interval.

Table I.7. Path Coefficients of the Structural Equation Model (Step 2) of the 7Cs of Positive Youth Development, Hope, and Life Satisfaction Controlling for Gender, Age, and Education Using the Youth Sample from Vietnam (n = 248)

Items	*β*	*b*	SE	z-value	*p*-value	95% ULCI	95% LLCI
Hope regressed by:							
Competence	-0.506	-0.853	1.507	-0.566	0.571	-3.805	2.100
Confidence	0.690	1.837	1.492	1.231	0.021	0.187	0.262
Character	-1.154	-1.945	2.845	-0.684	0.494	-7.521	3.631
Caring	0.551	0.929	2.189	0.424	0.671	-3.361	5.218
Connection	0.649	1.093	1.762	0.621	0.035	0.360	0.746
Contribution	-0.101	-0.171	0.338	-0.505	0.613	-0.833	0.491
Creativity	0.564	0.952	0.448	2.123	0.034	0.273	0.830
Age	0.019	0.023	0.092	0.249	0.804	-0.158	0.204
Gender	0.042	0.156	0.232	0.670	0.503	-0.300	0.611
Education	0.036	0.140	0.296	0.472	0.637	-0.441	0.721
Life Satisfaction regressed by:							
Competence	0.446	0.591	0.695	0.849	0.396	-0.772	1.953
Confidence	0.198	0.262	0.693	0.379	0.705	-1.095	1.620
Character	-0.033	-0.043	1.122	-0.039	0.969	-2.242	2.155
Caring	0.466	0.618	0.685	0.902	0.367	-0.724	1.959
Connection	-0.621	-0.822	0.849	-0.969	0.333	-2.486	0.842
Contribution	0.116	0.153	0.184	0.833	0.405	-0.207	0.513
Creativity	-0.195	-0.259	0.306	-0.847	0.397	-0.858	0.340
Hope	0.451	0.354	0.205	1.727	0.004	0.248	0.756
Age	0.132	0.124	0.059	2.124	0.034	0.010	0.239
Gender	0.003	0.009	0.175	0.053	0.958	-0.334	0.353
Education	-0.111	-0.342	0.200	-1.707	0.088	-0.734	0.051

Note: *β* = standardized coefficient; *b* = unstandardized coefficient; SE = standard error; ULCI = upper limit confidence interval; LLCI = lower limit confidence interval.

Supplementary Material J

Table J.1. Indirect Relations of the 7Cs of Positive Youth Development on Life Satisfaction via Hope Controlling for Gender, Age, and Education Using the Overall Youth Sample (n = 1,967)

PYD Dimension	*β*	*b*	SE	z-value	*p*-value	95% ULCI	95% LLCI
Competence	-0.035	-0.048	0.025	-1.935	0.053	-0.096	0.001
Confidence	0.090	0.125	0.021	5.899	< .001	0.083	0.166
Character	0.041	0.057	0.035	1.602	0.109	-0.013	0.126
Caring	0.005	0.007	0.027	0.269	0.788	-0.046	0.061
Connection	0.152	0.210	0.040	5.277	< .001	0.132	0.288
Contribution	-0.007	-0.010	0.009	-1.092	0.275	-0.028	0.008
Creativity	0.053	0.073	0.011	6.567	< .001	0.051	0.095

Note: *β* = standardized coefficient; *b* = unstandardized coefficient; SE = standard error; ULCI = upper limit confidence interval; LLCI = lower limit confidence interval.

Table J.2. Indirect Relations of the 7Cs of Positive Youth Development on Life Satisfaction via Hope Controlling for Gender, Age, and Education Using the Youth Sample from Indonesia (n = 227)

PYD Dimension	*β*	*b*	SE	z-value	*p*-value	95% ULCI	95% LLCI
Competence	0.100	0.150	0.147	1.020	0.308	-0.138	0.439
Confidence	0.182	0.273	0.281	0.970	0.032	0.178	0.824
Character	0.111	0.166	0.234	0.708	0.479	-0.293	0.625
Caring	-0.082	-0.123	0.171	-0.716	0.474	-0.458	0.213
Connection	0.158	0.237	0.219	1.081	0.027	0.112	0.665
Contribution	-0.015	-0.022	0.032	-0.691	0.490	-0.086	0.041
Creativity	0.081	0.122	0.124	0.982	0.026	0.021	0.365

Note: *β* = standardized coefficient; *b* = unstandardized coefficient; SE = standard error; ULCI = upper limit confidence interval; LLCI = lower limit confidence interval.

Table J.3. Indirect Relations of the 7Cs of Positive Youth Development on Life Satisfaction via Hope Controlling for Gender, Age, and Education Using the Youth Sample from Malaysia (n = 280)

PYD Dimension	*β*	*b*	SE	z-value	*p*-value	95% ULCI	95% LLCI
Competence	-0.320	-0.554	4.073	-0.136	0.092	-0.537	0.430
Confidence	0.103	0.283	0.362	0.126	0.009	0.084	0.147
Character	0.132	0.228	1.772	0.128	0.098	-0.246	0.701
Caring	-0.020	-0.034	0.278	-0.124	0.401	-0.580	0.511
Connection	0.079	0.248	0.465	0.131	0.006	0.025	0.092
Contribution	-0.023	-0.039	0.285	-0.138	0.391	-0.598	0.519
Creativity	0.129	0.227	0.390	0.126	0.039	0.117	0.143

Note: *β* = standardized coefficient; *b* = unstandardized coefficient; SE = standard error; ULCI = upper limit confidence interval; LLCI = lower limit confidence interval.

Table J.4. Indirect Relations of the 7Cs of Positive Youth Development on Life Satisfaction via Hope Controlling for Gender, Age, and Education Using the Youth Sample from the Philippines (n = 479)

PYD Dimension	*β*	*b*	SE	z-value	*p*-value	95% ULCI	95% LLCI
Competence	-0.044	-0.063	0.051	-1.247	0.212	-0.162	0.036
Confidence	0.111	0.158	0.044	3.556	< .001	0.071	0.245
Character	0.082	0.103	0.051	2.168	0.007	0.042	0.109
Caring	0.045	0.064	0.043	1.495	0.135	-0.020	0.149
Connection	0.088	0.126	0.053	2.373	0.018	0.022	0.230
Contribution	0.006	0.008	0.019	0.435	0.663	-0.029	0.045
Creativity	0.045	0.064	0.021	3.119	0.002	0.024	0.105

Note: *β* = standardized coefficient; *b* = unstandardized coefficient; SE = standard error; ULCI = upper limit confidence interval; LLCI = lower limit confidence interval.

Table J.5. Indirect Relations of the 7Cs of Positive Youth Development on Life Satisfaction via Hope Controlling for Gender, Age, and Education Using the Youth Sample from Singapore (n = 247)

PYD Dimension	*β*	*b*	SE	z-value	*p*-value	95% ULCI	95% LLCI
Competence	0.013	0.020	0.028	0.699	0.485	-0.035	0.074
Confidence	0.063	0.098	0.083	1.184	0.023	0.064	0.160
Character	0.049	0.076	0.078	0.973	0.330	-0.077	0.230
Caring	-0.011	-0.017	0.035	-0.503	0.615	-0.085	0.050
Connection	0.064	0.100	0.101	0.996	0.031	0.037	0.198
Contribution	-0.008	-0.012	0.019	-0.656	0.512	-0.049	0.024
Creativity	0.009	0.014	0.016	0.880	0.379	-0.017	0.044

Note: *β* = standardized coefficient; *b* = unstandardized coefficient; SE = standard error; ULCI = upper limit confidence interval; LLCI = lower limit confidence interval.

Table J.6. Indirect Relations of the 7Cs of Positive Youth Development on Life Satisfaction via Hope Controlling for Gender, Age, and Education Using the Youth Sample from Thailand (n = 486)

PYD Dimension	*β*	*b*	SE	z-value	*p*-value	95% ULCI	95% LLCI
Competence	-0.054	-0.073	0.082	-0.888	0.375	-0.233	0.088
Confidence	0.014	0.019	0.054	0.348	0.727	-0.086	0.124
Character	0.017	0.022	0.089	0.248	0.804	-0.153	0.197
Caring	-0.042	-0.056	0.080	-0.705	0.481	-0.213	0.100
Connection	0.235	0.314	0.173	1.815	0.007	0.125	0.454
Contribution	-0.010	-0.013	0.023	-0.585	0.558	-0.059	0.032
Creativity	0.057	0.077	0.039	1.954	< .001	0.042	0.153

Note: *β* = standardized coefficient; *b* = unstandardized coefficient; SE = standard error; ULCI = upper limit confidence interval; LLCI = lower limit confidence interval.

Table J.7. Indirect Relations of the 7Cs of Positive Youth Development on Life Satisfaction via Hope Controlling for Gender, Age, and Education Using the Youth Sample from Vietnam (n = 248)

PYD Dimension	*β*	*b*	SE	z-value	*p*-value	95% ULCI	95% LLCI
Competence	-0.228	-0.302	0.515	-0.586	0.558	-1.312	0.708
Confidence	0.311	0.651	0.823	0.791	0.042	0.361	0.263
Character	-0.520	-0.689	1.308	-0.527	0.598	-3.252	1.874
Caring	0.248	0.329	0.900	0.365	0.715	-1.435	2.093
Connection	0.292	0.387	0.612	0.632	0.027	0.113	0.588
Contribution	-0.046	-0.060	0.144	-0.420	0.674	-0.342	0.221
Creativity	0.254	0.337	0.234	1.443	0.014	0.121	0.595

Note: *β* = standardized coefficient; *b* = unstandardized coefficient; SE = standard error; ULCI = upper limit confidence interval; LLCI = lower limit confidence interval.

Supplementary Material K

To evaluate an alternative conceptual ordering of the variables, we estimated an additional structural equation model in which hope was specified as the predictor variable, the 7Cs of positive youth development (PYD) were specified as mediators, and life satisfaction was specified as the outcome variable, while controlling for age, gender, and educational attainment. This alternative specification was informed by prior work suggesting that hopeful future expectations may contribute to the development of PYD characteristics (Schmid et al., 2011). The model demonstrated acceptable overall fit to the data, χ^2 (1136) = 14,253.25, $p < .001$, CFI = .948, TLI = .950, RMSEA = .077, 90% CI (.076, .078), SRMR = .072.

Despite adequate global fit, inspection of the structural parameters revealed several theoretically problematic findings. Specifically, character ($\beta = -.31$, $p < .001$), caring ($\beta = -.17$, $p < .001$), contribution ($\beta = -.11$, $p < .001$), and creativity ($\beta = -.06$, $p < .001$) demonstrated significant negative direct associations with life satisfaction after accounting for hope and the other PYD dimensions. These findings contradict both the PYD literature and the observed positive bivariate correlations among these variables. Prior research has consistently documented positive associations between PYD attributes and indicators of well-being, including life satisfaction (Carvalho et al., 2024; Holsen et al., 2017). The emergence of negative coefficients in the multivariate model likely reflects statistical suppression arising from treating hope as the predictor of the 7Cs of PYD.

Additionally, as shown in Table K, several indirect effects from hope to life satisfaction via specific PYD dimensions exhibited unexpected negative directions. While competence ($\beta = .11$, $p < .001$; 90% CI [.07, .25]), confidence ($\beta = .23$, $p < .001$; 90% CI [.26, .39]), and connection ($\beta = .18$, $p < .001$; 90% CI [.12, .40]) yielded positive indirect effects, character ($\beta = -.24$, $p < .001$; 90% CI [-.50, -.21]), caring ($\beta = -.10$, $p < .001$; 90% CI [-.21, -.09]), contribution ($\beta = -.04$, $p < .001$; 90% CI [-.09, -.03]), and creativity ($\beta = -.03$, $p < .01$; 90% CI [-.08, -.02]) produced significant negative indirect effects. From a PYD perspective, these results are counterintuitive because they imply that higher levels of several developmental strengths would be associated with lower life satisfaction.

Together, these findings suggest that although the alternative model achieved acceptable statistical fit with the data, it yielded parameter estimates that were inconsistent with the PYD framework and prior empirical evidence. Consequently, we retained the hypothesized model in which the 7Cs function as intrapersonal (competence, confidence, and creativity), interpersonal (connection and caring), and ecological (character and contribution) developmental assets that promote hope, which subsequently contributes to greater life satisfaction. This ordering is also more closely aligned with the integrative paradigm of PYD (Buenconsejo & Datu, 2022), which conceptualizes the 7Cs as relatively stable psychosocial resources, and with research identifying hope as a future-oriented motivational mechanism through which developmental assets facilitate positive well-being outcomes (Ismailov, 2026).

Table K. Indirect Relations of Hope on Life Satisfaction via the 7Cs of Positive Youth Development Controlling for Gender, Age, and Education Using the Overall Youth Sample (Alternative Structural Equation Modelling)

PYD Dimension	*β*	*b*	SE	z-value	*p*-value	95% ULCI	95% LLCI
Competence	0.109	0.159	0.046	3.435	< 0.001	0.068	0.249
Confidence	0.224	0.327	0.034	9.480	< 0.001	0.259	0.394
Character	-0.244*	-0.355	0.074	-4.834	< 0.001	-0.500	-0.211
Caring	-0.102*	-0.148	0.029	-5.113	< 0.001	-0.205	-0.092
Connection	0.177	0.258	0.073	3.554	< 0.001	0.116	0.401
Contribution	-0.042*	-0.061	0.015	-4.184	< 0.001	-0.090	-0.032
Creativity	-0.031*	-0.046	0.016	-2.936	0.003	-0.076	-0.015

Note: *β* = standardized coefficient; *b* = unstandardized coefficient; SE = standard error; ULCI = upper limit confidence interval; LLCI = lower limit confidence interval; *The emergence of several significant negative indirect effects is inconsistent with both PYD theory and the observed positive bivariate associations among the variables, suggesting possible suppression effects when hope was treated as the predictor variable of the 7Cs of PYD.

References:

Buenconsejo, J., & Datu, J. A. D. (2022). Towards an integrative paradigm of positive youth development: Implications for research, practice, and policy. *Human Development, 66* (6), 381–396. https://doi.org/10.1159/000527122

Carvalho, M., Branquinho, C., Moraes, B., Cerqueira, A., Tomé, G., Noronha, C., Gaspar, T., Rodrigues, N., & Matos, M. G. D. (2024). Positive youth development, mental stress and life satisfaction in middle school and high school students in Portugal: Outcomes on stress, anxiety, and depression. *Children, 11*(6), 681. https://doi.org/10.3390/children11060681

Schmid, K. L., Phelps, E., Kiely, M. K., Napolitano, C. M., Boyd, M. J., & Lerner, R. M. (2011). The role of adolescents' hopeful futures in predicting positive and negative developmental trajectories: Findings from the 4-H Study of Positive Youth Development. *Journal of Positive Psychology, 6*(1), 45–56. https://doi.org/10.1080/17439760.2010.536777

Holsen, I., Geldhof, J., Larsen, T., & Årdal, E. (2017). The 5Cs of positive youth development in Norway: Assessment and associations with positive and negative outcomes. *International Journal of Behavioral Development, 41*(5), 559–569. https://doi.org/10.1177/0165025416645668Hair

Ismailov, M. (2026). Life skills and hope in times of polycrisis: a developmental perspective on youth agency and future orientation. *Frontiers in Developmental Psychology, 4*. https://doi.org/10.3389/fdpys.2026.1793402
